# Was wissen wir über schulische Lehr-Lern-Prozesse im Distanzunterricht während der Corona-Pandemie? – Evidenz aus Deutschland, Österreich und der Schweiz

**DOI:** 10.1007/s11618-021-01000-z

**Published:** 2021-03-04

**Authors:** Christoph Helm, Stephan Huber, Tina Loisinger

**Affiliations:** 1 Institut für Bildungsmanagement und Bildungsökonomie IBB, Pädagogische Hochschule Zug, Zugerbergstrasse 3, 6300 Zug, Schweiz; 2 Johannes Kepler Universität Linz, Linz, Österreich

**Keywords:** Covid-19, Distanzunterricht, Homeschooling, Systematic Review, Surveys, Covid-19, Distance education, Homeschooling, Systematic Review, Surveys

## Abstract

Der Review gibt einen systematischen Überblick über den quantitativen Forschungsstand zur Schulsituation und zum Lehren und Lernen während der Corona-Pandemie. Der Review umfasst 97 Online-Befragungen, die in der Zeit vom 24. März 2020 bis 11. November 2020 durchgeführt wurden und 255.955 Fälle (Schüler*innen, Eltern, Lehrkräfte, Schulleitungen u. a.) erfassten. Die Analyse und Synthese der Befunde erfolgt entlang zweier Modelle, dem Phasenmodell des Forschungsprozesses und einem integrativen Modell zur Distance Education. Der Review macht deutlich, dass zentrale Aspekte des Lehrens und Lernens während den coronabedingten Schulschließungen im Frühjahr 2020, wie bspw. Merkmale des Fernunterrichts (z. B. Qualitätsdimensionen), Schülermerkmale (z. B. Selbständigkeit) und Merkmale der häuslichen Ressourcen für das Lernen (z. B. die elterliche Unterstützung), bereits Gegenstand vieler Befragungen waren. Die Schulsituation während der Corona-Pandemie stellt daher kein unerforschtes Phänomen mehr dar. Vielmehr fordert das wissenschaftliche Ethos von Forscher*innen dieses Feldes, den aktuellen Forschungsstand in ihren Arbeiten zu berücksichtigen. Der vorgelegte Review soll diese Aufgabe erleichtern, indem nicht nur die existierenden Befragungen gelistet, sondern deren zentrale Erkenntnisse synthetisiert werden. Darüber hinaus liefert der Review eine relevante Informationsbasis für Entscheidungen und für das Handeln in den jeweiligen Verantwortungsbereichen der Politik, Verwaltung und Schulpraxis. Gleichzeitig warnt der Review vor einer unreflektierten Übernahme der Befunde, indem die wissenschaftliche Qualität der Befragungen kritisch diskutiert wird.

**Zusatzmaterial online:**

Zusätzliche Informationen sind in der Online-Version dieses Artikels (10.1007/s11618-021-01000-z) enthalten.

## Abstract (en)

The review provides a systematic overview of the state of quantitative research on teaching and learning characteristics during school closures due to corona. The review comprises 97 online surveys conducted between 24th of March 2020 and 11th of November 2020 and covering 255,955 cases (students, parents, teachers, school leaders etc.). The analysis and synthesis of the findings was carried out along two models, the phase model of the research process and the “integrative model on distance education”. The review makes clear that central aspects of teaching and learning during corona-based school closures in Spring 2020, such as distance learning characteristics (e.g. quality dimensions), student characteristics (e.g. self-sufficiency) and characteristics of home resources for learning (e.g. parental support) have already been the object of many surveys. The school situation during the corona pandemic is therefore no longer an unexplored phenomenon. Rather, the scientific ethos of researchers in this field demands that the current state of research needs to be considered in their work. The review presented here is intended to facilitate this task by not only listing the existing surveys, but also synthesizing their central findings. In addition, the review provides a relevant information basis for decisions and action in politics, administration and school practice. At the same time, the review warns against an unreflected adoption of the findings by critically discussing the scientific quality of the surveys.

## Einleitung

Aktuell erleben wir eine durch die Corona-Pandemie ausgelöste gesellschaftliche Krise mit weitreichenden Auswirkungen auf nahezu alle gesellschaftlichen Bereiche, so auch auf das Bildungssystem. Diese wirkte sich auf alle formalen und non-formalen Bildungssettings, -stätten und -institutionen aus. Der vorliegende Beitrag fokussiert die Schule. Von Mitte März bis Mitte Mai bzw. Anfang/Mitte Juni 2020 waren die Schulen in Deutschland, in Österreich und in der Schweiz geschlossen. Danach gab es – je nach Kanton in der Schweiz und Bundesland in Deutschland und Österreich – unterschiedliche Strategien der Schulwiedereröffnung (bspw. Unterricht im Rotations- oder Schichtbetrieb, Unterricht in Halb- oder Ganzklassen). Diese für alle neue Situation führte rasch zu neuen Herausforderungen, vielen offenen Fragen und je nach Akteursgruppe zu unterschiedlichen Informationsbedürfnissen. Das Bedürfnis nach neuem Wissen wurde von einer Vielzahl von Bildungsforscher*innen und außerwissenschaftlichen Akteuren (z. B. Elternverbänden) erkannt. Viele von ihnen reagierten rasch, sodass mittlerweile 97 Befragungen vorliegen (Stand 11. November 2020), die die Schulsituation während (und nach) der coronabedingten Schulschließung im Frühjahr 2020 untersuchen.

Der vorliegende Beitrag zielt daher darauf ab, die bestehenden Befragungen zum Fernunterricht während der Schulschließung in Deutschland, Österreich und der Schweiz zu sammeln und entlang des *Modells zum Forschungsprozess* (Review 1) und des integrativen *Rahmenmodells zum Fernunterricht *(Review 2) zu analysieren und zu synthetisieren. Im Ergebnis soll ein theoriegeleiteter und strukturierter Überblick über die Fülle an vorliegenden empirischen Informationen zur Praxis des Fernunterrichts im DACH-Raum entstehen. Die Wahl der Analysemodelle liegt in ihrem methodischen und inhaltlichen Fokus begründet. Während das Review 1 entlang des Forschungsprozesses einen Blick auf Aspekte des Designs der bestehenden Befragungen erlaubt, ermöglicht Review 2 entlang des integrativen Rahmenmodells zum Fernunterricht einen fokussierten und theoriegeleiteten Blick auf Befunde zu zentralen Aspekten des Lehr-Lern-Prozesses im Fernunterricht.

## Theorie- und Forschungsstand

Ziel dieses Reviews ist es, die bestehenden Befragungen zum Fernunterricht entlang des methodischen Vorgehens sowie entlang ihrer inhaltlichen Befunde zu analysieren und zu synthetisieren. Für die Analyse des methodischen Vorgehens eignet sich die in der Literatur weithin etablierte Vorstellung des *Phasenmodells des Forschungsprozesses* (bspw. Schnell et al. 2018). Dieses Modell umfasst Kategorien, die für das Verständnis der Anlage einer Studie relevant sind (z. B. Forschungsziele/-frage, theoretischer Rahmen, Erhebungsverfahren/-instrumente, Stichprobeninformationen). Deutlich weniger klar ist die Modellwahl für die Analyse und Synthese der inhaltlichen Befunde. Da der Fernunterricht aufgrund der Schulschließungen eine gänzlich neue Situation darstellt, wurden für die Beschreibung dieses „Phänomens“ noch keine Theoriemodelle entwickelt. Es ist daher notwendig, auf Modelle aus „verwandten“ Forschungstraditionen wie dem „Homeschooling“ (Ray 2017), der „Distance Education“ (Keegan 1986; Moore 2013; Wedemeyer 1981) und der „Hausaufgabenpraxis“ (Kohler 2011; Trautwein et al. 2006) zurückzugreifen.

### Theoriemodelle verwandter Bezugsdisziplinen

#### Homeschooling

Seit dem ersten Schul-Lockdown im Frühjahr 2020 verwenden Medien, Bildungspolitiker*innen, Praktiker*innen und die allgemeine Öffentlichkeit, aber auch Bildungsforscher*innen, den Begriff „Homeschooling“, um auf die Situation während der Schulschließungen hinzuweisen. Auch wenn der Begriff „Homeschooling“ (häusliche Beschulung) die Situation treffend beschreibt, so darf nicht vergessen werden, dass der Homeschooling-Begriff ursprünglich eine amerikanische – mittlerweile weltweite – Tradition des häuslichen Unterrichts beschreibt, die sich in mehreren, sehr zentralen Punkten von der Schulsituation während der Corona-Pandemie unterscheidet. Im traditionellen (US-amerikanischen) Homeschooling …

- entscheiden sich Eltern aus unterschiedlichen Motiven (z. B. Unzufriedenheit mit dem Angebot öffentlicher Schulen, mangelnde Sicherheit an öffentlichen Schulen, religiöse Motive; siehe Redford et al. 2017) *freiwillig* für den häuslichen Unterricht. Während des Schul-Lockdowns im DACH-Raum blieb den Eltern dagegen keine Wahl.
- übernehmen Eltern die vollständige Verantwortung und Gestaltung der Beschulung bzw. (Aus‑)Bildung ihrer Kinder; wobei in der Regel individuelle Lernpläne jährlich von den Bildungsbehörden genehmigt werden müssen (Ray 2020). Während des Schul-Lockdowns im DACH-Raum blieb dagegen die Beschulung in der Verantwortung des Staates bzw. der Schulbehörden.
- existieren beide Formen der Beschulung (häuslicher und öffentlicher Unterricht) parallel. Im Lockdown wurde der traditionelle öffentliche Unterricht in einen häuslichen, aber (in unterschiedlichem Ausmaß) aus der Ferne organisierten Unterricht umgestellt.

Der Umstand, dass (a) das traditionelle (US-amerikanische) Homeschooling nicht mit jenem während der Corona-Pandemie vergleichbar ist und (b) unseren Recherchen nach bisher keine eigenständige Theorie zum Homeschooling entwickelt wurde – vielmehr werden gängige Bildungs- und Lerntheorien und -philosophien, z. B. die Montessori-Pädagogik, übernommen –, legt nahe, dass die Forschungstradition zum „Homeschooling“ kein passendes Theoriemodell für die Analyse und Synthese der hier untersuchten Befragungen zum Fernunterricht während Corona bereithalten kann.

#### Distance Education

Vielversprechender erscheint die Tradition der Forschung zur Distance Education, da die angeführten Unterscheidungsmerkmale zwischen Homeschooling und coronabedingtem Fernunterricht hier nicht gelten: (1) Bildungsangebote in der Distance Education sind meist alternativlos, d. h. dasselbe Bildungsprogramm wird häufig nicht in Präsenz angeboten. (2) Die Verantwortung für die Gestaltung des Bildungsangebotes bleibt weiterhin bei privaten oder öffentlichen Institutionen und wird nicht von den Eltern übernommen. Aber auch die Tradition der Distance Education hat Merkmale, die eine direkte Umlage auf die Schulsituation während des coronabedingten Lockdowns erschweren. So hat sie ihren Ursprung in der Erwachsenenbildung, was vermutlich der Grund dafür ist, dass der Rolle der elterlichen Unterstützung im Lernprozess keine Bedeutung beigemessen wird. Dennoch erscheint ein Blick auf existierende Theorien zur Distance Education zielführend.

Keegan (1986), Simonson et al. (1999) und Saba (2016) geben einen Überblick über in Summe sechs etablierte Theoriemodelle zur Distance Education^1^, wovon drei Theorien Bezüge zu Lehr- und Lern-Prozessmerkmalen aufweisen, die auch für den Fernunterricht relevant sind.^2^ Während Wedemeyer (1981) und Moore (2013) Distance Education über den Grad an ermöglichter Individualisierung, Autonomie und Selbstständigkeit definieren, lenkt Holmberg (2003) den Blick auf die persönlichen Beziehungen und motivationalen Aspekte („Empathy“) als zentrale Elemente der Distance Education. Gerade für den coronabedingten Fernunterricht ist anzunehmen, dass die Selbstständigkeit der Schüler*innen und ihre Motivation besonders relevant für die Initiierung und Aufrechterhaltung von Lernbemühungen sind. Daher bieten diese Theorien gute Anknüpfungspunkte was die Rolle des selbstgesteuerten Lernens betrifft. Allerdings gehen diese Theorien nicht über jene hinaus, die bereits in die traditionellen Lehr-Lern-Forschung Eingang gefunden haben (z. B. Selbstbestimmungstheorie), sodass wir an dieser Stelle nicht näher auf sie eingehen.

Etwas aktuellere theoretische Rahmenmodelle mit Bezug zum Distance Learning finden sich im Bereich der Online Education (Multimodal Model for Online Education, Picciano 2017) und im Bereich des E‑Learnings (E-Learning Systems’ Theoretical Framework, Aparicio et al. 2016). Picciano (2017) leitet aus mehreren Lerntheorien (z. B. Behaviorismus und Kognitivismus) zentrale Qualitätselemente der Online Education ab: „Content“, „Social/Emotional“, „Self-Paced“, „Dialectic/Questioning“, „Evaluation/Assessment“, „Collaboration“, „Reflection“ sowie „Learning Community“. Damit fokussiert das Modell stark auf Aktivitäten und Methoden des Lehrens und Lernens und auf die Frage, welche Möglichkeiten der Einsatz von Technologien bietet, um diese Aktivitäten und Methoden im Online-Setting umzusetzen. Damit liefert es Anhaltspunkte für relevante Qualitätselemente des coronabedingten Fernunterrichts. Eine deutlich allgemeinere und breitere Theorie als die bisher skizzierten Theorien liefern Aparicio et al. (2016). Sie werfen einen systemischen Blick auf das E‑Learning. Die von ihnen beschriebenen Elemente des E‑Learning-Systems umfassen neben Merkmalen der Schüler*innen und der Qualität des Lehr-Lern-Prozesses auch Merkmale der Lehrpersonen und Merkmale der digitalen Medien/Technologien. Das Modell weist Ähnlichkeiten zum Angebots-Nutzungs-Modell (Helmke 2009) auf: Es systematisiert zentrale Einflussbereiche/Dimensionen des E‑Learnings, bleibt aber in seiner konkreten Auslegung und seinen Wirkungszusammenhängen untereinander unklar.

Alle bisher identifizierten Theoriemodelle zur Distance Education sind für sich alleine genommen für die Analyse der Befragungen zum coronabedingten Fernunterricht unzureichend, da sie wichtige Protagonist*innen des Lehr-Lern-Prozesses des coronabedingten Fernunterrichts vernachlässigen: Theorien zum Homeschooling beinhalten naturgemäß keine Aussagen zur Rolle der Schule und Lehrpersonen. Theorien zur Distance Education wiederum beziehen die für den Fernunterricht so zentrale häusliche/familiäre Situation der Kinder und Jugendlichen nicht mit ein. Modelle, die alle drei Protagonist*innen (Lehrer*innen, Schüler*innen und Eltern) in den Blick nehmen, sind in der Forschungstradition zur *Hausaufgabenpraxis* (z. B. Kohler 2011; Trautwein et al. 2006) zu finden.

#### Hausaufgabenpraxis

Theoriemodelle der Hausaufgabenpraxis postulieren, dass die Rolle der Eltern im Rahmen der Hausaufgabenbetreuung sowie die häusliche Situation/häusliche Ressourcen allgemein (z. B. sozioökonomischer Status der Lernenden, Ausstattung zuhause) starken Einfluss auf die Qualität und den Erfolg häuslicher Lernprozesse nimmt. Zwei in der Literatur prominente Modelle sind das Homework-Modell (Trautwein et al. 2006) und das Prozessmodell zur Wirkungsweise von Hausaufgaben (Kohler 2011).

Das *Homework-Modell* von Trautwein et al. (2006) basiert u. a. auf verschiedenen Motivationstheorien, insbesondere der Erwartungs-Wert-Theorie, und gängigen Lehr-Lern-Theorien, insbesondere der Angebots-Nutzungs-Logik.^3^ Im Unterschied zum Angebots-Nutzungs-Modell versuchen Trautwein et al. (2006) die für die Hausaufgabenpraxis relevanten Faktorenbündel „Elternrolle“, „Schülermotivation“, „Qualität der Hausaufgabenpraxis“ und „Hausaufgabenverhalten der Schüler*innen“ konkreter zu beschreiben. So postulieren Trautwein et al. (2006), dass Merkmale der Lernumgebung, der Lehrpersonen, der Hausaufgabenpraxis, der Schüler*innen, der Eltern und der elterlichen Lernunterstützung bei der Hausaufgabenbearbeitung die Lernmotivation der Lernenden beeinflussen. Die Motivation wiederum wird als Prädiktor des Hausaufgabenverhaltens der Schüler*innen angenommen, welches mit der Schülerleistung assoziiert ist. Mit seiner starken Ähnlichkeit zum Angebots-Nutzungs-Modell bleibt das Homework-Modell mit Bezug zur Hausaufgabenpraxis notwendigerweise abstrakt.

Kohler (2011) legt dagegen ein *Prozessmodell* vor, das – ebenfalls eingebettet in das Angebots-Nutzungs-Modell – zentrale aufeinander aufbauende, didaktische Schritte des Einsatzes und der Nutzung von Hausaufgaben stärker ausdifferenziert: Auswahl, Vergabe, Bearbeitung, Kontrolle und Auswertung von Hausaufgaben. Das Modell zeigt zwei für den Lernprozess relevante Punkte auf:

1. Es verweist auf die Gefahr, dass eine erfolgreiche Hausaufgabenbearbeitung und damit ein erfolgreicher Lernprozess nach jedem Schritt vorzeitig abgebrochen werden kann. Die Erfahrung zeigt, dass nicht jede erteilte Hausaufgabe auch von den Schüler*innen bearbeitet wird; und dass nicht jede bearbeitete Hausaufgabe auch von Lehrkräften kontrolliert (und ausgewertet) wird.
2. Unterschiedliche Prozessschritte haben unterschiedliche Funktionen und Qualitätsmerkmale. Während im Rahmen der Auswahl von Hausaufgaben die Funktion der Hausaufgabe und fachdidaktische Überlegungen im Vordergrund stehen, stehen bei Erteilung von Hausaufgaben Fragen der Klarheit und der Zeitnutzung im Fokus. Kontrolle bezieht sich auf die Überprüfung des Vorhandenseins der Ausarbeitung; während die Auswertung das inhaltliche Feedback meint.

Diese beiden für das Prozessmodell konstitutiven Aspekte erscheinen auch für die Aufgabenpraxis im Fernunterricht wesentlich.

#### Ein integratives Modell zu Lehr-Lern-Prozessen während der Schulschließungen

**Figure 1:**
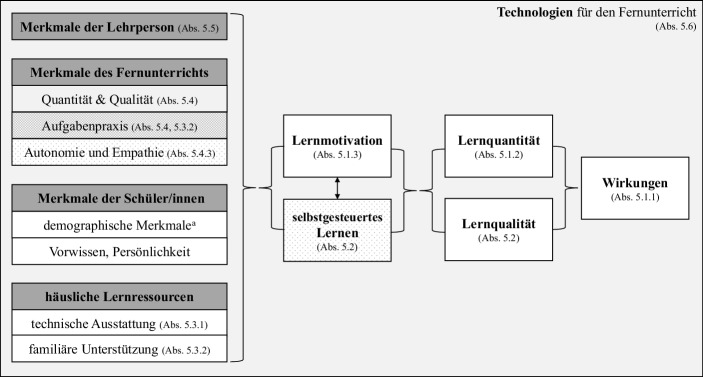

Modelle der Hausaufgabenforschung scheinen aufgrund ihrer Angebots-Nutzungs-Logik alle wesentlichen Faktorenbündel für den Fernunterricht abzudecken. Was ihnen fehlt, ist der Blick auf die Rolle der Technik im Lehr-Lern-Prozess. Daher wird in Abb. 1 ein integratives Modell vorgelegt, das das Modell der Hausaufgabenpraxis nach Trautwein et al. (2006; keine Schattierung) um das Prozessmodell der Hausaufgabenpraxis (Schachfelder-Schattierung) sowie Theorien der Distance Education (punktierte Schattierung) und e‑Education (hellgraue Schattierung) erweitert.

### Forschungsstand verwandter Bezugsdisziplinen

#### Forschungsstand zum Homeschooling

Aufgrund der parallelen Existenz des Homeschoolings als Alternativangebot zum öffentlichen Unterricht, hat sich die Mehrzahl der empirischen Forschung zum Homeschooling auf folgende Fragen konzentriert (Ray 2017):

- Wer sind die Homeschoolers? Welche demographischen Merkmale (z. B. Wohnort, Bildungsabschluss, ethnische Herkunft, religiöse Einstellung) charakterisieren sie?
- Welche Motive bewegen Eltern, ihre Kinder zuhause zu unterrichten?
- Unterscheiden sich Homeschoolers und öffentlich unterrichtete Schüler*innen in
  - ihren akademischen Leistungen?
  - ihrem Sozialverhalten?
  - langfristigen Bildungserfolgen (meist GPA an tertiären Bildungseinrichtungen)?

Keine dieser Forschungsfragen (und ihrer Befunde) deckt sich mit den Intentionen der Befragungen zur Schulsituation während des coronabedingten Lockdowns. Daher sind auch die Befunde unserer Ansicht nach für die vorliegende Arbeit kaum relevant. Ein sehr globaler und vorsichtiger Schluss, der aus der Review-Studie von Ray (2017) abgeleitet werden kann, ist, dass Homeschooler in den USA in vielen Untersuchungen höhere akademische Leistungen und ein angemesseneres Sozialverhalten zeigten, was nahelegt, dass häuslicher Unterricht per se nicht mit negativen Schüleroutcomes einhergehen muss. Aus den oben angeführten Gründen ist es aber kaum zulässig, diese Befunde auf die Corona-Situation umzulegen.

#### Forschungsstand zur Distance Education und zur Online bzw. E-Education

Eine Vielzahl aktueller Meta-Analysen zur Distance Education (Allen et al. 2004; Bernard et al. 2004; Cavanaugh 2001; Shachar und Neumann 2010) und dem Online-Learning (Castro und Tumibay 2019; Ebner und Gegenfurtner 2019; Means et al. 2013; Pei und Wu 2019) zeigt, dass Distance Education leichte Vorteile hinsichtlich des Lernerfolgs bietet. Allerdings basiert die Mehrheit der Studien auf Stichproben der post-sekundären (Aus‑)Bildung. Nur wenige Meta-Analysen untersuchen auch den Pflichtschulbereich. Cavanaugh (2001) und Bernard et al. (2004) finden leichte positive Effekte für Distance Education bei K‑12-Schüler*innen; aber auch hohe Heterogenität in den Befunden. Aktuellere Meta-Analysen (Means et al. 2013) können dagegen keine Unterschiede in akademischen Leistungen zwischen K‑12-Schüler*innen im Online-Unterricht und K‑12-Schüler*innen im traditionellen Unterricht beobachten.

#### Forschungsstand aus weiteren Bezugsdisziplinen

Tomasik et al. (2020) geben einen Überblick zu Befunden aus Forschungstraditionen, die sich mit den Auswirkungen reduzierter oder fehlender Unterrichtszeit auf Schülerleistungen beschäftigen. Unterrichtszeit kann aus unterschiedlichen Gründen variieren oder gänzlich ausfallen; etwa aufgrund von Schülerfehlstunden, Schulferien und Schulschließungen bedingt durch Naturkatastrophen. Darüber hinaus variiert die Unterrichtszeit zwischen Bildungssystemen mit unterschiedlichen Stundentafeln (Curricula). Für all diese Aspekte liegen empirische Befunde aus einschlägigen Forschungstraditionen (School Absenteeism Studies, Seasonal Learning Studies, Studies on School Closures During Natural Disasters, Comparative Studies on Instructional Time) vor. Tomasik et al. (2020) resümieren, dass Studien zu Schülerfehlstunden und zu Schulschließungen aufgrund von Naturkatastrophen schwache bis starke negative Effekte auf die Leistungsentwicklungen von Schüler*innen beobachten konnten, während Studien zu Effekten von Schulferien und von zwischen den Bildungssystemen variierenden Unterrichtszeiten kaum nennenswerte Unterschiede in den Schülerleistungen feststellen konnten. Ein weiterer Aspekt, der die Lernzeit von Schüler*innen deutlich beeinflussen kann, ist die Hausaufgabenpraxis. Eine Reihe von Meta-Analysen dieser Forschungstradition (Cooper et al. 2006; Fan et al. 2017) belegt positive, aber nur schwache Effekte der Hausaufgabenpraxis auf Schülerleistungen.

Für den vorgelegten Review sind diese Befunde allerdings nur bedingt relevant, da die bisherigen Befragungen zum coronabedingten Fernunterricht nicht die akademischen Leistungen der Kinder und Jugendlichen erfassen.^4^ Vielmehr fokussieren die Befragungen darauf, wie die Umsetzung und die Rahmenbedingungen des Fernunterrichts von unterschiedlichen Akteursgruppen wahrgenommen werden. Dazu existieren unseren Recherchen zufolge keine Reviews. Dies ist daher Ziel des vorliegenden Beitrags.

## Design des Reviews

### Identifikation der Befragungen

Die Identifikation der vorliegenden 97 Befragungen erfolgte zweistufig: Einerseits wurde ein systematischer Literaturreview durchgeführt. Darüber hinaus wurden andererseits Befragungen durch Google-Recherche und durch direktes Anschreiben der Autor*innen von im Internet angekündigten Befragungen (z. B. https://airtable.com/shrQFS0CG3jdPf725/tblbgmyj6f8HAiKYo) identifiziert. Beide Vorgehensweisen erfolgten entlang folgender Einschlusskriterien: Die Befragungen mussten …

- *quantitative* Erhebungen darstellen.
- sich inhaltlich auf die *deskriptive Beschreibung der Schulsituation bzw. des Fernunterrichts und/oder des Distance Learnings* während der Schulschließungen im Frühjahr 2020 fokussieren.
- Stichproben enthalten, die sich aus *Akteursgruppen des Bildungssystems* (Schüler*innen, Lehrer*innen, Schulleitungen, Eltern, Schuladministration etc.) mit Bezug zur *Primarstufe, Sekundarstufe I oder II (exkl. der Berufsbildung)* in *Deutschland, Österreich oder der Schweiz* zusammensetzen.

Entsprechend sind qualitative Studien, Studien, die Fragen ohne Bezug zum Fernunterricht (z. B. Ausbreitung des Virus in Schulen) untersuchen, internationale Befragungen über den DACH-Raum hinaus, Befragungen im Elementar‑, Hochschul- oder Berufs‑, Aus‑, Fort- und Weiterbildungsbereich nicht Teil des Reviews. Auch Studien zur objektiv erfassten Leistungsentwicklung der Schüler*innen während des Lockdowns sind nicht Inhalt des Reviews.

Kein Ausschlusskriterium stellt der „Forschungsstatus“ der Institution, von der die Befragung durchgeführt wurde, dar. So umfasst das Review auch 22 Befragungen, die von Organisationen durchgeführt wurden, die üblicherweise nicht forschend tätig sind (z. B. Elternverbände, Plattformen für Lehrerfortbildungen). Wir haben uns aus zwei Gründen entschlossen diese Befragungen aufzunehmen: (1) Die von diesen Befragungen erfasste Stichprobe ist substanziell (*N* = 76.624). (2) Das Untersuchungsdesign gleicht jenen von Forschungsinstitutionen (z. B. Online-Befragung). Zudem handelt es sich beim coronabedingten Fernunterricht um ein neues Forschungsfeld, für das bisher keine empirische Evidenz vorlag. Gerade in einer solchen frühen Phase der Untersuchung eines gänzlich neuen Phänomens erscheint es gerechtfertigt, auch auf Befragungen „nicht wissenschaftlichen Ursprungs“ zurückzugreifen. Umso wichtiger ist eine kritische Reflexion der Befragungen vor dem Hintergrund wissenschaftlicher Kriterien, die wir in Abschn. 6.2 vornehmen.

### Recherche- und Sichtungsprotokoll des systematischen Reviews

Die systematische Recherche von Befragungen zum Fernunterricht erfolgt in vier Forschungsdatenbanken, von denen zu erwarten ist, dass sie in Summe alle für das Reviewziel relevante Veröffentlichungen beinhalten: Google Scholar, Scopus, EBSCOhost, FIS Bildung. Tab. 1 enthält die je nach Datenbank verwendeten Suchbegriffe, Einstellungsoptionen und Anzahl der Suchtreffer sowie die Anzahl der letztlich als relevant identifizierten Befragungen.

**Table 1:** 

Datenbank	Suchbegriffe & logische Operatoren	Gewählte Optionen der Abfrage	Suchtreffer	Relevant
Google Scholar	(corona OR „covid-19“ OR „cov-19“) AND (school OR schooling OR instruction OR learning) AND (survey) AND (Germany OR Austria OR Switzerland) AND (students OR pupils) -medical -therapy	2020–2021, gibt nur die nach Zitaten relevantesten 1000 Treffer aus	2240	10
Google Scholar	(Corona OR „covid-19“ OR „cov-19“) AND (Schule OR Unterricht OR Lernen) AND (Deutschland OR Österreich OR Schweiz) AND (Umfrage OR Befragung) AND (Schüler OR Lehrer)	2020–2021, gibt nur die nach Zitaten relevantesten 1000 Treffer aus	421	16
Scopus	(TITLE-ABS-KEY (corona OR covid OR covid-19 OR coronavirus OR 2019-ncov) AND TITLE-ABS-KEY (school OR schooling OR instruction OR learning) AND TITLE-ABS-KEY (germany OR austria OR switzerland)) AND (LIMIT-TO (PUBYEAR, 2021) OR LIMIT-TO (PUBYEAR, 2020)) AND (LIMIT-TO (SUBJAREA, „PSYC“) OR LIMIT-TO (SUBJAREA, „SOCI“) OR LIMIT-TO (SUBJAREA, „MULT“))	2020–2021, im Titel und Abstract, Beschränkung auf die Disziplinen Psychologie (PSYC), Soziologie (SCI) und Multidisziplinarität (MULT)	62	3
EBSCOhost	(corona OR covid OR covid-19 OR coronavirus OR 2019-ncov) AND (school OR schooling OR instruction OR learning) AND (Germany OR Austria OR Switzerland)	2020–2021, im Abstract	104	2
FIS Bildung	CORONA ODER COVID-19 UND Umfrage	2020–2021, im Freitext	15	6
Summe	–	–	2842	37
Nach Dubletten	–	–	–	25

Zur Identifizierung relevanter Befragungen in den Suchtreffern wurde dreistufig vorgegangen: Im ersten Schritt wurde geprüft, ob der Titel Hinweise für den Ausschluss enthält (wie bspw. „higher education“, Länder). War dies nicht der Fall, wurde im zweiten Schritt Entsprechendes für das Abstract geprüft. War auch hier kein Ausschlussgrund identifizierbar, wurde in einem dritten Schritt der Volltext (sofern zugänglich) auf Passung zum Reviewziel geprüft. Schritt 1 und 2 führten nach Bereinigung von Dubletten zu 25 Suchtreffern, von denen im Rahmen des dritten Prüfungsschrittes weitere fünf Treffer ausgeschlossen wurden. Tab. 2 weist die als relevant identifizierten Befragungen aus.

**Table 2:** 

Datenbank	Titel	Autor*innen
GS Deutsch	Kinder, Eltern und ihre Erfahrungen während der Corona-Pandemie	Andresen et al. (2020)
FIS Bildung	Schulschließungen wegen Corona: Regelmäßiger Kontakt zur Schule kann die schulischen Aktivitäten der Jugendlichen erhöhen	Anger et al. (2020)
GS Deutsch	Wie erlebten Jugendliche den Corona-Lockdown? Ergebnisse einer Befragung im Kanton Zürich	Baier und Kamenowski (2020)
GS Englisch	Social inequality in the homeschooling efforts of German high school students during a school closing period	Dietrich et al. (2021)
Scopus	Large loss in studying time during the closure of schools in Switzerland in 2020	Grätz und Lipps (2021)
GS Englisch	COVID-19 and Educational Inequality: How School Closures Affect Low-and High-Achieving Students	Grewenig et al. (2020)
GS Deutsch	Lernen in Zeiten der Corona-Pandemie. Die Rolle familiärer Merkmale für das Lernen von Schüler*innen. Befunde vom Schul-Barometer in Deutschland, Österreich und der Schweiz	Huber und Helm (2020b)
Scopus	COVID-19 and schooling: evaluation, assessment and accountability in times of crises – reacting quickly to explore key issues for policy, practice and research with the school barometer	Huber und Helm (2020a)
GS Deutsch	COVID-19 und aktuelle Herausforderungen in Schule und Bildung. Erste Befunde des Schul-Barometers in Deutschland, Österreich und der Schweiz	Huber et al. (2020)
GS Deutsch	COVID-19: Strategien der Schulentwicklung in der Krise	Jesacher-Rößler und Klein (2020)
GS Deutsch	Teachers’ experiences of stress and their coping strategies during COVID-19 induced distance teaching	Klapproth et al. (2020)
GS Deutsch	Kindsein in Zeiten von Corona	Langmeyer et al. (2020)
GS Englisch	Energetic students, stressed parents, and nervous teachers: A comprehensive exploration of inclusive homeschooling during the COVID-19 crisis	Letzel et al. (2020)
GS Deutsch	Fernunterricht als Ausnahmesituation. Befunde einer bundesweiten Befragung von Eltern mit Kindern in der Grundschule	Porsch und Porsch (2020)
GS Englisch	COVID-19 LehrerInnenbefragung-Zwischenergebnisse: Was tun, damit aus der Gesundheitskrise nicht auch eine Bildungskrise wird?	Steiner et al. (2020)
GS Deutsch	Lernen trotz Corona. Chancen und Herausforderungen des distance learning an österreichischen Schulen	Tengler et al. (2020)
FIS Bildung	‚Sind doch Corona-Ferien, oder nicht?‘ Befunde einer Schüler*innenbefragung zum ‚Fernunterricht‘	Wacker et al. (2020)
GS Deutsch	Bundesweite Elternbefragung zu Homeschooling während der Covid 19-Pandemie. Erkenntnisse zur Umsetzung des Homeschoolings in Deutschland	Wildemann und Hosenfeld (2020)
GS Deutsch	Bildung in der Coronakrise: Wie haben die Schulkinder die Zeit der Schulschließungen verbracht, und welche Bildungsmaßnahmen befürworten die Deutschen?	Wößmann et al. (2020)
FIS Bildung	Leben und Lernen in Zeiten von Corona	Ziegler und Hannemann (2020)

*GS* Google Scholar

### Überblick zu den identifizierten Befragungen

**Figure 2:**
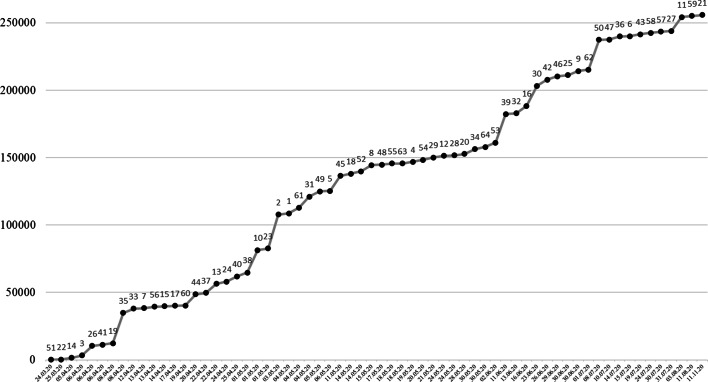

Tab. 3 listet die im Rahmen der systematischen Literaturrecherche als auch der darüber hinausgehenden Recherchen *identifizierten 64 Quellen* (meist online verfügbare PDF-Reports) auf, in denen die Befunde der 97 Befragungen berichtet werden.^5^ In bemerkenswerter Weise schafften es bildungswissenschaftliche und außerwissenschaftliche Institutionen innerhalb von nur wenigen Monaten, umfassendes Wissen zu unterschiedlichsten Fragestellungen im Zusammenhang mit dem Fernunterricht aufzubauen. Fasst man alle Befragungen zusammen, so wurden bereits rund 256.000 Fälle^6^ (Schüler*innen, Eltern, Lehrkräfte, Schulleitungen) aus Deutschland, Österreich und der Schweiz befragt. Abb. 2 zeigt, wie sich der Informationsstand in den Monaten nach Beginn der Schulschließungen Mitte März entwickelt hat. Viele weitere Befragungen befanden sich zum Zeitpunkt der Manuskripterstellung in der Planung, Durchführung oder Auswertung (siehe bspw. https://airtable.com/shrQFS0CG3jdPf725/tblbgmyj6f8HAiKYo für eine Übersicht zu *ongoing studies* in der Schweiz).

**Table 3:** 

		Erfasste Fälle						Stichprobe	Befragungszeitraum		Rek	Rep
#	Autor*innen	Lehrer*innen^a^	Schüler*innen	Eltern	Schulleitung	SV & US	Summe	Geographischer Raum	Beginn	Ende		
1	ADAS und LIFE (2020)	–	241	633	–	–	874	DE	14.04.20	04.05.20	MV	Nein
2	Andresen et al. (2020)	–	–	25.224	–	–	25.224	DE	24.04.20	03.05.20	MV	Nein
3	Anger et al. (2020) Dietrich et al. (2021)	**–**	1735	**–**	**–**	**–**	1735	DE	24.03.20	06.04.20	MV	kA
4	Baier und Kamenowski (2020)	–	1103	–	–	–	1103	Zürich	23.04.20	19.05.20	MV	Nein
5	Berghammer (2020)	**–**	–	230	–	–	230	AT	01.05.20	06.05.20	OP	gD
6	Bešić et al. (2020)	142	–	–	–	–	142	AT	15.06.20	15.07.20	kA	Nein
7	Bezirkselternausschuss Mitte (2020)	–	–	459	–	–	459	Berlin	06.04.20	13.04.20	MV	Nein
8	Bildungsdirektion Nidwalden (2020)^b^	432	1569	2506	38	–	4545	Nidwalden	04.05.20	15.05.20	MV	kA
9	Cattaneo und Wolter (2020)	–	–	3000	–	–	3000	CH	01.06.20	30.06.20	OP	Ja
10	Cecchini und Dutrévis (2020)^c^ Huber und Helm (2020b)	2719	5634	8241	93	–	16.687	Genf	17.04.20	01.05.20	MV	Nein
11	Civey (2020)	–	–	10.277	–	–	10.277	DE	22.05.20	03.08.20	kA	Ja
12	Conus und Durler (2020)	–	–	1280	–	–	1280	Waadt, Freiburg	28.04.20	24.05.20	MV +	Nein
13	Cordes (2020)	3767	–	2867	–	–	6634	DE	22.04.20	22.04.20	kA	Nein
14	Dreer et al. (2020)	1263	–	–	–	–	1263	Thüringen	30.03.20	05.04.20	MV +	Nein
15	Eickelmann und Drossel (2020)	310	–	–	–	–	310	DE	02.04.20	14.04.20	OP	Ja
16	LEARN EPFL (2020)	5666	–	–	–	–	5666	Waadt	21.04.20	16.06.20	MV	Nein
17	Feistritzer et al. (2020)	–	–	316	–	–	316	Wien	10.04.20	17.04.20	kA	Nein
18	fobizz (2020)	1695	–	–	–	–	1695	DE, AT, CH	01.05.20	14.05.20	kA	Nein
19	forsa (2020c)	1031	–	–	–	–	1031	DE	02.04.20	08.04.20	OP	Ja
20	forsa (2020a)	1006	–	–	–	–	1006	DE	22.05.20	28.05.20	OP	Ja
21	forsa (2020b)	–	–	–	785	–	785	DE	13.10.20	11.11.20	OP	Ja
22	Grote (2020)	–	–	122	–	–	122	DE	23.03.20	25.03.20	MV	Nein
23	Grütter et al. (2020)	–	1159	–	–	–	1159	Deutschschweiz, Tessin	30.03.20	01.05.20	kA	Nein
24	Heller und Zügel (2020)	–	1016	586	–	–	1602	DE	19.04.20	24.04.20	OP	Ja
25	Holtgrewe et al. (2020a)	88	343	417	–	–	848	AT	01.04.20	30.06.20	kA	Nein
26	Huber et al. (2020)^d^ Huber und Helm (2020a)^d^	1949	2152	2222	655	138	7116	DE, AT, CH	24.03.20	06.04.20	MV +	Nein
27	Jesacher-Rößler und Klein (2020)	–	–	–	532	–	532	AT	01.06.20	31.07.20	MV +	Nein
28	Klapproth et al. (2020)	380	–	–	–	–	380	DE	20.04.20	24.05.20	MV +	Nein
29	Kugelmeier und Schmolze-Krahn (2020)	–	–	1767	–	–	1767	DE	14.05.20	21.05.20	MV +	Nein
30	Landeselternrat Sachsen (2020)	–	–	14.731	–	–	14.731	Sachsen	27.05.20	23.06.20	MV +	Nein
31	Langmeyer et al. (2020)	–	–	8127	–	–	8127	DE	22.04.20	04.05.20	MV +	Nein
32	Letzel et al. (2020)	124	150	247	–	–	521	DE	27.04.20	13.06.20	MV	Nein
33	Lochner (2020)	–	–	3107	–	–	3107	Thüringen	01.04.20	12.04.20	MV +	Nein
34	Lorenz et al. (2020b) Lorenz et al. (2020a)	3632	–	–	–	–	3632	DE	16.04.20	30.05.20	kA	Nein
35	Müller (2020)	–	–	22.507	–	–	22.507	Hamburg	25.03.20	08.04.20	MV	Nein
36	Neuenschwander et al. (2020)	108	1321	851	52	–	2332	CH	18.06.20	14.07.20	kA	kA
37	Neumeier (2020) brlv (2020)	1100	–	–	–	–	1100	Bayern	14.04.20	22.04.20	kA	Ja
38	Petersen und Heimbach (2020)	–	–	2776	–	–	2776	DE	01.05.20	01.05.20	OP	Ja
39	Piatti et al. (2020)	2741	–	18.385	–	–	21.126	Tessin	02.06.20	11.06.20	kA	kA
40	Porsch und Porsch (2020)	–	–	3995	–	–	3995	DE	25.03.20	25.04.20	MV +	Nein
41	Rathgeb (2020)	–	1002	–	–	–	1002	DE	02.04.20	06.04.20	OP	Ja
42	Refle et al. (2020) Grätz und Lipps (2021)	–	3410	1305	–	–	4715	CH	13.05.20	26.06.20	MV	kA
43	Samson und Dukes (2020)	–	–	1511	–	–	1511	DE, AT, CH	09.04.20	19.07.20	kA	kA
44	Schober et al. (2020a)	–	8349	–	–	–	8349	AT	06.04.20	20.04.20	MV +	Nein
45	Schober et al. (2020c)	–	11.118	–	–	–	11.118	AT	27.04.20	11.05.20	MV +	Nein
46	Schober et al. (2020b)	–	2491	–	–	–	2491	AT	08.06.20	29.06.20	MV +	Nein
47	Schreiner et al. (2020)	–	234	–	–	–	–	Tirol	29.06.20	10.07.20	kA	Nein
48	Schütz und Bestgen (2020)	429	–	–	–	–	429	DE	07.04.20	17.05.20	kA	Nein
49	Schwab et al. (2020)	3467	263	286	–	–	4016	AT	23.04.20	05.05.20	MV	Nein
50	Schwerzmann und Frenzel (2020)	3691	15.785	2374	172	–	22.022	Luzern	08.06.20	08.07.20	OP	Ja
51	Seda und Ottacher (2020)	110	–	–	–	–	110	AT	23.03.20	24.03.20	MV	Nein
52	Spiel und Holzer (2020)	1759	–	–	–	–	1759	AT	01.05.20	14.05.20	MV +	Nein
53	Steiner et al. (2020)	3274	–	–	–	–	3274	AT	04.05.20	02.06.20	kA	Nein
54	Steinmayr und Christiansen (2020) Steinmayr et al. (2020)	–	–	1456	–	–	1456	DE	01.04.20	20.05.20	kA	Nein
55	Tengler et al. (2020) Schrammel et al. (2020)	417	–	404	12	–	833	AT	01.04.20	18.05.20	MV +	Nein
56	Thies und Klein (2020)	–	–	1067	–	–	1067	DE	03.04.20	13.04.20	OP	kA
57	Trültzsch-Wijnen und Trültzsch-Wijnen (2020)	–	433	510	–	–	943	AT	15.07.20	30.07.20	kA	kA
58	Vuorikari et al. (2020)^e^	–	413	513	–	–	926	DE	22.07.20	24.07.20	kA	kA
59	Vuorikari et al. (2020)^f^	–	378	484	–	–	862	CH	21.07.20	11.08.20	kA	kA
60	Wacker et al. (2020)	–	169	–	–	–	169	Baden-Württemberg	09.04.20	19.04.20	MV	Nein
61	Wildemann und Hosenfeld (2020)	–	–	4230	–	–	4230	DE	08.04.20	04.05.20	kA	Nein
62	Wößmann et al. (2020)	–	–	1099	–	–	1099	DE	03.06.20	01.07.20	OP	gD
63	Ziegler und Hannemann (2020)	50	–	40	–	–	90	DE	02.04.20	18.05.20	MV +	Nein
64	Zinn und Bayer (2020)	–	–	1508	–	–	1508	DE	01.04.20	30.05.20	OP	Ja
–	Summe *N*	41.350	60.468	151.660	2339	138	255.955	–	–	–	–	–

Während des Begutachtungsprozesses des Manuskripts sind wir auf folgende weitere Befragungen gestoßen, die wir aber aufgrund des fortgeschrittenen Status des Manuskripts nicht mehr in die Analysen des Reviews aufnehmen konnten: Attig et al. (2020), Lockl et al. (2020), Wolter et al. (2020), Scheiber et al. (2020), Schönherr und Zandonella (2020), S-CLEVER-Konsortium (2021) und Zoch et al. (2020). Darüber hinaus verweisen wir auf die Übersicht von Projekten bei Fickermann und Edelstein (2021).

*SV & US* Vertreter*innen der Schulverwaltung und der Unterstützungssysteme, *AT* Österreich, *CH* Schweiz, *DE* Deutschland, *MV* E-Mail-Verteiler, *MV* *+* E-Mail-Verteiler, (Soziale) Netzwerke, Internetseiten, *OP* Online-Panel, *kA* keine Angaben, *Rek.* Rekrutierung der Stichprobe, *Rep.* Repräsentativität der Stichprobe, *gD* gewichtete Daten

^a^Neben Lehrer*innen wurden in manchen Befragungen auch weitere Mitarbeitende der Schule (z. B. Erzieher*innen in Ganztagsschulen und Schulsozialarbeiter*innen) erfasst

^b^Die Rücklaufquote beträgt für Schulleitungen in Volksschulen 72 %, Lehrkräfte 35 % und Schüler*innen 8 %

^c^Die Rücklaufquote beträgt für Schulleitungen 100 %, Lehrkräfte 84 %, Schüler*innen 67 % und Eltern 65 %

^d^In diesen Befragungen wird explizit ausgewiesen, dass neben Lehrer*innen auch weitere Mitarbeitende der Schule befragt wurden

^e^Für Deutschland wurde die Studie von Claudia Lampert (Leibniz-Institut für Medienforschung | Hans-Bredow-Institut) durchgeführt

^f^Für die Schweiz wurde die Studie von Lilian Suter und Gregor Waller (ZHAW Zürcher Hochschule für Angewandte Wissenschaften) durchgeführt

### Kategorienbildung

Die Oberkategorien für die Analyse und Synthese der identifizierten 97 Befragungen wurden aus dem Phasenmodell zum Forschungsprozess (Abschn. 4: Review 1) und dem integrativen Distance Education-Modell (Abschn. 5: Review 2) deduktiv abgeleitet. Die Dokumente „A1 Codierungen Review 1“ und „A2 Codierungen Review 2“ im Online-Zusatzmaterial listen alle Befragungen und Kategorien samt Codierungen. Das Kategorienmodell für den Review 2 wurde allerdings induktiv bei Analyse des Datenmaterials angepasst. D. h., Kategorien, die in den Befunden nicht vorkamen, wurden entfernt. Dies betraf insbesondere Schülermerkmale wie das Vorwissen und Persönlichkeitsmerkmale, Aspekte der Lernqualität sowie Aspekte, die dem Prozessmodell der Hausaufgabenpraxis nach Kohler (2011) zuzuordnen sind. Auch wurden bei sehr umfangreichen Kategorien Subkategorien induktiv aus dem Datenmaterial abgeleitet. Dies betraf vor allem die Qualitätsmerkmale des Fernunterrichts (Abschn. 5.4). Im Online-Zusatzmaterial befindet sich das Dokument „A3 Codier-Manual Review 2“ mit Informationen zur Kategoriendefinition und Ankerbeispielen.

### Hinweise zur Darstellung der Befunde

In Abschn. 5 werden die deskriptiven Befunde der identifizierten Befragungen gegenübergestellt. Im Rahmen der Synthese werden die Befunde zu sehr ähnlichen Fragestellungen zusammengefasst. Aufgrund der unterschiedlichen Operationalisierung sehr ähnlicher Konstrukte, der unterschiedlichen Stichprobenzusammensetzung, der verschiedenen Befragungszeiträume, der unterschiedlichen Antwortformate etc. ist jedoch Vorsicht bei der Interpretation geboten. Am Ende jedes Unterkapitels in Abschn. 5 werden Unterschiede in den Befunden nach dem sozioökonomischen Hintergrund der Schüler*innen und nach dem Schultyp dargestellt. Diese Befunde zu Gruppenunterschieden wurden in allen Befragungen auf Basis deskriptiver Verteilungsanalysen durchgeführt. Auf Tests zur statistischen Absicherung der Unterschiede wurde in den Befragungen bisweilen verzichtet.

## Review 1: Beschreibung der Befragungen entlang des Forschungsprozesses

Die Analyse und Synthese der Designs der 97 Befragungen zum Fernunterricht erfolgt entlang des Phasenmodells zum Forschungsprozess, das u. a. folgende für das Design einer Studie relevanten Kategorien postuliert: Forschungsziel, theoretische Annahmen, Operationalisierung der interessierenden Konstrukte, Art der Erhebung, Stichprobe, Analysemethoden. Da Befragungen zum Fernunterricht nicht nur von Forschungseinrichtungen durchgeführt wurden, wird auch die Kategorie „durchführende Organisation“ aufgenommen. Darüber hinaus interessiert aufgrund des hohen öffentlichen Interesses an den Befunden und der damit verbundenen sehr raschen Veröffentlichung der Befragungen die Kategorie „Dissemination“.

### Durchführende Organisationen

Von den 97 Befragungen wurde die Mehrheit (75 Befragungen) von Organisationen durchgeführt, die üblicherweise wissenschaftlich tätig sind. 32 Befragungen wurden von Universitäten (darunter 4 Befragungen, im Rahmen derer Befragungsinstitute mit der Datenerhebung beauftragt wurden), 13 von Pädagogischen Hochschulen oder Fachhochschulen, 8 von kommerziellen Befragungsinstituten und 22 von öffentlichen Forschungsinstituten durchgeführt. Darüber hinaus existieren 22 Befragungen, die von Organisationen durchgeführt wurden, die üblicherweise *nicht* forschend tätig sind. Acht Befragungen wurden von Kantonen (darunter vier, die über Befragungsinstitute beauftragt wurden) durchgeführt. Drei Befragungen stammen von Eltern-, zwei von Lehrerverbänden und zwei von einem Bundesverband für Kinder- und Jugendförderung. Jeweils zwei Befragungen wurden von einer Beratungsstelle und einer Lernplattform durchgeführt. Schließlich stammt jeweils eine Befragung von einer Lehrerplattform und von einem Wissenschaftsmagazin.

### Ziele der Befragungen

Alle Befragungen weisen einen sehr stark ausgeprägten situationsanalytischen Charakter auf. Damit ist gemeint, dass sie primär deskriptive Ziele, nämlich die Beschreibung der aktuellen Situation aus Sicht unterschiedlicher Akteursgruppen, verfolgen. So wurden die Befragungen durchgeführt, um bspw. „Einblick in das Funktionieren der Fernbeschulung [zu] erhalten“ (Müller 2020, S. 4), „mehr darüber [zu] erfahren, wie Schüler*innen mit dieser Situation zurechtkommen“ (Schober et al. 2020a) oder um zu erheben „welche Erfahrungen mit homeschooling in Zeiten von Corona gemacht wurden“ (Tengler et al. 2020, S. 4). Huber et al. (2020) beziehen sich in der Erfassung eines Stimmungsbildes durch das Schul-Barometer auf das Konzept von Responsible Science, in dem die Wissenschaft Fragestellungen oder Problemlagen aus der Gesellschaft aufgreift, mit wissenschaftlicher Methodologie bearbeitet und Ergebnisse an die entsprechenden Akteursgruppen, zum Beispiel verantwortliche Führungskräfte auf den verschiedenen Ebenen in Politik, Verwaltung und Praxis zurückmeldet. Da in den meisten Ergebnisberichten die Befragungsziele, wenn überhaupt, sehr vage dargestellt sind, bleibt unklar, inwiefern die jeweilige Befragung auch den Anspruch verfolgt, wissenschaftlichen Zielen und Qualitätskriterien zu genügen. Letztere sind vermutlich für Befragungen wissenschaftsnaher Organisationseinheiten eher anzunehmen.

### Theorie

In den meisten Fällen wurden keine Informationen über die theoretischen Konzepte gefunden, die der jeweiligen Befragung zugrunde liegen. Dort wo Bezüge zu Theorien hergestellt werden, sind diese meist knapp und/oder sehr abstrakt gehalten. So verweisen bspw. Schober et al. (2020a, 2020b, 2020c) in ihren Ergebnisberichten auf die Konzepte der sozialen Eingebundenheit und des selbstregulierten Lernens. Wildemann und Hosenfeld (2020) ziehen das Angebots-Nutzungs-Modell nach Helmke heran, um ihre Befunde zur Lernmotivation von Kindern zu rahmen. Mit Fokus auf die Belastung von Lehrkräften und Eltern stützen sich Dreer et al. (2020) auf das Job-Demands-Resources Model (nach Bakker und Demerouti) und Porsch und Porsch (2020) auf das Stressmodell (nach Lazarus). Schließlich verweisen Huber et al. (2020) auf eine breite Palette von Forschungssträngen und Konzepten (darunter die school effectiveness research oder das Modell der Basisdimensionen guten Unterrichts), um die an unterschiedliche Akteursgruppen gerichteten Befragungen in traditionelle Forschungsfelder einzubetten. Aufgrund des Wunsches nach zeitnaher Rückmeldung der Befunde ist nachvollziehbar, dass in den Darstellungen Theoriebezüge weniger stark ausgeführt werden. Ein weiterer Grund könnte darin liegen, dass viele Befragungen deskriptive Ziele (Stichwort: Situationsanalyse) und nicht traditionelle wissenschaftliche Ziele, wie die Testung von Hypothesen verfolgen. Jedenfalls ist wünschenswert, in weiteren Publikationen die theoretischen Grundlagen expliziter anzuführen.

### Konstruktoperationalisierung

Soweit aus den vorliegenden Informationen ersichtlich ist, erfassten nahezu alle Befragungen die interessierenden Konstrukte (z. B. die erlebte Belastung der Schüler*innen) häufig mit nur einem Item; vermutlich um die Länge der Onlinebefragung möglichst kurz zu halten (vgl. bspw. Huber et al. 2020, S. 18). In wenigen Ausnahmefällen wurden (etablierte) Skalen eingesetzt. So wurden bspw. in der Befragung von Dreer et al. (2020) Lehrer-Skalen zu den selbsteingeschätzten Kompetenzen im Umgang mit digitalen Medien (Brandhofer 2015), zur Arbeitszufriedenheit (Ho und Au 2006) und Belastung (Cohen et al. 1983) sowie zu den erlebten psychologischen Grundbedürfnissen (Broeck et al. 2010) eingesetzt. Weiters setzten Porsch und Porsch (2020) in ihrer Elternbefragung Skalen zur Erfassung des Beanspruchungserlebens (Richter et al. 2015) und der Selbstwirksamkeit der Eltern (Wendt et al. 2017) ein. Bei Letzel et al. (2020) wurde die Taxonomie der Binnendifferenzierung nach Pozas et al. (2020) eingesetzt. In diesen wenigen Studien ist es daher möglich im Rahmen der Datenauswertung für den Messfehler zu kontrollieren. Entsprechend werden auch nur bei Dreer et al. (2020) und Porsch und Porsch (2020) Reliabilitätsmaße berichtet.

**Figure 3:**
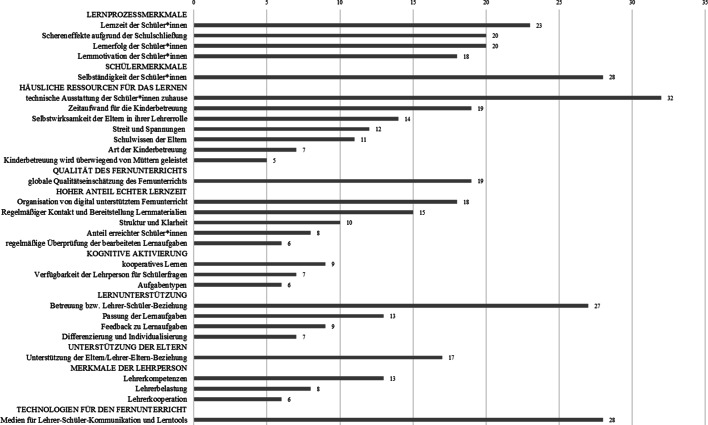

In einer vorbereitenden Analyse wurden die Items der Befragungen nach Themenbereichen geclustert. Abb. 3 zeigt jene Themenbereiche bzw. Forschungsfragen, die am häufigsten in den 64 Studienberichten (Tab. 3) adressiert wurden. Die Themenbereiche wurden entlang des in Review 2 als Analyseraster verwendeten integrativen Theoriemodells zum Fernunterricht geclustert.

### Erhebung

#### Erhebungsmethode

Alle Befragungen stellen Online-Befragungen – in seltenen Ausnahmen wie Zinn und Bayer (2020) auch Telefoninterviews dar. Die Rekrutierung der befragten Personen erfolgte auf sehr unterschiedliche Weise:

- In 20 Ergebnisberichten werden keine Informationen zur Rekrutierung der befragten Personen angeführt.
- 16 Ergebnisberichte führen an, dass die Rekrutierung über die Versendung des Links zur Online-Befragung via E‑Mailverteiler (eigene Datenbanken, Elternverbände, Schulleiterverbände etc.) und über Rundbriefe mit der Bitte um Weiterleitung an verschiedene Akteursgruppen (insb. Lehrpersonen, Eltern und Schüler*innen) erfolgte.
- Zusätzlich wurde in 15 Ergebnisberichten explizit die Bewerbung des Links zur Online-Befragung über verschiedene Medien und soziale Netzwerke wie Facebook und andere Netzwerke (z. B. Schulleiterverbänden) angeführt.
- 11 Projekte greifen auf existierende (Online‑)Panels zurück.
- Zwei Projekte nutzen das sogenannte River-Sampling über Webseiten.

#### Erhebungszeitraum

Tab. 3 und Abb. 2 zeigen das Datum, zu dem die Erhebung der jeweiligen Befragung endete. Da in Österreich die Schulen am 18. Mai und Deutschland je nach Bundesland zwischen dem 25. Mai und dem 15. Juni^7^ für alle Schüler*innen öffneten, endeten manche Befragungen zu einem Zeitpunkt, an dem die Kinder bzw. Jugendlichen die Schule zumindest nach dem Rotationsprinzip bereits wieder besuchten. Ein Teil der Befragungen wurde gänzlich nach der Schulwiedereröffnung durchgeführt. Viele der Befragungen, die nach dem Lockdown durchgeführt wurden, erfassten die Schulsituation während der Schulschließung retrospektiv. Einige Befragungen fokussierten dagegen Themen der Schulwiedereröffnung (forsa 2020a; Kugelmeier und Schmolze-Krahn 2020; Landeselternrat Sachsen 2020; Schober et al. 2020b).

In Tab. 4 skizzieren wir ein Modell, das hilft, gegenwärtige und künftige Befragungen und Publikationen den zeitlichen Phasen des Schulsystems während der Corona-Pandemie zuzuordnen. Dies erscheint uns wichtig, da die unterschiedlichen Phasen mit unterschiedlichen schulischen Angeboten (z. B. Schicht‑/Rotationsmodell, Halb‑/Ganzklassenmodell während der Phase der Schulwiedereröffnung) und unterschiedlicher Nutzung dieser Angebote durch die Schüler*innen (z. B. Summer Schools in den Ferien) einhergehen. Gleichzeitig ist zu berücksichtigen, dass auch innerhalb der Phasen das Angebot und die Nutzung geografisch variierten (z. B. Angebote in der Phase der Ferien).

**Table 4:** 

Schuljahr 2019/20					Schuljahr 2020/21		
Vor Corona	Schulschließung		Schule vor den Ferien	Ferien	Das neue Schuljahr		
Beginn des Schuljahres bis Mitte März 2020	Frühe Schulschließung (z. B. nur Stoffwiederholung bis zu Ostern)	Späterer Verlauf der Schulschließung (z. B. Vermittlung von neuem Unterrichtsstoff)	Halb- und Ganzklassenmodell, Schicht- und Rotationsmodell …	Mit und ohne Zusatzangebote in bestimmten Regionen (z. B. Summer School in Österreich)	Beginn des Schuljahres	Ende des Kalenderjahres	Weiterer Verlauf des Schuljahres

#### Messwiederholung

Mehrere Forschungsinitiativen führten eine Follow-up-Befragung während und/oder nach der Schulschließung durch (Civey 2020; Dreer et al. 2020; forsa 2020c, 2020a, 2020b; Huber et al. 2020; Huber und Helm 2020b; Müller 2020; Schober et al. 2020a, 2020b, 2020c). In den meisten Fällen scheint es sich dabei aber um eine von der vorangegangenen Befragung weitgehend unabhängige Befragung zu handeln. Welche Befragungen echte Längsschnittanalysen erlauben, geht nicht aus den Ergebnisberichten hervor.^8^

### Stichprobe

#### Umfang

Tab. 3 zeigt, dass die bis zum 11. November 2020 durchgeführten 97 Befragungen bereits 255.955 erfasste Fälle umfassen – davon 60.468 Schüler*innen, 151.660 Eltern und 41.350 Lehrkräfte sowie 2339 Schulleitungen. Eine Studie (Huber et al. 2020) befragte darüber hinaus auch 138 Personen der Schulaufsicht und Unterstützungssysteme. Die dem Stichprobenumfang nach größten Befragungen sind die deutschlandweite Familienstudie von Andresen et al. (2020), die Hamburger Elternbefragung von Müller (2020) und die jeweils mehrere Zielgruppen umfassenden, kantonalen Befragungen von Schwerzmann und Frenzel (2020) und Piatti et al. (2020).

#### Länder

Die Anzahl der Befragungen teilt sich wie folgt nach den DACH-Ländern auf: 40 (Deutschland), 23 (Österreich), 27 (Schweiz), 7 (DACH). Die erfassten Fälle verteilen sich wie folgt auf die Länder: 124.485 (Deutschland), 36.651 (Österreich), 84.497 (Schweiz), 10.322 (DACH).

#### Stichprobenzusammensetzung und Repräsentativität

In nur jeweils 7 der 23 Schülerbefragungen bzw. 6 der 26 Lehrerbefragungen werden vollständige Informationen zu Geschlecht, Alter und Schultyp der befragten Personen angeführt. Bei den Elternbefragungen werden nur in 4 bzw. 2 von 39 Befragungen Informationen zum Geschlecht und Alter bzw. Geschlecht, Alter und dem Schultyp der Kinder berichtet. Vor dem Hintergrund der meist sehr spärlichen *Informationen zu den Stichproben* lässt sich keine zusammenfassende Darstellung der in den 97 Befragungen untersuchten Stichproben erstellen. Dies stellt insbesondere für die Einordnung und Interpretation der in Abschn. 5 berichteten Befunde einen unbefriedigenden Zustand dar. Allerdings stehen diesem Umstand 18 Befragungen (in folgenden Berichten: Berghammer 2020; Cattaneo und Wolter 2020; Civey 2020; Eickelmann und Drossel 2020; forsa 2020c, 2020a, 2020b; Heller und Zügel 2020; Neumeier 2020; Petersen und Heimbach 2020; Rathgeb 2020; Schwerzmann und Frenzel 2020; Wößmann et al. 2020; Zinn und Bayer 2020)^9^ gegenüber, die *Repräsentativität* der erhobenen Daten beanspruchen. Meist verweisen die Autor*innen der Befragungen auf die repräsentative Zusammensetzung der Stichprobe durch a priori festgelegte Quoten (meist Alter, Geschlecht, Schulstufe/Schulart, Bundesland/Region, Schulabschluss, Erwerbsstatus, Gemeindegrößen). Nach Stand des vorliegenden Reviews greifen zwei Befragungen (Berghammer 2020; Wößmann et al. 2020) auf gewichtete Daten zurück, um für die deutsche bzw. österreichische Wohnbevölkerung repräsentative/verallgemeinerbare Aussagen tätigen zu können.

### Analysemethoden

Alle Befragungen verwenden primär deskriptive Statistiken. Die Antworten der befragten Personen werden meist in Form absoluter und/oder relativer Häufigkeiten sowie in Form von Mittelwerten (für unterschiedliche Teilgruppen der Stichprobe) dargestellt. Zusammenhangs- und Unterschiedsanalysen finden sich bspw. bei Dreer et al. (2020), Huber et al. (2020), Porsch und Porsch (2020), Schreiner et al. (2020) sowie Wößmann et al. (2020). Multivariate Verfahren (bspw. Regressionsanalysen, Strukturgleichungsmodelle) auf Basis der Befragungsdaten wurden erst in wenigen Publikationen durchgeführt (Baier und Kamenowski 2020; Huber et al. 2020; Grätz und Lipps 2021; Huber und Helm 2020b; Klapproth et al. 2020; Lorenz et al. 2020b; Schreiner et al. 2020; Steinmayr et al. 2020; Zinn und Bayer 2020). Da inferenzstatistische Befunde die Ausnahme darstellen und um kohärent zu bleiben, beschränkt sich der vorliegende Review auf deskriptive Befunde.

### Dissemination

Bereits sehr früh wurden erste Publikationen in wissenschaftlichen Organen, z. B. in einschlägigen Verlagen wie Waxmann veröffentlicht. So erschien bereits am 24. April 2020 das Buch „COVID-19 – aktuelle Herausforderungen in Schule und Bildung“ (Huber et al. 2020). Am 10. Juni folgte eine englischsprachige Veröffentlichung „COVID-19 and schooling: evaluation, assessment and accountability in times of crises—reacting quickly to explore key issues for policy, practice and research with the school barometer“ (Huber und Helm 2020a) in der Educational Assessment, Evaluation and Accountability. International Journal of Policy, Practice and Research (SCI, Springer). Am 15. Juni 2020 erschien das Beiheft 16 „Langsam vermisse ich die Schule“ (Fickermann und Edelstein 2020) der Fachzeitschrift „Die Deutsche Schule“ (darin enthalten Huber und Helm 2020b; Porsch und Porsch 2020; Wacker et al. 2020). Kurz darauf, am 20. Juni 2020, erschien die Ausgabe „Nähe(n) und Distanz(en) in Zeiten der COVID-19-Krise“ im Journal „Medienimpulse“ (darin enthalten Tengler et al. 2020).

Die Mehrheit der Befragungen wurde allerdings über Kurzberichte auf den institutseigenen Webseiten veröffentlicht. Es ist zu erwarten, dass viele der hier vorgestellten Befragungen Basis von in naher Zukunft erscheinenden wissenschaftlichen Publikationen darstellen werden. So befanden sich zum Zeitpunkt der Manuskripterstellung mehrere Beiträge im Review (z. B. Grewenig et al. 2020; Holtgrewe et al. 2020b; Klapproth et al. 2020; Letzel et al. 2020; Steinmayr et al. 2020).

## Review 2: Beschreibung der Befragungen entlang des integrativen Rahmenmodells zum Fernunterricht

Die Analyse und Synthese der Befunde der existierenden 97 Befragungen zum Fernunterricht erfolgt entlang des integrativen Rahmenmodells zum Fernunterricht (siehe Abb. 1), das folgende Kategorien postuliert: Lernerfolg (Abschn. 5.1.1), Quantität des Schülerlernens (Abschn. 5.1.2), Qualität des Schülerlernens (in den Befragungen nicht erfasst, vgl. Abschn. 5.2 und 5.4.2), Lernmotivation (Abschn. 5.1.3), selbstgesteuertes Lernen (Abschn. 5.2), Merkmale der Schüler*innen (vgl. die Ausführungen zum Schultyp und sozioökonomischen Hintergrund der Schüler*innen im jeweiligen Abschnitt), häusliche Lernressourcen (Abschn. 5.3), Merkmale des Fernunterrichts (Abschn. 5.4), Merkmale der Lehrperson (Abschn. 5.5) und Technologieeinsatz für den Fernunterricht (Abschn. 5.6).

### Lernprozessmerkmale

#### Lernerfolg

Unserem Wissen nach liegt für den DACH-Raum bisher nur zwei Studien von Tomasik et al. (2020) und Depping et al. (2021) vor, die auf standardisierte Leistungstests zurückgreift, um die Lernentwicklung der Schüler*innen während den Schulschließungen zu analysieren. In den bisher veröffentlichten Befragungen wurde dagegen versucht, mittels Fremd- und Selbsteinschätzungen den Lernerfolg zu erfassen.

Aus Sicht der Schüler*innen variieren die Angaben zu Items, die vorsichtig als Indizien für den selbsteingeschätzten Lernerfolg interpretiert werden können, relativ stark. So berichten …

- 8 %, dass sie sich oft fragen, ob sie im Fernunterricht viel Stoff versäumen (Schreiner et al. 2020).
- etwa 20 %, dass sie zuhause schlechtere Leistungen erbringen als im Präsenzunterricht (Schwerzmann und Frenzel 2020).
- 26 %, dass sie besorgt sind schlechtere Noten zu bekommen. Weitere 21 % sind diesbezüglich zum Teil besorgt (Trültzsch-Wijnen und Trültzsch-Wijnen 2020).
- 35 %, dass sie sich oft Gedanken über ihr Abschlusszeugnis und ihre Noten machen (Schreiner et al. 2020).
- 38 %, dass sie (eher) nur sehr wenig lernen (Baier und Kamenowski 2020).
- 40 %, dass es nun schwieriger ist dem Curriculum zu folgen (Refle et al. 2020).
- 45 %, sich (sehr) große Sorgen über negative Auswirkungen auf ihre Schulleistungen zu machen (Anger et al. 2020).

Derartige Einschätzungen zu negativen Einflüssen des Fernunterrichts auf den Lernerfolg hängen allerdings stark vom Schulfach ab, wie die Befragung von Grütter et al. (2020) zeigt: Während für das Fach Deutsch 14 % der Schüler*innen meinen, die Lernziele (eher) nicht erreichen zu können, sind es für das Fach Mathematik 25 % der Schüler*innen. Diesen Schülergruppen, die von negativen Auswirkungen berichten, stehen Schülergruppen gegenüber, die positiveres berichten. Bei Huber et al. (2020) geben immerhin 24 % an, dass sie jetzt mehr als im normalen Unterricht lernen. Auch hier zeigen sich fachspezifische Unterschiede. Bei Heller und Zügel (2020) geben je nach Schulfach 36–53 % der Schüler*innen an, dass sie (durch die gestellten Aufgaben im Fernunterricht) viel lernen.

Die Einschätzungen der *Eltern* zum Lernerfolg ihrer Kinder während des Fernunterrichts decken sich mit den Befunden der Schülerbefragungen. Im Durchschnitt zeigen sich die Eltern während des Lockdowns eher neutral, weder besonders unzufrieden noch besonders zufrieden mit dem, was ihre Kinder im Fernunterricht gelernt haben, so die großangelegte Familienbefragung von Andresen et al. (2020). Werden Eltern dagegen nach dem Lernrückstand ihrer Kinder gefragt, sind es rund ein Drittel bis zwei Drittel, die negative Auswirkungen befürchten: 33 % der Eltern haben Sorge, dass ihre Kinder aufgrund der Schulschließung hinter dem eigentlichen Lernstand zurückbleiben (Huber et al. 2020), 34 % machen sich Sorgen darüber, ob ihr Kind auch genug lernt (Müller 2020), und über die Hälfte (56 %) der von Thies und Klein (2020) befragten Eltern befürchtet, dass ihre Kinder den Anschluss an den Schulstoff verlieren. Zudem meinen in der ifo-Elternbefragung (Wößmann et al. 2020) 64 %, dass ihre Kinder während der Schulschließung viel *weniger* gelernt haben.

Auch der Anteil der *Lehrkräfte,* die negative Auswirkungen auf den Lernerfolg ihrer Schüler*innen befürchten, liegt bei etwas mehr als einem Drittel. In den Befragungen von Steiner et al. (2020), forsa (2020c) und Schwerzmann und Frenzel (2020) gehen zudem zwischen 36 und 38 % der Lehrkräfte davon aus, dass ihre Schüler*innen den Jahresstoff nicht schaffen werden, Lernrückstände erleiden oder sich ihre Leistungen/Kompetenzen während der Schulschließung verschlechtern. Dies dürfte insbesondere damit zusammenhängen, dass die Lernstoffvermittlung während der Schulschließungen weniger effektiv ist als im regulären Unterricht, wie 77 % der von Eickelmann und Drossel (2020) befragten Lehrkräfte angeben. Etwas aus der Reihe fallen die hohen und widersprüchlichen Werte der beiden folgenden Lehrerbefragungen: Im Kanton Nidwalden berichten 87 % der Lehrpersonen, dass ihre Lernenden gute Lernfortschritte während des Fernunterrichts gemacht haben (Bildungsdirektion Nidwalden 2020). Demgegenüber stehen 79 % der von Lorenz et al. (2020b) befragten Lehrkräfte, die berichten, dass ihre Schüler*innen während der Schulschließung weniger als normalerweise gelernt haben.

##### Unterschiede nach dem sozioökonomischen Hintergrund

Sowohl Schülerbefragungen (Anger et al. 2020; Baier und Kamenowski 2020; Vuorikari et al. 2020) als auch Eltern- (Thies und Klein 2020; Wößmann et al. 2020) und Lehrerbefragungen (Steiner et al. 2020) berichten für Schüler*innen aus sozial- und bildungsbenachteiligten Familien stärkere Nachteile des Fernunterrichts bzgl. des Lernerfolgs der Schüler*innen als für andere Schüler*innen.

##### Unterschiede nach dem Schultyp

Sowohl Schülerbefragungen (Schwerzmann und Frenzel 2020; Trültzsch-Wijnen und Trültzsch-Wijnen 2020) als auch Eltern- (Müller 2020; Trültzsch-Wijnen und Trültzsch-Wijnen 2020) und Lehrerbefragungen (Bildungsdirektion Nidwalden 2020; forsa 2020c; Eickelmann und Drossel 2020; Steiner et al. 2020) berichten bzgl. der Nachteile des Fernunterrichts hinsichtlich des Lernerfolgs der Schüler*innen *keine* wesentlichen Unterschiede zwischen den Schultypen.

#### Aufgewandte Lernzeit

**Figure 4:**
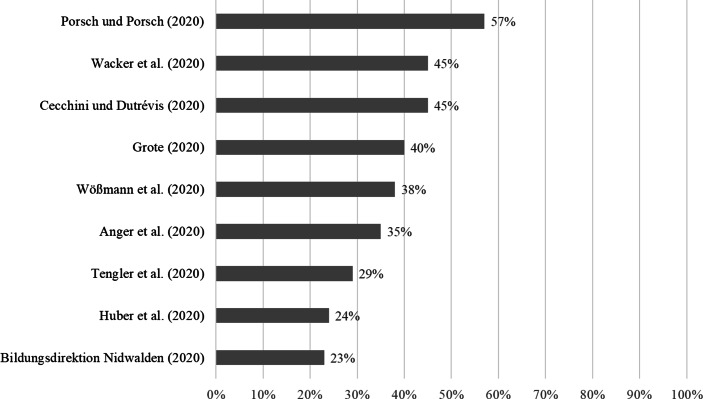

Laut der JIM plus-Befragung (Rathgeb 2020) geben 25 % der Schüler*innen an, täglich zu lernen. Weitere Befragungen – siehe Abb. 4 –, berichten ebenfalls über ein relativ niedriges tägliches Lernengagement der Schüler*innen an typischen Schultagen.^10^ Allerdings zeigt Abb. 4 auch, dass die Befunde zur Frage, wie viele Stunden Schüler*innen während der Schulschließungen täglich für die Schule aufwenden, keineswegs einheitlich sind, sondern stark zwischen den Befragungen variieren. Mögliche Gründe dafür können in der je nach Befragung unterschiedlichen Stichprobenzusammensetzung, Operationalisierung des Lernaufwands sowie des unterschiedlichen Befragungszeitpunktes liegen. Hier deutet sich weiterer Forschungsbedarf an.

In den* Schüler*befragungen von Huber et al. (2020), Anger et al. (2020), Cecchini und Dutrévis (2020) und Wacker et al. (2020) geben 24 %, 35 %, 45 % und 45 % der befragten Schüler*innen an, dass sie maximal 2 h pro Tag für Schulaktivitäten aufwenden.^11^ In vier Befragungen wird der durchschnittliche Lernaufwand pro Tag berichtet. Auch hier variieren die Durchschnittswerte deutlich: So werden bei Schober et al. (2020a) 5 h pro Tag berichtet, während es bei Huber et al. (2020; vgl. Huber und Helm 2020b) nur 2,7 h pro Tag sind. Dazwischen liegen die Befragung von Letzel et al. (2020) und jene von Holtgrewe et al. (2020a), die von knapp 4^12^ bzw. 3,5 h durchschnittlichem Lernaufwand pro Tag berichten. Verglichen mit der nominellen Lernzeit vor der Schulschließung mag dies wenig erscheinen. So berichten Refle et al. (2020; siehe auch Grätz und Lipps 2021) für Schweizer Lernende im Alter von 14–26 Jahren eine durchschnittliche Lernzeit von 36 h pro Woche vor den Schulschließungen. Während des Fernunterrichts sank diese Zahl um 12 h auf 23 h^13^. Im Vergleich dazu gibt die Mehrheit der *Mitarbeitenden der Schule und Schulleitungen* in der Befragung von Huber et al. (2020) an, dass Schüler*innen elf bis 13 h Bearbeitungszeit pro Woche für Lernaufgaben aufwenden müssen. Schließlich finden sich Befragungen, die nach globalen Schülereinschätzungen zum Lernaufwand während des Fernunterrichts fragen. In der internationalen Schülerbefragung der Europäischen Kommission (Vuorikari et al. 2020) geben 33 %, 29 % und 43 % an, dass ihr Workload während des Fernunterrichts geringer als vor dem Lockdown war. In der deutschen „JIMplus Corona“-Studie (Rathgeb 2020) sind dies 45 % der befragten Jugendlichen.

In den *Eltern*befragungen von Tengler et al. (2020), Wößmann et al. (2020), Grote (2020) und Porsch und Porsch (2020) geben 29 %, 38 %, 40 % und 57 % der Eltern an, dass die für schulische Aktivitäten aufgewendete Zeit täglich 2 h und weniger beträgt. Demgegenüber geben in der Elternbefragung von Langmeyer et al. (2020) rund 10 % der Eltern an, dass sich die Häufigkeit der Freizeitaktivität „zuhause etwas für die Schule tun“ im Vergleich zu vor dem Fernunterricht reduziert hat.

##### Unterschiede nach dem sozioökonomischen Hintergrund

Eine Schülerbefragung (Anger et al. 2020) und eine Elternbefragung (Wößmann et al. 2020) berichten *keine* Unterschiede für die investierte Lernzeit von Schüler*innen aus sozial- und bildungsbenachteiligten Familien. Einzig bei Grätz und Lipps (2021) wird widererwarten ein stärkerer Rückgang der Lernzeit für Schüler*innen aus sozioökonomisch besser gestellten Familien beobachtet.

##### Unterschiede nach dem Schultyp

Hier ist die Befundlage nicht eindeutig. Sowohl aus Schülerbefragungen als auch aus Elternbefragungen liegen Befunde vor, die *keine* systematischen und *keine* wesentlichen Unterschiede in der investierten Lernzeit von Schüler*innen unterschiedlicher Schultypen berichten (Grätz und Lipps 2021; Heller und Zügel 2020; Langmeyer et al. 2020; Refle et al. 2020; Trültzsch-Wijnen und Trültzsch-Wijnen 2020). Demgegenüber stehen Schüler- und Elternbefragungen, die zeigen, dass die investierte Lernzeit mit der Schulstufe und dem höheren Schultyp ansteigt (Bildungsdirektion Nidwalden 2020; Holtgrewe et al. 2020a; Thies und Klein 2020; Wößmann et al. 2020). Interessant ist darüber hinaus auch, dass mehrere Befragungen (Holtgrewe et al. 2020a; Müller 2020; Refle et al. 2020) zeigen, dass die Reduktion der Lernzeit aufgrund des Fernunterrichts für jüngere Schüler*innen höher ausfällt als für ältere Schüler*innen.

#### Lernmotivation

Aus *Schülersicht* geben je nach Befragung 37–70 % der Schüler*innen an, (eher) gerne oder sehr gerne im Fernunterricht zu lernen (37 % bei Huber et al. 2020; 46 % bei Trültzsch-Wijnen und Trültzsch-Wijnen 2020; 55 % bei Holtgrewe et al. 2020a; 55 % bei Baier und Kamenowski 2020; 48–70 % bei Schwerzmann und Frenzel 2020).

In der *Elternbefragung* von Wildemann und Hosenfeld (2020) ist der Anteil der Eltern, die ihr Kind als motiviert einschätzen (49 %) annährend gleich groß wie jener, die ihre Kinder als nicht motiviert einschätzen (51 %). In den großangelegten Befragungen der Elternverbände in Sachsen und Hamburg führen 41 % und 38 % der Eltern an, dass die fehlende Motivation ihrer Kinder eine echte Herausforderung darstellt (Landeselternrat Sachsen 2020) bzw. es ihren Kindern schwer fällt sich zu motivieren (Müller 2020). Einen ähnlichen Wert berichten Tengler et al. (2020): 41 % der Eltern führen die fehlende Lernmotivation als Hindernis für den Fernunterricht an. Demgegenüber steht der Befund von Huber et al. (2020): Nur 27 % der Eltern sind der Meinung, dass sich ihre Kinder (eher) nicht auf andere Lernmethoden wie z. B. E‑Learning freuen. Befunde von Heller und Zügel (2020) bestätigen dies. Sie zeigen, dass Eltern das Arbeitsverhalten – darunter auch die Motivation – ihre Kinder tendenziell als gut bewerten.

Im Vergleich zu den Eltern- und Schülereinschätzungen der Schülermotivation ist jene der *Lehrkräfte* deutlich pessimistischer. Bei Klapproth et al. (2020), Seda und Ottacher (2020) sowie Steiner et al. (2020) bezeichnen über 60–70 % der Lehrkräfte die fehlende Lernmotivation der Schüler*innen als Herausforderung für den Fernunterricht. Bei Tengler et al. (2020) und Schrammel et al. (2020) sind es mit 40 % der Lehrkräfte dagegen deutlich weniger. Auch andere Indikatoren der Schülermotivation aus Lehrersicht stimmen etwas optimistischer: Dreer et al. (2020) berichten, dass „nur“ 40 % der Lehrkräfte denken, dass ihre Schüler*innen den Fernunterricht *nicht* gut finden. 26 % der von Huber et al. (2020) befragten Mitarbeitenden der Schule denken, dass die Kinder sich *nicht* auf andere Lernmethoden freuen.

##### Unterschiede nach dem sozioökonomischen Hintergrund

Zwei Schülerbefragungen (Baier und Kamenowski 2020; Heller und Zügel 2020) verweisen darauf, dass Schüler*innen aus sozioökonomisch schlechter gestellten Familien den Fernunterricht *weniger* positiv erleben.

##### Unterschiede nach dem Schultyp

Während die Schülerbefragung von Schwerzmann und Frenzel (2020) zeigt, dass mit Anstieg des Schultyps auch die Schülermotivation im Fernunterricht ansteigt, können in der Schülerbefragung von Heller und Zügel (2020) diesbezüglich *keine* systematischen Unterschiede beobachtet werden. In mehreren Befragungen (Baier und Kamenowski 2020; Müller 2020; Schwerzmann und Frenzel 2020) zeigt sich zudem, dass die Lernmotivation im Fernunterricht bei Gymnasialschüler*innen im Vergleich zu Schüler*innen anderer Schultypen niedriger ausgeprägt ist.

### Schülermerkmale

#### Selbstständigkeit aus Schülersicht

Während der Schulschließung waren viele Schüler*innen auf sich gestellt. Sowohl das Lernen als auch die gesamte Tagesstruktur lag je nach familiärer Unterstützung mehr oder weniger stark in der Verantwortung der Schüler*innen.

Bezüglich der *Organisation des Tagesablaufs* gaben in der Befragung von Huber et al. (2020) (nur) 19 % der befragten Schüler*innen an, dass die eigenständige Tagesplanung eine Herausforderung darstellt. Zudem gaben 37 % der Schüler*innen an, dass es ihnen *nicht* leichtfällt, früh aufzustehen und einem geregelten Tagesablauf nachzugehen. Bei Schreiner et al. (2020) gaben 16 % der Schüler*innen an, dass es ihnen oft schwer fiel den Tag zu strukturieren. Die Rolle der Routine des Unterrichts für die Schüler*innen wurde in der Befragung von Refle et al. (2020) erhoben: 50 % vermissen die Routine, die der Unterricht bietet; weitere 22 % vermissen sie teilweise.

Bezüglich des *selbstgesteuerten Lernens* bestätigt bspw. die Befragung von Schwerzmann und Frenzel (2020) die höhere Eigenverantwortung: Je nach Schultyp geben 42 % (Sonderschule) bis 75 % (Gymnasium) der Schüler*innen an, im Fernunterricht selbstständiger als im Präsenzunterricht zu arbeiten. Dies ist allerdings für nicht wenige Schüler*innen mit Schwierigkeiten verbunden. In der Befragung von Letzel et al. (2020) geben 35 % der Schüler*innen an, dass sie mit dem Fernunterricht *nicht* gut zurechtkommen. 11 % sind es bei Schreiner et al. (2020), die berichten, dass für sie das Lernen zuhause schwierig ist. Bei Schwerzmann und Frenzel (2020) berichten 36 % der Schüler*innen von Konzentrationsproblemen im Fernunterricht. In der Wiener Befragung von Holtgrewe et al. (2020a) geben 35 % an, dass sie sich verunsichert und überfordert fühlen. Auch geben 22 % an, dass sie inhaltliche Schwierigkeiten bei der Bewältigung der Aufgaben haben. Ein ähnlich hoher Prozentsatz ist es in der Berliner Befragung der Anlaufstelle für Diskriminierungsschutz an Schulen (ADAS und LIFE 2020): 26 % der befragten Schüler*innen berichten Probleme damit gehabt zu haben, die Aufgaben im Fernunterricht eigenständig zu bearbeiten. Interessante Hinweise zum Einsatz von *Lernstrategien* der Schüler*innen im Fernunterricht finden sich bei Schober et al. (2020a) und Holtgrewe et al. (2020a). Erstere Befragung berichtet, dass die Mehrheit der Schüler*innen (70 %) sich beim Lernen einen Plan für die zu erledigenden Aufgaben macht. Jedoch halten nur 38 % der befragten Schüler*innen täglich fixe Lernzeiten ein. In der zweiten Befragungswelle am Ende der Schulschließungen geben immerhin 30 % der befragten Schüler*innen an, dass ihnen die Organisation des Lernens im Vergleich zu Beginn des Fernunterrichts deutlich besser gelingt; 36 % berichten, dass die Aufgabenbearbeitung im Vergleich zum Beginn des Fernunterrichts nun (eher) besser gelingen würde. In der zweiten Befragung (Holtgrewe et al. 2020a) finden sich Hinweise, dass Schüler*innen mit einfacher qualifizierten Eltern häufiger (84 %) versuchen, mit Lernschwierigkeiten selbstständig zurechtzukommen als Schüler*innen mit hochqualifizierten Eltern (46 %). Auch suchen sie häufiger Hilfe bei Lehrer*innen oder Mitschüler*innen, während Schüler*innen von hochqualifizierten Eltern häufiger Eltern und Geschwister zurate ziehen.

Neben Fähigkeiten des selbstgesteuerten Lernens verlangt der Fernunterricht den Schüler*innen auch Fähigkeiten im *Umgang mit digitalen Medien* ab. Vor diesem Hintergrund haben Trültzsch-Wijnen und Trültzsch-Wijnen (2020) in ihrer österreichischen Schülerbefragung Skalen zur Erfassung der selbsteingeschätzten Digital Skills der Lernenden eingesetzt. Zentrale digitale Skills wie die Fähigkeit, den Wahrheitsgehalt von Onlineinformationen zu prüfen oder Informationen über das Internet zu teilen, werden laut Selbstauskünften von 42–66 % der Schüler*innen beherrscht.^14^ Auch schätzen 46–65 % der Schüler*innen ihre Selbstwirksamkeit in Bezug auf verschiedene Online-Lernaktivitäten als gut ein. Auch Holtgrewe et al. (2020a) haben österreichische Schüler*innen (ab 11 Jahren) zu ihren Fähigkeiten im Umgang mit digitalen Medien befragt. 87 %, 80 % und 63 % der Schüler*innen berichten keine Schwierigkeiten mit dem Computer, Online-Meeting-Tools und Lernplattformen zu haben. In der Tiroler Schülerbefragung von Schreiner et al. (2020) berichten 56 %, dass sie selten oder nie Mühe im Umgang mit digitalen Medien hatten. Für alle drei DACH-Länder zeigen Vuorikari et al. (2020), dass „nur“ 15 % (Deutschland), 12 % (Österreich) und 13 % (Schweiz) der Schüler*innen angeben, dass sie *nicht* schnell lernen, wie man an Online-Lernaktivitäten teilnimmt. Aus *Lehrersicht* berichten Eickelmann und Drossel (2020) über alle Schultypen hinweg, dass das fehlende Know-how der Schüler*innen für 53 % der Lehrpersonen eine Herausforderung für den Fernunterricht darstellt.

#### Selbstständigkeit aus Elternsicht

Aus Sicht der *Eltern* scheinen die selbstregulativen Fähigkeiten ihrer Kinder für das *selbstgesteuerte Lernen *und für die* eigenständige Organisation des Tagesablaufes* gut ausgeprägt zu sein. So berichten Huber et al. (2020), dass „nur“ 30 % der Eltern denken, dass es ihren Kindern (eher) nicht leichtfällt, früh aufzustehen und einem geregelten Tagesablauf nachzugehen. Bzgl. des selbstständigen Lernens geben 80 % der von der Bildungsdirektion Nidwalden (2020) befragten Eltern an, dass ihre Kinder im Fernunterricht Aufgaben selbstständig bearbeiten konnten. Auch schätzen bei Heller und Zügel (2020) rund die Hälfte der Eltern (46–55 %) das Arbeitsverhalten ihrer Kinder (Selbstständigkeit, Konzentration, Durchhaltevermögen) hoch bis sehr hoch ein. Bei Porsch und Porsch (2020) und Müller (2020) geben zudem 45–60 % an, dass ihre Kinder die Aufgaben selbstständig und konsequent bearbeiten. „Nur“ 26 % der von Müller (2020) befragten Eltern geben an, dass sich ihr Kind überfordert fühlt. Auch bei Tengler et al. (2020) sind es „nur“ 22 % und 27 % der Eltern, die die Überforderung und das mangelnde Zeitmanagement ihrer Kinder als Herausforderung für erfolgreichen Fernunterricht sehen. Demgegenüber steht die Studie von ADAS und LIFE (2020), in der von 61 % der Eltern berichtet wird, die angeben, dass ihre Kinder die Aufgaben nur schlecht oder sehr schlecht eigenständig bearbeiten können. Schließlich geben auch bei Conus und Durler (2020) nur 4 % der Eltern an, dass ihre Kinder bei den schulischen Aufgaben nie Hilfe brauchten.

Vuorikari et al. (2020) befragten die Eltern zu einer Reihe von *Online und Distance Learning Skills* ihrer Kinder (z. B. Mein Kind ist besser im Organisieren von Lernaufgaben geworden). Konkret wurde erfragt, ob sich diese Skills während des Lockdowns verbessert haben. Je nach Skill meinen 35–51 % der Eltern aus Österreich, 45–58 % der Eltern aus Deutschland und 37–52 % der Eltern aus der Schweiz, dass sich die entsprechenden Skills ihrer Kinder während des Lockdowns verbessert haben. Die niedrigste Zustimmung erlangte in jedem Land das Item „Mein Kind beschäftigte sich stärker mit schulischen Aktivitäten“; die höchste Zustimmung erhielt jeweils das Item „Mein Kind hat mehr Autonomie gewonnen, z. B. durch den Einsatz digitaler Technologien“.

#### Selbstständigkeit aus Lehrersicht

Die Befunde aus den *Lehrerbefragungen* lassen sich in die drei Bereiche „selbstständiges Arbeiten“, „Schülerfähigkeiten als Herausforderungen für den Fernunterricht“ und „Medienkompetenzen“ clustern.

##### Selbstständiges Arbeiten

97 % der im Kanton Nidwalden befragten Lehrkräfte (Bildungsdirektion Nidwalden 2020) geben an, dass ihre Schüler*innen zuverlässig an ihren Aufgaben arbeiten. Im Kanton Waadt sind es je nach Schultyp zwischen 63 % (Zyklus 1) und 71 % (Zyklus 2) der Lehrkräfte, die ihre Schüler*innen als selbstständig bezeichnen (LEARN EPFL 2020). Auch Schwerzmann und Frenzel (2020) berichten Schultypenunterschiede: Zwischen 25 % (Sonderschule) und 64 % (Gymnasium) der Lehrpersonen stimmen der Aussage zu, dass ihre Schüler*innen im Fernunterricht selbstständiger arbeiten als im Präsenzunterricht. Auch bei Schwab et al. (2020) meinen 80 % der befragten Lehrpersonen, dass es ihren Schüler*innen gut gelungen ist, sich auf neue Lernmethoden einzulassen.

##### Herausforderungen für erfolgreichen Fernunterricht

Als Herausforderungen im Bereich der Schülerfähigkeiten zum selbstständigen Lernen nennen die Lehrkräfte die Tagesstruktur und Selbstorganisation (90 %, Steiner et al. 2020; 50 % Seda und Ottacher 2020), die Ablenkung beim Lernen zuhause (71 %, Steiner et al. 2020), das Zeitmanagement (55 %, Schrammel et al. 2020; Tengler et al. 2020), die Überforderung allgemein (33 %, Steiner et al. 2020) und mit dem E‑Learning (55 %, Seda und Ottacher 2020), das fehlende Know-how im Umgang mit den Lernangeboten (53 %, Eickelmann und Drossel 2020), die Selbstständigkeit (42 %, Schrammel et al. 2020; Tengler et al. 2020) und Konzentrationsprobleme (8 %, Schwerzmann und Frenzel 2020).

##### Medienkompetenz

In Bezug auf eine ausreichende Medienkompetenz der Schüler*innen zeichnen die von Lorenz et al. (2020b) befragten Lehrkräfte ein eher kritisches Bild, wobei in Grundschulen die Medienkompetenz der Schüler*innen wie erwartet geringer eingeschätzt wird als an Gymnasien.

##### Unterschiede nach dem sozioökonomischen Hintergrund

Sowohl Schülerbefragungen (Holtgrewe et al. 2020a; Vuorikari et al. 2020)^15^ als auch Eltern- (Conus und Durler 2020) und Lehrerbefragungen (Steiner et al. 2020) berichten von einem positiven Zusammenhang zwischen dem sozioökonomischen Hintergrund der Schüler*innen und den Fähigkeiten für das selbstgesteuerte Lernen im Fernunterricht.

##### Unterschiede nach dem Schultyp

Sowohl Schülerbefragungen (Schwerzmann und Frenzel 2020; Bildungsdirektion Nidwalden 2020; ADAS und LIFE 2020; Heller und Zügel 2020) als auch Eltern- (Müller 2020) und Lehrerbefragungen (Schwerzmann und Frenzel 2020) berichten erwartungsgemäß von einem deutlichen Anstieg der Schülerfähigkeiten zum selbstgesteuerten Lernen mit der Schulstufe bzw. mit dem höheren Schultyp. Dreer et al. (2020) zeigen allerdings, dass die Lehrereinschätzung der Eigenständigkeit des Lernens der Schüler*innen an der eigenen Schule nicht dieser Systematik folgt.

### Merkmale der häuslichen Ressourcen für das Lernen zuhause

#### Technische Ausstattung

**Figure 5:**
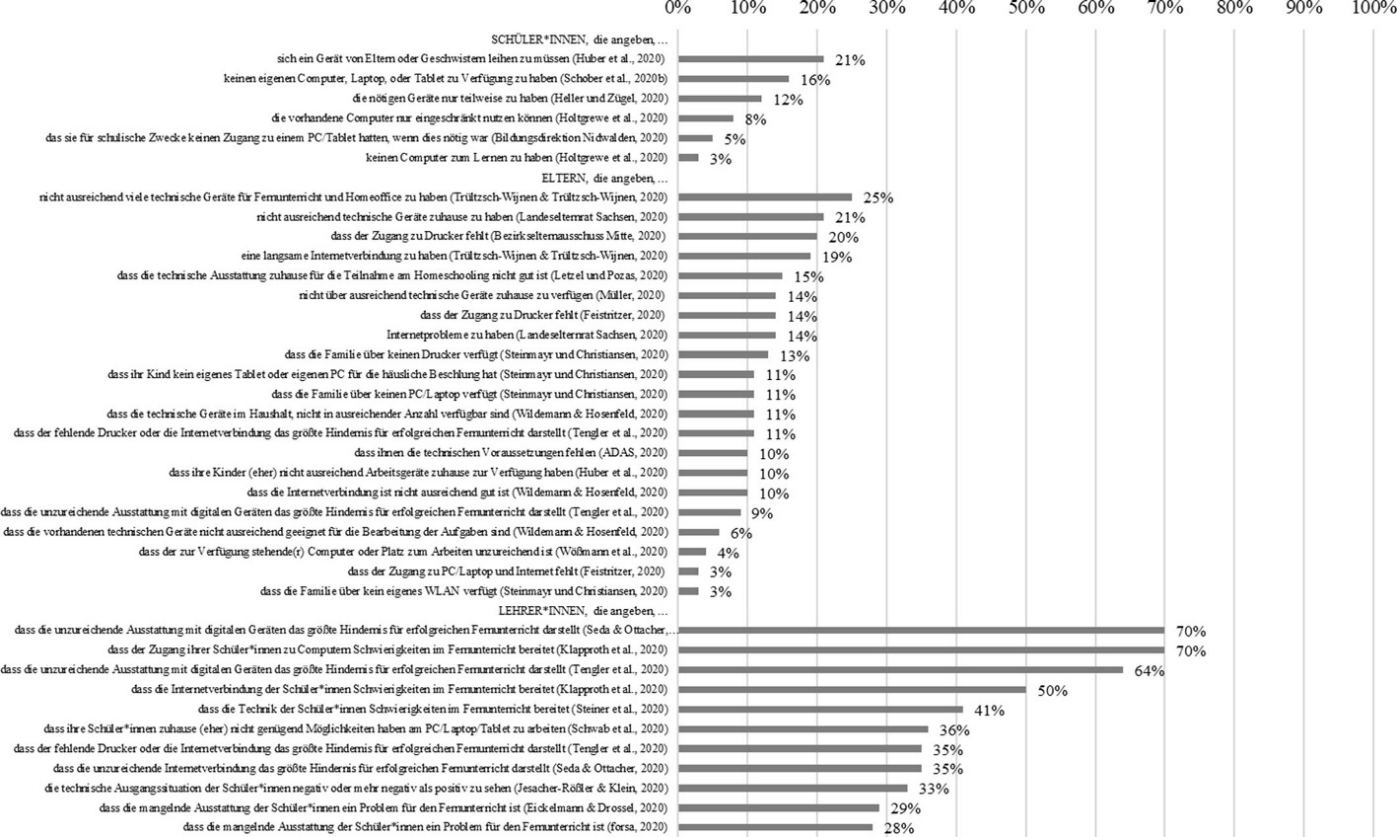

Abb. 5 zeigt, dass aus Sicht der Schüler*innen und Eltern zwischen 3 und 21 % bzw. 25 % der Befragten angeben, dass die für den Fernunterricht nötige und zuhause verfügbare technische Ausstattung „mangelhaft“ ist. So steht bspw. kein eigener Computer, Laptop, oder kein eigenes Tablet zur Verfügung, sodass diese Geräte von Eltern oder Geschwistern geliehen werden müssen. Besonders kritisch erwiesen sich in der Zeit der Schulschließungen der fehlende Zugang zu einem Drucker und eine mangelhafte Internetverbindung. Aus Sicht der Lehrkräfte stellt sich die Situation deutlich prekärer dar. Je nach Befragung sehen 28–70 % der Lehrkräfte in der technischen Ausstattung der Schüler*innen eine Herausforderung für den Fernunterricht.

Ähnlich wie bei der technischen Ausstattung scheinen die häuslichen Arbeitsplatzbedingungen der Schüler*innen nur für einen kleineren Teil eine Herausforderung darzustellen. Je nach Schultyp berichten bei Schwerzmann und Frenzel (2020) 88–96 % der Schüler*innen über einen ausreichend großen Arbeitsplatz zu verfügen. Über einen ruhigen Arbeitsplatz zuhause berichten ebenfalls 85–90 %. Nur 6 % der Schüler*innen geben an, dass sie aufgrund der Wohnsituation zuhause nicht wirklich lernen können (Baier und Kamenowski 2020). Auch aus Elternsicht berichten nur 4 % der *Eltern*, dass ein geeigneter Raum fehlt, um ihr Kind bei den Lernaufgaben zu unterstützen (Lochner 2020). In der Elternbefragung von Langmeyer et al. (2020) geben sogar 78 % der Eltern an, für jedes Kind ein eigenes Kinderzimmer zu haben. Aus Sicht der *Lehrkräfte* wird der fehlende ruhige Arbeitsplatz aber deutlich häufiger (39 %) als Problem für den Fernunterricht angeführt (Steiner et al. 2020). Auch wird die allgemeine Wohnsituation der Schüler*innen von Lehrkräften häufig als Problem für den Fernunterricht angeführt (35 % bei Seda und Ottacher 2020; 50 % bei Klapproth et al. 2020).

##### Unterschiede nach dem sozioökonomischen Hintergrund

In den Befragungen ist wiederholt zu beobachten, dass die Einschätzung der Qualität der technischen Ausstattung der Schüler*innen mit dem sozioökonomischen Status der Schüler*innen steigt (ADAS und LIFE 2020; Holtgrewe et al. 2020a; Wößmann et al. 2020).

##### Unterschiede nach dem Schultyp

Während in der Luzerner Schülerbefragung (Schwerzmann und Frenzel 2020) Schüler*innen des Zyklus 1 deutlich seltener als Schüler*innen höherer Schultypen davon berichten, über verschiedene technische Mittel (insbesondere Drucker, Computer/Laptop, Handy) zu verfügen, können in der Nidwaldener Schülerbefragung (Bildungsdirektion Nidwalden 2020) keine Schultypenunterschiede ausgemacht werden. Auch die Waadter Lehrerbefragung (LEARN EPFL 2020) und die Berliner Elternbefragung (Bezirkselternausschuss Mitte 2020) verweisen nur auf kleine Schultypenunterschiede in der technischen Ausstattung der Schüler*innen für den Fernunterricht.

#### Familiäre Unterstützung

##### Rolle der Mütter

Gerade in der Krise zeigt sich, wie sehr traditionelle Geschlechterrollen in unserer Gesellschaft immer noch verankert sind. Die Mehrfachbelastung durch die Schulschließungen trifft nahezu ausschließlich Mütter. In den Befragungen berichten 81 % der Mütter (Wildemann und Hosenfeld 2020) und 59 % bzw. 84 % der Schüler*innen (Schober et al. 2020a; Heller und Zügel 2020), dass die elterliche Lernunterstützung in erster Linie von Müttern geleistet wird bzw. sich diese dafür hauptverantwortlich sehen (vgl. auch ähnliche Befunde bei ADAS und LIFE 2020 und Langmeyer et al. 2020).

##### Ausmaß der Lernunterstützung durch die Eltern

Durch die Schulschließung ist eine höhere Unterstützung der Schüler*innen beim Lernen durch die Eltern gefordert. Nicht immer können Eltern dieser Forderung nachkommen. Über alle Schülergruppen hinweg berichten 21 % (Schober et al. 2020a) bzw. 33 % der Schüler*innen (Refle et al. 2020), dass sie zuhause keine oder nicht die notwendige Unterstützung beim Lernen erhalten.^16^ Diese Anteilswerte können jedoch Spitzen verdecken: So geben bei Holtgrewe et al. (2020) 67 % der Mädchen und 50 % der Jungen über 14 Jahre an, dass sich ihre Eltern keine Zeit nehmen (können), um mit ihnen zu lernen. Demgegenüber stehen Befunde, wonach sich nur ein kleinerer Teil der Schüler*innen (10 %) mehr Elternunterstützung wünscht (Letzel et al. 2020). Aus *Sicht der Eltern* wird die Notwendigkeit der Lernbetreuung bestätigt. 50 % der Eltern in der Hamburger *Elternbefragung* (Müller 2020) geben an, dass sie ihr Kind mehr als normalerweise unterstützen müssen (vgl. auch Ziegler und Hannemann 2020). Bei Eltern von Schüler*innen in der Grundschule sind dies sogar 62 % (Müller 2020). Allerdings nimmt der *Unterstützungsbedarf* mit dem Alter der Schüler*innen ab (Holtgrewe et al. 2020a; Müller 2020; Rathgeb 2020). Diesem Unterstützungsbedarf können Eltern auch laut eigenen Angaben nicht immer gerecht werden. Als Grund beklagen sie häufig (je nach Befragung 25–66 %) die *fehlende Zeit *für die Lernunterstützung (25 % bei Trültzsch-Wijnen und Trültzsch-Wijnen 2020; 43 % bei Thies und Klein 2020; 55 % bei Letzel et al. 2020; 59 % bei ADAS und LIFE 2020; rund 60 % bei Landeselternrat Sachsen 2020; 66 % bei Lochner 2020; vgl. auch Ziegler und Hannemann 2020)^17^. Aus *Lehrersicht* meinen 39 % der von Steiner et al. (2020) befragten Lehrkräfte, dass die fehlende Elternunterstützung ein Problem für den Fernunterricht darstellt (Steiner et al. 2020). In mehreren Befragungen wurden die Eltern direkt nach der *aufgewandten Zeit* für die Lernunterstützung im Fernunterricht gefragt. Auch hier zeigt sich der von den Eltern monierte Zeitmangel: Je nach Befragung geben 24–63 % der Eltern an, durchschnittlich weniger als 1 h pro Tag für die Lernunterstützung ihrer Kinder Zeit zu haben (24 %^18^ bei Wildemann und Hosenfeld 2020; 35 % bei Grote 2020; 53 % bei Cordes 2020; 63 %^19^ bei Heller und Zügel 2020). Zudem berichten Thies und Klein (2020), Berghammer (2020) und Wößmann et al. (2020) von durchschnittlich rund 3 h, 2 h und 1 h pro Tag, die Eltern für die Lernbetreuung ihrer Kinder aufwenden. Als zentrale Gründe für die mangelnde Elternunterstützung werden in der sächsischen Elternbefragung (Landeselternrat Sachsen 2020; vgl. auch Tengler et al. 2020) die Berufstätigkeit der Eltern, die Anzahl der zuhause zu betreuenden Kinder und das fehlende nötige Fachwissen genannt.

##### Kompetenzen und Selbstwirksamkeit der Eltern

In der Befragung von Huber et al. (2020) und Holtgrewe et al. (2020a) sehen 14 % bzw. 37 % der Schüler*innen eine Herausforderung der Schulschließung darin, dass ihre Eltern ihnen nicht helfen können. Aus Sicht der Eltern berichten 34 % (Feistritzer et al. 2020), dass es für sie schwierig ist, ihren Kindern bei den Aufgaben zu helfen. Möglich Gründe dafür liegen neben der aufgrund der Mehrfachbelastung der Eltern fehlenden Zeit im *fehlenden Fachwissen* der Eltern. In mehreren Elternbefragungen geben 15–35 % der befragten Eltern an (15 % bei Trültzsch-Wijnen und Trültzsch-Wijnen 2020; 16 % bei Wildemann und Hosenfeld 2020; 19 % bei Lochner 2020; 25 % bei Grote 2020; 27 % bei Landeselternrat Sachsen 2020; 27 % bei Letzel et al. 2020; 34 % bei Feistritzer et al. 2020; 35 % bei Thies und Klein 2020), dass ihnen das nötige *Fachwissen* für die Lernunterstützung ihrer Kinder fehle bzw. sie die inhaltliche Betreuung (z. B. Überprüfung des Wissens der Lernenden vor Tests) nicht gewährleisten könnten. Bei Trültzsch-Wijnen und Trültzsch-Wijnen (2020) meinen zudem 13 % der in Österreich befragten Eltern, dass ihnen die nötigen *digitalen Kompetenzen* fehlen. Die Beherrschung konkreter digitaler Skills (z. B. Fähigkeit zur Prüfung des Wahrheitsgehalts von Online-Informationen) verneinen zwischen 4 und 13 % der Eltern. Für alle drei DACH-Länder berichten Vuorikari et al. (2020) von folgenden Anteilswerten für Eltern ohne oder mit geringen digitalen Kompetenzen: 19 % (DE), 24 % (AT) und 17 % (CH).

Drei Befragungen haben nach der *Selbstwirksamkeitseinschätzung* der Eltern in ihrer Rolle als Lehrperson zuhause gefragt. Die Befunde bei Porsch und Porsch (2020) verweisen auf relativ hohe Werte: Eltern erleben sich in den Fächern Deutsch, Mathematik und Sachunterricht als selbstwirksam und enthusiastisch. Dieser Befund deckt sich mit jenem von Wildemann und Hosenfeld (2020): Die deutliche Mehrheit (84 %) der befragten Eltern empfindet die Homeschooling-Aufgaben im Verhältnis zu den Kompetenzen der Eltern als nicht schwer. Auch bei Thies und Klein (2020) und bei Wößmann et al. (2020) gibt ein ähnlich hoher Anteil (71 % bzw. 86 %) der Eltern an, mit der Organisation des Unterrichts zuhause bzw. mit der Situation allgemein gut zurecht zu kommen. Dieser selbstwirksamen Elterngruppe steht eine ebenfalls nicht kleine Gruppe von Eltern gegenüber, die sich mit der Beschulung (29 % bei Müller 2020) bzw. der Betreuung (46 % bei Kugelmeier und Schmolze-Krahn 2020; 13 % bei Zinn und Bayer 2020) zuhause überfordert fühlen, diese als echte Herausforderung empfinden (15 % bei Huber et al. 2020) oder sich nicht gut in der Lage fühlen, ihre Kinder bei der Bearbeitung der Lernaufgaben zu unterstützen (15 % bei Lochner 2020). Auch sagen 14 % der von Trültzsch-Wijnen und Trültzsch-Wijnen (2020) befragten Eltern, sie seien nicht fähig, ihre Kinder bei Motivationsproblemen zum Lernen zu bringen und 3 % finden es schwer, bei den Schulaufgaben ihrer Kinder dranzubleiben. Auch die *Fremdeinschätzung aus Sicht der Lehrkräfte* zeichnet ein eher pessimistisches Bild: Die Lehrerbefragung von Steiner et al. (2020) kommt zu dem Schluss, dass 61 % der Lehrkräfte denken, dass die Eltern mit Homeschooling überfordert sind. Darüber hinaus sehen 37 % der Lehrkräfte in der Befragung von Tengler et al. (2020) die Motivation der Eltern als ein großes Hindernis für erfolgreichen Fernunterricht.

##### Gestaltung der elterlichen Lernunterstützung

**Figure 6:**
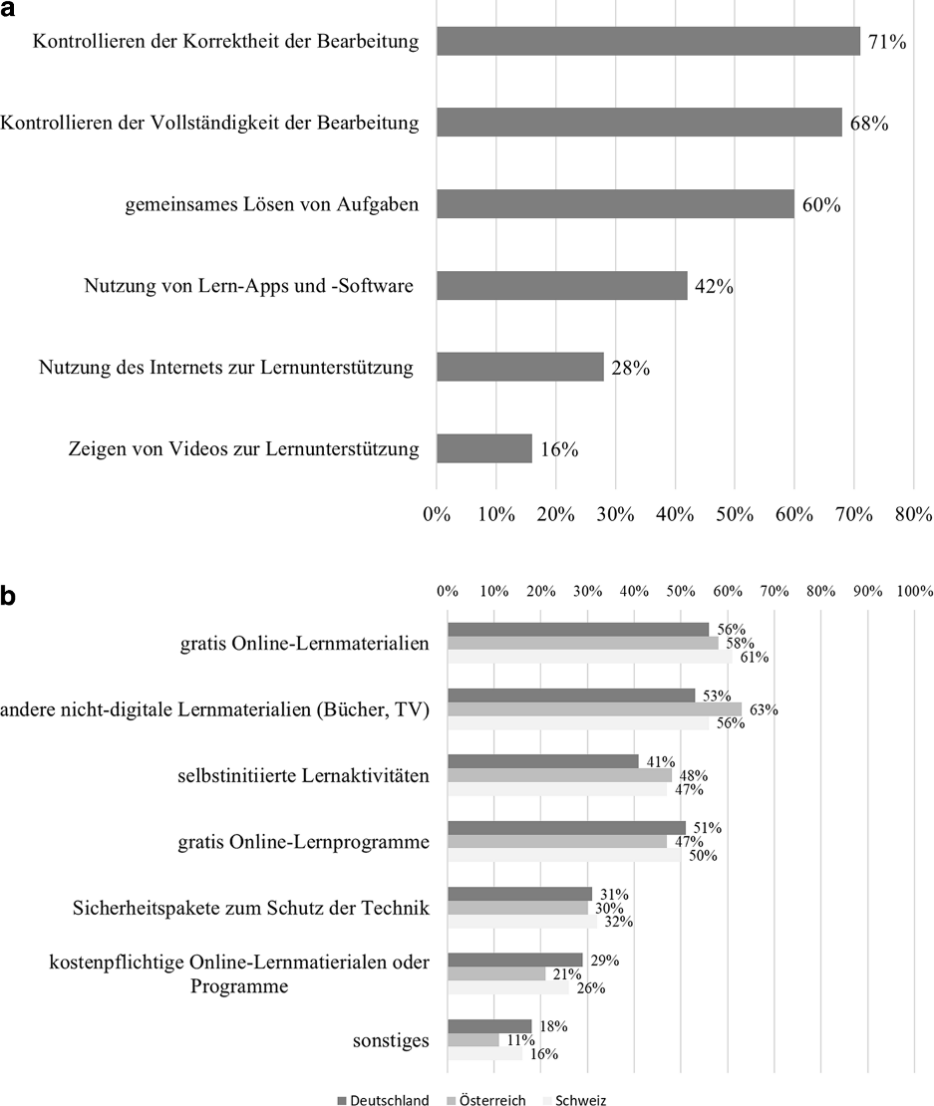

Wie sieht die Lernunterstützung der Eltern konkret aus? Die Befunde von Porsch und Porsch (2020; vgl. Abb. 6a) zeigen, dass die elterliche Lernunterstützung in den häufigsten Fällen (zwischen 68 und 71 %) darin besteht, die Korrektheit und Vollständigkeit der Bearbeitung der Lernaufgaben durch die Schüler*innen zu kontrollieren. Ebenfalls löst die Mehrheit der Eltern Aufgaben mit ihren Kindern gemeinsam. Eher selten (16 % und 28 %) kommt die Nutzung von Videos und des Internets zum Einsatz. In der internationalen Elternbefragung von Vuorikari et al. (2020; vgl. Abb. 6b) zeigt sich, dass über alle drei DACH-Länder hinweg gratis Online-Lernmaterialien und nicht-digitale Medien wie Bücher am häufigsten von Eltern im Rahmen ihrer Lernunterstützung eingesetzt werden. Auch bei Grote (2020) verweisen rund 40 % der befragten Eltern darauf, dass sie zusätzliche Lernmedien nutzen; 30 % bearbeiten mit ihren Kindern zusätzlich eigene Themen. Schließlich berichten Wildemann und Hosenfeld (2020), dass 71 % der Eltern versuchen, ihr Kind im Homeschooling zu motivieren. Am häufigsten (50 % bzw. 46 %) greifen die Eltern dazu auf Appelle an Einsicht und Verständnis bzw. auf die gemeinsame Freizeitgestaltung (z. B. spielen, Filme sehen) zurück. Bei Holtgrewe et al. (2020a) berichten zwei Drittel der Schüler*innen, dass ihre Mütter sie trösten, wenn es schlecht läuft.

##### Konflikte, Streit und Spannungen im Fernunterricht

**Figure 7:**
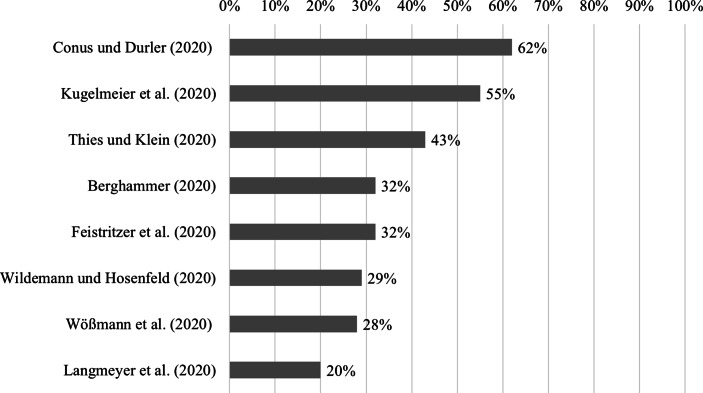

Dass die Lernbetreuung der Kinder durch ihre Eltern in vielen Fällen nicht immer reibungslos abläuft, lässt sich aus den in Abb. 7 dargestellten Befragungen belegen: Zwischen 20 und 62 % der Eltern geben an, dass es häufiger zu Streit bzw. Konflikten kommt oder die Beziehung zu ihren Kindern sehr oder ziemlich belastet ist. Auch 21 % der von Refle et al. (2020) befragten Schüler*innen geben an, dass es häufiger zu Spannungen zuhause komme. Jugend- und Familienstudien berichten dagegen von einer hohen Zufriedenheit der Kinder mit der Stimmung zuhause (Andresen et al. 2020) und davon, dass die Jugendlichen nicht häufiger als vor der Schulschließung aggressives verbales Verhalten oder gar Formen der körperlichen Gewalt ihrer Eltern wahrnehmen mussten (Baier und Kamenowski 2020). Mögliche Ursachen für Konflikte im Fernunterricht könnten darin liegen, dass sich Schüler*innen zu sehr kontrolliert fühlen oder zuhause viel für ihre Eltern erledigen müssen, wie 12 % bzw. 21 % der Schüler*innen in der Befragung von Huber et al. (2020) berichten. Auch geben 50 % der von Heller und Zügel (2020) befragten Schüler*innen an, dass die „Schule zu Hause“ ziemlich oder total durchgeplant ist, was zu einer hohen Belastung/Beanspruchung der Schüler*innen beitragen kann.

##### Unterschiede nach dem sozioökonomischen Hintergrund

In der Elternbefragung von ADAS und LIFE (2020) berichten vor allem alleinerziehende Eltern und Eltern mit Migrationshintergrund häufiger von fehlender Zeit für ihre Kinder (ADAS und LIFE 2020). Allerdings zeigen andere Elternbefragungen, dass der durchschnittlich investierte Zeitaufwand für die Lernbetreuung der Kinder nur geringfügig nach Bildungshintergrund der Eltern variiert (Thies und Klein 2020; Wößmann et al. 2020). Was die fachlichen Kompetenzen der Eltern betrifft, scheinen demografische Merkmale eine etwas wichtigere Rolle zu spielen: In der Schülerbefragung von Holtgrewe et al. (2020a) nimmt gut die Hälfte der Schüler*innen von *einfach*qualifizierten Eltern die Hilflosigkeit ihrer Eltern wahr; bei hochqualifizierten Eltern ist es nur jedes vierte Kind. In der ADAS und LIFE-Befragung (2020) zeigt sich, dass Alleinerziehende und Eltern mit Migrationshintergrund ihr Wissen, das sie zur Unterstützung ihrer Kinder bei den Aufgaben benötigen, deutlich häufiger (21 % bzw. 25 %) als wenig oder nicht geeignet einschätzen als andere Eltern (etwa 10 %). Auch bei Grote (2020) finden sich Hinweise, dass Eltern mit Migrationshintergrund häufiger Probleme haben, die inhaltliche Betreuung, insbesondere im Deutschunterricht, wahrzunehmen.

##### Unterschiede nach dem Schultyp

Insbesondere für den Betreuungsbedarf zeigen sich erwartungsgemäß Unterschiede in Anhängigkeit der Schüler*innen. So geben in der JIM plus-Befragung (Rathgeb 2020) 90 % der 12- bis 13-jährigen und nur 20 % der 16- bis 18-jährigen Schüler*innen an, dass sie Hilfe von ihren Eltern beim Lernen bekommen. Bei Holtgrewe et al. (2020a) reduziert sich dieser Anteil von 69 % (Volksschüler*innen) auf 16 % (Sek II-Schüler*innen).

### Merkmale der Qualität des Fernunterrichts

Die Befunde zu Aspekten, die als Qualitätsmerkmale von Fernunterricht vermutet werden, werden im Folgenden nach dem Modell der Basisdimensionen der Unterrichtsqualität (Klieme et al. 2009) gegliedert – auch wenn die Abgrenzung der Dimensionen nicht immer eindeutig ist. Die Basisdimensionen „hoher Anteil echter Lernzeit sowie Klarheit und Struktur“, „kognitive Aktivierung“ und „individuelle Lernunterstützung bzw. soziales Lernklima“ werden von Klieme (2020), Meyer (2020) und Voss und Wittwer (2020) als zentrale Kriterien zur Beurteilung der Qualität des *Fern*unterrichts verwendet.

#### Maßnahmen zur Sicherung echter Lernzeit sowie der Struktur und Klarheit

In den uns vorliegenden Befragungen wurden verschiedene Aspekte des Lehr-Lern-Prozesses im Fernunterricht abgefragt, von denen wir annehmen, dass sie dazu beitragen, die Lernzeit der Schüler*innen hoch zu halten.

Als Voraussetzung für einen hohen Anteil echter Lernzeit sehen wir einen hohen Anteil an erreichten Schüler*innen im Fernunterricht. Knapp 60 % der Lehrkräfte geben bei brlv (2020; Neumeier 2020) sowie Huber et al. (2020) an, 90–100 % der Schüler*innen zu erreichen. Bei Eickelmann und Drossel (2020) geben 35 % der Lehrkräfte an, alle Schüler*innen erreicht zu haben. Schließlich schätzen bei Seda und Ottacher (2020) sowie Schütz und Bestgen (2020) die Lehrkräfte ein, dass sie 80 % bzw. 87 % ihrer Schüler*innen erreichen. Dennoch sehen in der Lehrerbefragung von Steiner et al. (2020) je nach Schultyp 55 % bzw. 59 % der Lehrkräfte die Erreichbarkeit der Schüler*innen als Problemlage im Fernunterricht. Bei forsa (2020c) sehen 14 % die Erreichbarkeit der Schüler*innen als *größte* Herausforderung im Fernunterricht.

Besonders wichtig für die Lernzeit erscheint uns auch der *regelmäßige Kontakt und Austausch* der Lehrperson mit den Schüler*innen. Die internationale *Schülerbefragung* von Vuorikari et al. (2020) berichtet für Deutschland, Österreich und die Schweiz, dass 34 %, 41 % und 57 % der Schüler*innen täglich Kontakt mit ihren Lehrer*innen haben. 55 %, 53 % und 41 % haben nur wöchentlich Kontakt zu ihren Lehrer*innen. Allerdings dürften diese Befunde zwischen den Schulfächern erheblich variieren. So zeigen Heller und Zügel (2020), dass je nach Schulfach nur 21 % (Kunst/Musik) bis 55 % (Informatik) der Schüler*innen regelmäßigen Austausch mit der Lehrperson haben. Schließlich wird bei Huber et al. (2020) berichtet, dass für 12 % der Schüler*innen die Absprachen mit den Lehrkräften nicht funktionieren. Unter den Eltern meinen sogar 41 %, dass die Absprachen ein großes Problem auf Seiten der Schule darstellen (Petersen und Heimbach 2020). In den *Elternbefragungen* von Wößmann et al. (2020) und Steinmayr und Christiansen (2020) geben 45 % bzw. rund 50 % der Eltern an, dass ihr Kind während des Fernunterrichts nie persönlichen Kontakt (z. B. per Telefon) mit einer Lehrkraft hatte. Täglichen Kontakt mit der Lehrperson haben laut der Elternbefragung von Thies und Klein (2020) nur ein Fünftel der Schüler*innen. Ähnlich wie bei den Schüler*innen, haben die Lehrpersonen offenbar auch nur mit etwa der Hälfte der Eltern direkten Kontakt. So berichten Porsch und Porsch (2020), Steinmayr und Christiansen (2020) und Wildemann und Hosenfeld (2020), dass 48 %, mindestens die Hälfte und 63 % der Eltern keinen persönlichen Kontakt zu oder Austausch mit den Lehrkräften hatten. 63 % der Eltern haben allerdings die Kontaktdaten der Lehrkräfte erhalten, um sich im Bedarfsfall melden zu können (Porsch und Porsch 2020). Jene Eltern, die im Kontakt mit den Lehrkräften stehen, zeigen sich in der Mehrheit mit der Kommunikation zufrieden (62 % bei Thies und Klein 2020; etwa 50 % bei Grote 2020; knapp 50 % bei Landeselternrat Sachsen 2020). Aus *Sicht der Lehrkräfte* liegen ebenfalls Informationen zum Ausmaß des Schülerkontaktes vor. Bei forsa (2020c) verweisen 79 % der Lehrkräfte darauf, dass ihre Schüler*innen grundsätzlich jederzeit mit ihnen Kontakt aufnehmen können (bzgl. der Eltern sind es 86 %). Bei Dreer et al. (2020) standen mehr als die Hälfte (60 %) der Lehrkräfte drei bis fünf Mal in einer Woche in Kontakt mit ihren Schüler*innen. Bei Fobizz (2020) unterrichten knapp 30 % der Lehrer*innen ihre Schüler*innen im Fernunterricht täglich. In der Befragung von Eickelmann und Drossel (2020) geben nur 9 % der Lehrkräfte an, nur zu wenigen Schüler*innen in Verbindung zu stehen. Rund doppelt so hoch (20 %) ist der Anteil in der Lehrerbefragung von forsa (2020c). Über diese Befunde hinaus berichten Lorenz et al. (2020b), von einem sehr hohen Anteil (85 %) an Lehrpersonen, die sich regelmäßig einen Überblick über die Aktivitäten der Lernenden verschaffen. Neben der Quantität gibt es auch Hinweise zur Qualität der Lehrer-Schüler-Kommunikation: Ein hoher Lehreranteil zeigt sich (88 % bei Bildungsdirektion Nidwalden 2020; 77 % bei brlv 2020; Neumeier 2020) zufrieden mit der Kommunikation mit den Schüler*innen während des Fernunterrichts sowie mit der Kommunikation mit den Eltern (99 % bei Bildungsdirektion Nidwalden 2020; 63 % der Schulleiter*innen bei Jesacher-Rößler und Klein 2020; vgl. auch die im Durchschnitt als positiv eingeschätzte Lehrer-Eltern-Kommunikation bei Lorenz et al. 2020b).

Neben dem persönlichen Kontakt kommt auch der *Übermittlung von Lernmaterialen* hohe Bedeutung für den Anteil echter Lernzeit zu. In der ifo-Befragung (Wößmann et al. 2020) berichten 96 % der Eltern, dass ihre Kinder wöchentlich Aufgabenblätter erhalten. Dies deckt sich mit den 94 % der Eltern, die von Anger et al. (2020) dazu befragt wurden. Conus und Durler (2020) berichten dagegen von 20 % der Eltern, die Probleme haben, die Lernmaterialen zu erhalten. Der Anteil variiert je nach Schulfach stark: Wildemann und Hosenfeld (2020) berichten, dass 99 % der Eltern angeben für Mathematik Lernaufgaben von den Lehrkräften erhalten zu haben, während dies nur 10 % für das Fach Musik berichten.

Neben dem regelmäßigen Lehrer-Schüler/Eltern-Kontakt gehen wir davon aus, dass durch die Organisation und Durchführung *digital unterstützten Unterrichts *die Lernzeit der Schüler*innen gesteigert werden kann. Die Befunde zum Ausmaß des tatsächlich durchgeführten digitalen Unterrichts sind allerdings ernüchternd.

In der *Schülerbefragung* von Huber et al. (2020) berichten 31 % der Schüler*innen, dass ihre Lehrkräfte keinen digitalen Unterricht organisieren und 68 % bzw. 70 % keine digital unterstützte Live-Kommunikation nutzen bzw. keine Erklärvideos zur Verfügung stellen. Auch in der Schülerbefragung der Bildungsdirektion des Schweizer Kantons Nidwalden (2020) geben 38 % der Lernenden an, dass sie keinen gemeinsamen digitalen Unterricht hatten. Bei Wacker et al. (2020) fordern 63 % der befragten Schüler*innen mehr Videokonferenzen. Auch bei Holtgrewe et al. (2020a) fordern 30 % der Schüler*innen mehr Erklärungen der Lehrperson, um mit den Aufgaben besser zurecht zu kommen. Über alle drei Länder Deutschland, Österreich und die Schweiz hinweg berichten Vuorikari et al. (2020), dass nur 32 %, 36 % und 24 % der befragten Schüler*innen wöchentlich digitalen Lehrerkontakt und Onlineaktivitäten hatten. Täglichen Onlinekontakt und Onlineaktivitäten hatten gar nur 19 %, 16 % und 28 %.

Auch in *Elternbefragungen* zeigt sich ein nüchternes Bild zum Ausmaß des digitalen Unterrichts während der Schulschließung im Frühjahr 2020. Mehrere Elternbefragungen berichten von einer größeren Elterngruppe, zwischen 45 und 85 % der befragten Eltern, die angeben, dass kein oder kaum Onlineunterricht stattfindet (45 % bei Wößmann et al. 2020; 67 % bei Thies und Klein 2020; 75 % bei Grote 2020; bis zu 85 % bei Steinmayr und Christiansen 2020). Steinmayr und Christiansen (2020) zeigen, dass dieser Anteil zwischen den Schulfächern leicht variiert: Laut Elternangaben haben 74 % der Mathe-, 77 % der Deutsch-, 78 % der Englisch- und 85 % der Biologie‑/Sachunterrichtlehrkräfte noch keinen Unterricht per Videokonferenz durchgeführt. Etwas aus der Reihe fällt die Berliner Elternbefragung des Bezirkselternausschuss Mitte (2020). Hier berichten nur 30 % der Eltern von Grundschulkindern, dass keine digitalen Unterrichtsformen genutzt werden; in der Gemeinschaftsschule und dem Gymnasium sind es sogar nur rund 10 %. Der Anteil an Schüler*innen, der täglich an digitalem Unterricht teilnimmt, ist laut den Elternbefragungen von Thies und Klein (2020) sowie Wößmann et al. (2020) mit 7 % und 6 % relativ klein. Entsprechend verwundert nicht, dass bei Kugelmeier und Schmolze-Krahn (2020) 40 % der Eltern zusätzliche digitale Unterstützungsangebote wünschen.

Auch aus Sicht der Lehrer*innen ist der Einsatz digitalen Unterrichts wenig verbreitet. So berichten mehrere Befragungen (Bildungsdirektion Nidwalden 2020; Cordes 2020; forsa 2020c; Lorenz et al. 2020b; Schrammel et al. 2020; Steiner et al. 2020) von eher niedrigen Lehreranteilen, die digitalen (Präsenz‑)Unterricht umsetzen. Bspw. verweisen Cordes (2020) und forsa (2020c) darauf, dass etwas mehr als die Hälfte der befragten Lehrpersonen (56 % und 53 %) den Anteil an digitalen Medien im Unterricht/im Kollegium unter 40 %/25 % einschätzen. Bei Lorenz et al. (2020b) geben konkret nur 25 % der befragten Lehrkräfte an, dass sie digitalen Unterricht (z. B. in Form von Videokonferenzen) machen und nur 31 % geben an, dass Apps und digitale Anwendungen genutzt werden, mit denen die Schüler*innen ihren Lernprozess planen, dokumentieren und/oder reflektieren können. Leicht höher liegt der Anteil an Videokonferenzen im Unterricht bei Schrammel et al. (2020): 46 % der befragten Lehrkräfte halten Videokonferenzen via Skype, MS Teams oder Zoom ab. Wie komplex die Frage nach dem Einsatz digitaler Medien im Unterricht ist, zeigen die heterogenen Befunde von Steiner et al. (2020). Je nach Zweck des digitalen Unterrichts vereinbaren Lehrkräfte digitalen Präsenzunterricht in unterschiedlich starkem Ausmaß: Steiner et al. (2020) berichten von 20 % der Lehrkräfte, die Präsenzzeiten für Chats vereinbaren, bis rund 40 % der Lehrkräfte, die Präsenzzeiten für Individualfragen oder für Videokonferenzen vereinbaren. Ähnlich zeigt die Lehrerbefragung der Bildungsdirektion Nidwalden (2020), dass bspw. nur 15 % der Lehrkräfte Sprachnachrichten einsetzen und nur 30 % Videokonferenzen durchführen, während immerhin 52 % der Lehrkräfte Erklärvideos einsetzen.

Die *regelmäßige Überprüfung* der von den Schüler*innen zu bearbeitenden Lernaufgaben durch die Lehrperson vermuten wir als weitere Maßnahme, die die Schüler*innen beim Lernen hält. Je nach *Schülerbefragung* geben 61 % (Huber et al. 2020), 52 % (Letzel et al. 2020) bzw. je nach Schulfach 53–71 % (Heller und Zügel 2020) der Schüler*innen an, dass die Lernaufgaben durch die Lehrkräfte kontrolliert bzw. geprüft werden. Nur bei 13 % der Schüler*innen erfolgt keine Kontrolle durch die Lehrperson (Huber et al. 2020). Anders ist dies aus *Elternsicht:* Grote (2020) berichtet von rund 75 % der Eltern, die angeben, dass bisher nie Aufgaben ihrer Kinder kontrolliert wurden oder Feedback gegeben wurde. Aus *Lehrersicht* kontrollieren aber 96 % der Lehrkräfte die Bearbeitung der Lern- und Unterrichtsaufgaben durch die Schüler*innen sehr wohl (Schwab et al. 2020). Im Vergleich zu vor der Schulschließung tun dies laut der Luzerner Gymnasiallehrerbefragung (Schwerzmann und Frenzel 2020) nun 33 % der Lehrkräfte häufiger und 38 % seltener. Da die Trennung zwischen Kontrolle von Schülerarbeiten und dem Feedback zu Schülerarbeiten oft schwer ist, verweisen wir hier auf die Befunde zum Feedback in Abschn. 5.4.3.

Weiter sind* Struktur und Klarheit* Voraussetzungen dafür, dass Schüler*innen sinnvoll lernen können. Die Wahrung von Struktur und Klarheit scheint den Lehrpersonen zu gelingen, da aus *Schülersicht* je nach Unterrichtsfach nur 5 % (Biologie) bis 16 % (Physik) der von Heller und Zügel (2020) befragten Schüler*innen berichten, dass ihnen *nicht* klar ist, was sie im Fach machen sollen. Mit Blick auf die Klarheit der Arbeitsaufträge und Lernaufgaben geben sogar 92 % der Schüler*innen (Bildungsdirektion Nidwalden 2020) und je nach *Elternbefragung* immerhin 56–80 % der Eltern (etwa 80 % bei Grote 2020; 79 % bei Wildemann und Hosenfeld 2020; 73 % bei Letzel et al. 2020; 56 % bei Müller 2020) an, dass die *Arbeitsaufträge klar, übersichtlich und verständlich* gestaltet sind. Laut der Luzerner *Gymnasiallehrerbefragung* (Schwerzmann und Frenzel 2020) geben 38 % der Befragten an, dass sie im Vergleich zu vor der Schulschließung nun häufiger klare Strukturen vorgeben. Ein weiteres wichtiges Merkmal der Struktur und Klarheit im Fernunterricht stellen eine *klar kommunizierte Aufgabenvergabe und -abgabe *sowie *Lernpläne* dar. Bei Wildemann und Hosenfeld (2020) geben 42 % der Eltern an, dass sie keinen erkennbaren Rhythmus in der Aufgabenvergabe wahrnehmen. Hingegen geben 90 % der von Lorenz et al. (2020b) befragten Lehrkräfte an, dass sie feste Abgabetermine kommunizieren. Wochenpläne erhalten die Schüler*innen aus Sicht von 37 % der Eltern (Wildemann und Hosenfeld 2020) und aus Sicht von 47 % der Lehrkräfte (Huber et al. 2020).

##### Unterschiede nach dem sozioökonomischen Hintergrund

Bei Wößmann et al. (2020) geben deutlich mehr Eltern ohne akademischen Abschluss an, dass ihre Kinder nie Online-Unterricht hatten, als dies Eltern mit akademischem Abschluss tun. Bei Steiner et al. (2020) berichten für benachteiligte Schüler*innen deutlich mehr Lehrkräfte, dass sie diese im Fernunterricht *nicht* erreichen als dies Lehrkräfte für nicht-benachteiligte Schüler*innen angeben.

##### Unterschiede nach dem Schultyp

Die Befunde der Elternbefragung von Langmeyer et al. (2020) und der Lehrerbefragung von Fobizz (2020) enthalten Hinweise dazu, dass mit höheren Schultypen bzw. -stufen der Lehrer-Schüler-Kontakt abnimmt. Umgekehrt verhält es sich beim Ausmaß an digitalem Unterricht: Sowohl in Schülerbefragungen (Bildungsdirektion Nidwalden 2020; Trültzsch-Wijnen und Trültzsch-Wijnen 2020) als auch Eltern- (Bezirkselternausschuss Mitte 2020) und Lehrerbefragungen (forsa 2020c) wird von einem positiven Zusammenhang zwischen dem Ausmaß an Onlineunterricht und höheren Schultypen bzw. -stufen berichtet. Was die regelmäßige Überprüfung von bearbeiteten Lernaufgaben durch Lehrkräfte betrifft, so berichten Schwab et al. (2020) von *keinen* wesentlichen Unterschieden zwischen den Schultypen. Auch die Klarheit und Verständlichkeit der Lernaufgaben im Fernunterricht scheint sich aus Schüler- (Bildungsdirektion Nidwalden 2020) und Elternsicht (Müller 2020) *kaum* zwischen den Schultypen zu unterscheiden. Bzgl. dem Anteil erreichter Schüler*innen sind in den vorliegenden Befragungen *keine* systematischen Zusammenhänge mit dem Schultyp beobachtbar (Eickelmann und Drossel 2020; forsa 2020c; Steiner et al. 2020). Auch widersprechen sich die Befunde der für Deutschland repräsentativen Lehrerbefragungen, wonach einmal Gymnasiallehrkräfte zu jener Lehrergruppe gehören, die vergleichsweise wenige Schüler*innen erreichen (Eickelmann und Drossel 2020) und einmal zu jener Gruppe gehören, die vergleichsweise viele Schüler*innen im Fernunterricht erreichen (forsa 2020c).

#### Maßnahmen zur Sicherung der kognitiven Aktivierung

Inwiefern gestaltet sich Fernunterricht so, dass die Verstehensprozesse der Lernenden unterstützt werden? In der *Schülerbefragung* von Heller und Zügel (2020) werden Befunde zur Wissensvermittlung als auch zum eingesetzten *Aufgabentyp* berichtet: (1) Je nach Schulfach geben 40 % (Deutsch) bis 60 % (Informatik) der Schüler*innen an, dass ihnen gefällt, wie die Lehrkräfte die Wissensvermittlung organisieren. Eine motivierende Wissensvermittlung gilt als verständnisförderlich. In diesem Zusammenhang berichten auch Baier und Kamenowski (2020), dass 57 % der Schüler*innen der Aussage zustimmen, dass ihre Lehrpersonen abwechslungsreichen Fernunterricht gestalten. (2) Die von Heller und Zügel (2020) befragten Schüler*innen geben an, dass die geforderten Lernaufgaben fast ausschließlich in die Kategorien „Aufgaben lösen“ und „Texte lesen“ fallen und damit eher repetitive Tätigkeiten, die oft wenig verständnisfördernd sind, von den Schüler*innen verlangt werden. Dies deckt sich mit den Befunden zweier *Elternbefragungen*. In der ifo-Befragung (Wößmann et al. 2020) erwies sich die Bereitstellung von zu bearbeitenden Aufgabenblättern als die häufigste Lehraktivität während der Schulschließung: 87 % der Eltern gaben an, dass die Schüler*innen mehrmals wöchentlich bereitgestellte Aufgaben bearbeiten. In der Elternbefragung von Wildemann und Hosenfeld (2020) berichten 76 %, dass selten oder nie kreative Aufgaben gestellt werden. Darüber hinaus geben über 60 % der Eltern für die Fächer Mathematik und Deutsch an, dass die Aufgaben wenig bis gar nicht abwechslungsreich sind; dies gilt sowohl für die Grundschule als auch für weiterführende Schulen. In der Mehrheit der Fälle berichten die Eltern, dass Lernaufgaben (50 % in der Grundschule, 40 % in weiterführenden Schulen) sowohl als Übung als auch zum Erlernen von neuen Inhalten eingesetzt werden. Ähnliches berichten Wößmann et al. (2020): Laut Elternangaben wurde bei 47 % der Schüler*innen hauptsächlich neuer Unterrichtsstoff vermittelt und bei einem ähnlich großen Anteil (45 %) hauptsächlich bereits bekannter Unterrichtstoff wiederholt. Dies gilt unabhängig vom Bildungshintergrund und von den schulischen Leistungen der Schüler*innen. Auch aus Lehrersicht stellen Arbeitsblätter das mit großem Abstand am häufigsten genutzte Format dar (84 %; forsa 2020c). Zudem zeigen Schwerzmann und Frenzel (2020), dass Lehrpersonen häufiger als vor dem Schul-Lockdown „Texte verfassen und gestalten“ lassen.

Neben dem Aufgabentyp wird in der Literatur (z. B. Kunter und Voss 2013; Renkl 1997) häufig auch *kooperatives Lernen* als – wenn richtig umgesetzt – förderlich für die kognitive Aktivierung der Lernenden angesehen. In der *Schülerbefragung* von Heller und Zügel (2020) findet sich der Hinweis, dass das Ausmaß an kooperativem Lernen im Fernunterricht je nach Schulfach stark variiert: 24 % (Kunst/Musik) bis 63 % (Physik) der Schüler*innen geben an, regelmäßigen Austausch mit den Mitschüler*innen zu haben. Fachunabhängig berichten in der JIMplus-Befragung (Rathgeb 2020) 50 % der Jugendlichen mit Freunden über Chats zu lernen. Leicht seltener wird kooperatives Lernen aus *Elternsicht* wahrgenommen. Befragungen berichten von 31 % (Müller 2020) bzw. 40 % (Wildemann und Hosenfeld 2020) der Eltern, die meinen, dass sich ihr Kind mindestens einmal pro Woche mit Mitschüler*innen austauscht. Dies erscheint wenig, vor dem Hintergrund des hohen Anteils an Eltern (88 % bei Thies und Klein 2020), die berichten, dass ihren Kindern der Austausch mit Klassenkameraden und Lehrkräften fehlt. Und vor dem Hintergrund des Befundes der internationalen Befragung von Vuorikari et al. (2020), der zeigt, dass in Deutschland, Österreich und der Schweiz 63 %, 73 % und 73 % der befragten Eltern meinen, dass die Schulen mehr kooperative Onlineelemente einsetzen könnten, um ihre Kinder besser zu unterstützen. Das eher moderate Kooperationsniveau im Fernunterricht wird auch aus *Lehrersicht* bestätigt. Bei Huber et al. (2020) und Schwab et al. (2020) berichten 40 % bzw. 45 % der Lehrkräfte, dass sie ihren Schüler*innen *keine* technischen Möglichkeiten zum unterrichtlichen Austausch anbieten.

Dagegen scheint ein weiteres Merkmal kognitiv aktivierenden Unterrichts relativ positiv ausgeprägt zu sein: die *Verfügbarkeit der Lehrperson für Fragen*. Nur rund 10 % der von Huber et al. (2020) bzw. 12 % der von Schreiner et al. (2020) befragten Schüler*innen führen an, dass sie ihre Lehrkräfte *nicht* immer fragen können, bspw. wenn sie beim Lernen nicht weiterkommen (vgl. auch die hohen Zustimmungsraten bei Grütter et al. 2020). Auch wissen nur 3 % der von Schober et al. (2020a) befragten Schüler*innen nicht, wie sie ihre Lehrpersonen bei Fragen erreichen können. Dies deckt sich mit der *Elternsicht*: Nur 10 % (Wößmann et al. 2020) bzw. 15 % (Müller 2020) der Eltern geben an, dass ihr Kind während der Schulschließung *nicht* die Möglichkeit hatte, seine Lehrer*innen etwa für Hilfe bei den Aufgaben direkt zu kontaktieren (z. B. per Telefon, E‑Mail oder WhatsApp) oder nicht die Unterstützung erhielt, wenn es diese benötigte. Dagegen geben in der Elternbefragung in der französischsprachigen Schweiz (Conus und Durler 2020) 54 % an, dass ihre Kinder im Fernunterricht nicht die Möglichkeit hatten, ihre Lehrer*innen zu kontaktieren. Aus *Lehrersicht* geben fast alle Befragten (99 %) an, dass sie für Rückfragen zur Verfügung stehen (Lorenz et al. 2020b).

##### Unterschiede nach dem sozioökonomischen Hintergrund

Bzgl. der Aufgabentypen zeigen sich bei Wößmann et al. (2020) *keine* sozioökonomisch bedingten Unterschiede. Auch bzgl. der Erreichbarkeit der Lehrperson für Schüler*innen bei Lernproblemen beobachten Wößmann et al. (2020) *keine* Unterschiede nach der sozialen Herkunft. Dagegen berichten Baier und Kamenowski (2020), dass für Schüler*innen mit niedrigerem Sozialstatus seltener Lehrpersonen als Ansprechpersonen bei Problemen zur Verfügung stehen.

##### Unterschiede nach dem Schultyp

Bzgl. der kognitiven Aktivierung durch abwechslungsreichen Fernunterricht berichten Wildemann und Hosenfeld (2020) von vernachlässigbaren Schultypenunterschieden und Baier und Kamenowski (2020) davon, dass Gymnasialschüler*innen seltener als Schüler*innen anderer Schultypen angeben, dass der Unterricht abwechslungsreich ist. Kooperatives Lernen im Fernunterricht ist erwartungsgemäß deutlich stärker in höheren Schulstufen ausgeprägt (Baier und Kamenowski 2020; Müller 2020; Schwab et al. 2020). Schließlich beobachtet Müller (2020) *keine* Schultypenunterschiede bzgl. der Verfügbarkeit der Lehrpersonen für die Lernunterstützung der Schüler*innen.

#### Maßnahmen zur Sicherung der individuellen Lernunterstützung

Diese Basisdimension ist u. a. durch die positive *Lehrer-Schüler-Beziehung* sowie die individuelle *Betreuung* der Lernenden gekennzeichnet. In dieser Hinsicht weist der Fernunterricht einen immanenten Mangel gegenüber dem Regelunterricht auf (vgl. Klieme 2020). Allerdings nicht für alle befragten Personen, wie die bestehenden Befragungen zeigen (vgl. dazu auch die Befunde in Abschn. 5.4.2 zur Verfügbarkeit der Lehrpersonen im Fernunterricht für Schülerfragen und in Abschn. 5.4.1 zum Lehrer-Schüler-Kontakt). In der Befragung von Letzel et al. (2020) wünschen sich 49 % der Schüler*innen mehr Unterstützung durch die Lehrperson. Auch berichten 51 % (Letzel et al. 2020), 48 % (Baier und Kamenowski 2020), 40 % (Lochner 2020) und 34 % (Wacker et al. 2020) der Schüler*innen, dass ihnen die Lehrperson fehlt. Dabei dürften sie insbesondere die Gespräche vermissen, wie 30 % der Schüler*innen bei Holtgrewe et al. (2020a) angeben. Grundsätzlich funktioniert die Kommunikation mit den Lehrkräften aber gut, wie die Luzerner Schülerbefragung (Schwerzmann und Frenzel 2020) belegt: Rund 90 % der Schüler*innen geben an, dass diese gut funktioniert hat. Auch stimmen 49 % der bei Refle et al. (2020) befragten Schüler*innen der Aussage zu, dass ihre Lehrpersonen sich sehr bemüht haben, ihnen beim Lernen zu helfen. Auch aus *Elternsicht* fehlt den Kindern der Kontakt zu ihren Lehrer*innen. Bei Thies und Klein (2020) berichten 88 % der Eltern, dass ihren Kindern besonders der persönliche Austausch mit ihren Lehrkräften fehlt. In der für Deutschland repräsentativen Elternbefragung von Wößmann et al. (2020) geben 67 % der Eltern an, dass ihr Kind weniger als einmal pro Woche individuellen Kontakt mit einer Lehrkraft hatte; 45 % hatten nie individuellen Kontakt. Auch in der Hamburger Elternbefragung von Müller (2020) geben „nur“ 44 % der Eltern an, dass die Lehrkräfte ausreichend persönlichen Kontakt zu ihrem Kind halten. Demgegenüber stehen 72 % der von Feistritzer et al. (2020) befragten Eltern, die angeben, dass die Lehrkräfte bemüht sind, auf ihr Kind einzugehen. Auch schätzen 63 % der an der ADAS und LIFE-Befragung teilnehmenden Eltern die Betreuung ihrer Kinder durch die Lehrkräfte und die Schule als gut oder sehr gut ein (ADAS und LIFE 2020). Ebenso schätzen rund 50 % der in der sächsischen Befragung (Landeselternrat Sachsen 2020) befragten Eltern die Kommunikation zwischen Lehrer*innen und Schüler*innen gut ein. Entsprechend sieht „nur“ etwa ein Drittel (29 %) der Eltern den fehlenden Kontakt mit der Lehrperson als Hindernis für erfolgreichen Fernunterricht (Tengler et al. 2020). Die Informationen aus den *Lehrerbefragungen* zur Lehrer-Schüler-Beziehung zeigen folgendes Bild: Nach Eickelmann und Drossel (2020) ist 87 % der Lehrer*innen wichtig mit ihren Schüler*innen persönlich in Kontakt zu bleiben. 53 % der von Tengler et al. (2020) befragten *Lehrkräfte* geben an, dass ihre Schüler*innen die gleiche Unterstützung wie sonst auch bekommen. Bei brlv (2020; Neumeier 2020) berichten rund 70 % der Lehrkräfte, dass die Beziehung zu ihren Schüler*innen genauso gut wie zuvor sei. 16 % der Befragten sehen sogar ein verbessertes Verhältnis zu ihren Schülern seit den Schulschließungen. Auch bei Schwerzmann und Frenzel (2020) berichten 16 % der befragten Gymnasiallehrer*innen, dass sie mehr in Beziehungsarbeit zu einzelnen Schüler*innen investieren als im Präsenzunterricht. Bei Ziegler und Hannemann (2020) hat für 8 % der Lehrkräfte die Quantität und für 20 % die Qualität des Lehrer-Schüler-Verhältnisses zugenommen. Sowohl aus Lehrer- als auch aus Schulleitersicht wird die Lehrer-Schüler-Interaktion im Fernunterricht überaus positiv wahrgenommen: Bei Spiel und Holzer (2020) geben 93 % der Lehrkräfte an, dass die persönlichen Beziehungen zu den Schüler*innen während des Unterrichtens von zu Hause aus überwiegend positiv waren. Ein ähnlich hoher Anteil (84 %) fühlte sich trotz der Situation mit den Schüler*innen verbunden. Bei Jesacher-Rößler und Klein (2020) berichten 66 % der befragten Schulleiter*innen, dass die Lehrer-Schüler-Interaktion positiv oder mehr positiv als negativ wahrgenommen wird. Zudem stimmen bei Huber et al. (2020) 85 % der Lehrer*innen und 93 % der Schulleitungen der Aussage „Die Befindlichkeit und Sorgen der Schülerinnen und Schüler werden von der Schule ernst genommen.“ zu.

Als etwas spezifischere Form der Schülerbetreuung lässt sich die *Differenzierung und Individualisierung* im Fernunterricht in den Blick nehmen. Indizien für das Ausmaß der Differenzierung und Individualisierung im Fernunterricht finden sich vor allem bei Holtgrewe et al. (2020a), Letzel et al. (2020), Huber et al. (2020) und Schwab et al. (2020). Holtgrewe et al. (2020a) berichten, dass die Schüler*innen im Fernunterricht mehr Freiheiten wahrnehmen: freiere Zeiteinteilung (83 %); stärkere Auswahl beim Lernstoff (31 %) und bei den Lerninhalten (16 %) sowie mehr Zeit zum Üben (51 %). Letzel et al. (2020) haben eine Skala zur Binnendifferenzierung (Pozas et al. 2020) bei einer kleineren Gruppe von Schüler*innen, Eltern und Lehrkräften eingesetzt. Der überwiegenden Mehrheit der Schüler- und Elternangaben nach findet Differenzierung im Fernunterricht durch Aufgaben (Schüler*innen: 62–86 %; Eltern: 79–80 %) und durch Arbeitsgruppen (91–93 %; 95–97 %) *eher selten bis nie* statt. Dagegen schätzen die Lehrkräfte die Binnendifferenzierung deutlich stärker ausgeprägt ein. Nur 41–66 % bzw. 75–86 % geben an, dass Binnendifferenzierung im Fernunterricht durch Aufgaben bzw. durch Gruppenbildung *eher selten bis nie* stattfindet. Auch Schwab et al. (2020) haben die Lehrkräfte mit einer Skala zur Individualisierung des Fernunterrichts befragt. Den Befunden nach zeigen 75 % der Lehrkräfte Interesse an den Lebenslagen ihrer Schüler*innen und 86 % meinen diese auch im Fernunterricht zu berücksichtigen. Der Lehreranteil der Individualisierungsmaßnahmen im Fernunterricht variiert zwischen 61 % (Themen der Lernpakete werden individualisiert) und 99 % (Schüler*innen für individuelle Fragen zur Verfügung zu stehen). Ähnlich wie bei Letzel et al. (2020) wurden auch bei Huber et al. (2020) Schüler*innen, Eltern und Lehrkräfte zur Individualisierung im Fernunterricht befragt. 49 % der Schüler*innen und 32 % der Eltern stimmen der Aussage zu, dass der Lernstand der Schüler*innen Berücksichtigung findet. 45 % der Schüler*innen und 26 % der Eltern geben an, dass die Lernenden differenzierte Hinweise zu den bearbeiteten Lernaufgaben bekommen. 30 % der Lehrpersonen berichten von einem wöchentlichen, individuellen Schüler-Coaching. In der Befragung von Luzerner Gymnasiallehrer*innen (Schwerzmann und Frenzel 2020) sagen sogar 35 %, dass sie mehr individuelles Coaching als im Präsenzunterricht machen.

In einigen Untersuchungen wurde auch konkret nach der *Passung der Lernaufgaben* gefragt. Aus *Schülersicht* empfindet rund die Hälfte bis 60 % die Lernaufgaben als nicht zu schwer (63 % bei Letzel et al. 2020; 50 % bei Schreiner et al. 2020) oder kommt mit den Lernaufgaben ohne Hilfe zurecht (53 % in Chemie bis 67 % in Informatik, Heller und Zügel 2020). Im Umkehrschluss dürfte eine nicht kleine Gruppe an Schüler*innen die Lernaufgaben als Herausforderung wahrnehmen. Beispielsweise finden 37 % (Letzel et al. 2020), 22 % (Holtgrewe et al. 2020a) und 13 % (Schreiner et al. 2020) der befragten Schüler*innen die Aufgaben zu schwer oder hatten Schwierigkeiten bei ihrer Bewältigung. Die Befunde aus *Elternsicht* sind relativ einheitlich. In mehreren Befragungen stufen zwischen 60–71 % der Eltern den Schwierigkeitsgrad, die Komplexität und die Menge der Lernaufgaben als angemessen für ihre Kinder ein (68 % bei Feistritzer et al. 2020; 60 % bzw. 67 % Letzel et al. 2020; 71 % bei Müller 2020). Etwas aus der Reihe fallen die Befunde der Elternbefragungen im Kanton Nidwalden (86 % bei Bildungsdirektion Nidwalden 2020) und dem Bundesland Sachsen (33 % bei Landeselternrat Sachsen 2020; vgl. auch die niedrigen Werte bei Grote 2020). Schließlich berichten Ziegler und Hannemann (2020) von 50 % der Eltern, die angeben, dass ihre Kinder weniger gut mit den Aufgaben zurechtkommen als im Präsenzunterricht; 35 % berichten, dass sie diese auch weniger gut verstehen würden. Aus Lehrersicht sind diese Anteile mit 66 % und 50 % nochmal deutlich höher. Dies obwohl in *Lehrerbefragungen* 59 % der Lehrkräfte angeben, eher bis sehr häufig Aufgaben und Materialien einzusetzen, die ihrer Schwierigkeit nach abgestuft sind (Letzel et al. 2020), und 75 % der Lehrkräfte angeben, Inhalte und Aufgaben an den individuellen Lernstand ihrer Schüler*innen anzupassen (Lorenz et al. 2020b). Siehe dazu auch die oben berichteten Befunde zur Individualisierung von Lernpaketen (Schwab et al. 2020).

Neben der Schülerbetreuung und der Individualisierung nimmt das *Feedback* eine zentrale Rolle für die individuelle Lernunterstützung ein. Im Fernunterricht empfinden Schüler*innen ausführliches schriftliches Feedback als besonders hilfreich, wie 79 % in der Schülerbefragung von Holtgrewe et al. (2020a) angeben. Die Mehrheit der Schüler*innen dürfte diese Hilfe auch bekommen, denn je nach Schulfach meinen „nur“ 5 % (Informatik) bis 20 % (Kunst/Musik) der Schüler*innen *kein* Feedback zu erhalten (Heller und Zügel 2020). Auch bei Wacker et al. (2020) und Bildungsdirektion Nidwalden (2020) berichten „nur“ 16 % bzw. 13 % der Schüler*innen, dass sie von ihren Lehrer*innen selten oder nie Feedback auf gelöste Lernaufgaben bekommen. Aus *Elternsicht* ist die Lage zum Feedback deutlich anders. Die Befunde der Elternbefragungen sind zwar relativ heterogen, aber die Mehrheit verweist auf deutlich höhere Elternanteile, die kein Feedback wahrnehmen. Etwa ein Drittel bis drei Viertel der *Eltern* geben an, dass ihr Kind *kein* Feedback von der Lehrperson erhält. Während in manchen Befragungen (60 % bei Grote 2020; 58 % bei Wildemann und Hosenfeld 2020; je nach Fach 59–74 % bei Steinmayr und Christiansen 2020) deutlich mehr als die Hälfte der Eltern von fehlendem Feedback berichten, sind dies in der Hamburger Elternbefragung (32 %; Müller 2020) und in der ifo-Elternbefragung (17 % nie, 36 % weniger als einmal pro Woche; Wößmann et al. 2020) deutlich weniger. Die unterschiedlichen Befunde könnten auf nicht-identische Operationalisierungen in den Befragungen zurückzuführen sein. Laut *Lehrer*daten geben 96 % der Lehrkräfte an, dass sie die Bearbeitung der Lern- bzw. Unterrichtsaufgaben durch die Schüler*innen kontrollieren (Grote 2020). In der Studie von Lorenz et al. (2020b) sind es 84 % der Lehrkräfte, die Feedback auf die Ergebnisse der Lernenden geben und 82 % der Lehrkräfte, die Musterlösungen zur Verfügung stellen.

##### Unterschiede nach dem sozioökonomischen Hintergrund

Bei Baier und Kamenowski (2020) vermissen Schüler*innen mit niedrigem bzw. mittlerem Sozialstatus ihre Lehrkräfte seltener als Schüler*innen mit hohem Sozialstatus. Bzgl. der Passung der Lernaufgaben zum Lernstand der Schüler*innen berichten Holtgrewe et al. (2020a), dass Schüler*innen einfach qualifizierter Eltern und bilinguale Schüler*innen deutlich häufiger Schwierigkeiten bei der Bewältigung der Lernaufgaben haben als andere Schülergruppen. Schließlich berichten bei Wößmann et al. (2020) Eltern mit akademischem Bildungsabschluss deutlich häufiger von Lehrerfeedback und individuellen Lehrer-Schüler-Gesprächen als Eltern ohne akademischen Bildungsabschluss dies tun.

##### Unterschiede nach dem Schultyp

- Lehrer-Schüler-Beziehung im Fernunterricht: Schwerzmann und Frenzel (2020), Müller (2020) und Eickelmann und Drossel (2020) können *keine* Schultypenunterschiede in der Qualität der Lehrer-Schüler-Kommunikation bzw. im Lehrer-Schüler-Kontakt ausmachen. Lediglich bei Holtgrewe et al. (2020a) und bei forsa (2020c) finden sich Hinweise, dass Schüler*innen der Volksschule ihre Lehrer*innen häufiger vermissen und in höheren Schultypen Schüler*innen häufiger jederzeit in Kontakt mit der Lehrperson treten können.
- Differenzierung und Individualisierung im Fernunterricht: Schwab et al. (2020) und Feistritzer et al. (2020) berichten, dass in Gymnasien deutlich weniger oft Lernpakete für das Lernen zuhause individualisiert werden und Lehrer*innen deutlich seltener bemüht sind auf die Lage der Schüler*innen einzugehen als dies in anderen Schultypen der Fall ist. Holtgrewe et al. (2020a) berichten darüber hinaus, dass Schüler*innen der Sekundarstufe II deutlich mehr freie Zeiteinteilung im Fernunterricht wahrnehmen als Schüler*innen anderer Schulstufen.
- Passung der Lernaufgaben im Fernunterricht: Bei Müller (2020) und Feistritzer et al. (2020) zeichnet sich die Tendenz ab, dass in Grund- bzw. Volksschulen die Lernaufgaben häufiger über eine angemessenere Schwierigkeit verfügen bzw. weniger überfordernd sind als in anderen Schultypen. Darüber hinaus berichtet die kantonale Befragung der Bildungsdirektion Nidwalden (2020) von einem leicht negativen Zusammenhang zwischen einem angemessenen Zeitaufwand für Lernaufgaben und höheren Schulstufen.
- Feedback im Fernunterricht: In den vorliegenden Befragungen (Bildungsdirektion Nidwalden 2020; Müller 2020) sind *keine* systematischen Schultypenunterschiede auszumachen.

#### Maßnahmen zur Unterstützung der Eltern (eine 4. Basisdimension)

Ein weiteres Merkmal guten Fernunterrichts kann in der Elternunterstützung gesehen werden. Da die Eltern zentrale Funktionen der Lehrpersonen übernehmen müssen (z. B. die individuelle Lernunterstützung beim Lernen zuhause) hängt der Erfolg des Fernunterrichts auch stark vom Ausmaß und der Qualität der elterlichen Unterstützung ab (siehe Abschn. 5.3.2; vgl. Köller et al. 2020) – bei jüngeren Schüler*innen stärker als bei älteren Schüler*innen. Schulen und Lehrkräfte sollten daher danach trachten, die Eltern in ihrer neuen Rolle während des Fernunterrichts zu unterstützen. Mehrere *Elternbefragungen* weisen darauf hin, dass es einen moderaten Anteil an Eltern (7–45 %) gibt, die sich im Fernunterricht von der Schule und den Lehrkräften ihrer Kinder *nicht* ausreichend oder *schlecht* unterstützt fühlen (7 % bei Feistritzer et al. 2020; 23 % bei Porsch und Porsch 2020; 32 % bei Lochner 2020).^20^ Die Art der von den Schulen und Lehrpersonen gewährten Unterstützung betreffend zeigen Heller und Zügel (2020), dass – über alle Schulstufen hinweg – von den meisten Eltern (etwa 40–50 %) Strukturierungs- und Organisationshilfen sowie technische Unterstützung wahrgenommen werden (vgl. dazu auch die 47 % der Eltern bei Conus und Durler (2020), die angeben Informationen darüber erhalten zu haben, wie sie die Schularbeit ihres Kindes organisieren können). Rund 10 % der Eltern haben keine Unterstützungsangebote bekommen. Auch bei Porsch und Porsch (2020) und Conus und Durler (2020) finden sich Hinweise, dass 28 % bzw. 16 % der Befragten neben den Aufgaben keine weitere Unterstützung oder Informationen von den Lehrkräften über die pädagogische Beaufsichtigung der Arbeit ihres Kindes erhalten haben. Entsprechend wünschen sich 39 % der von Thies und Klein (2020) befragten Eltern eine bessere Organisation und Unterstützung durch die Schule und Lehrkräfte für das Lernen zuhause. Im Gegensatz zum Unterstützungsangebot dürfte das Informationsangebot der Schulen zufriedenstellend sein. Zumindest für die Eltern des Kantons Nidwalden: 95 % geben an, dass sie von der Schule/der Lehrperson informiert wurden, wie der Fernunterricht abläuft (Bildungsdirektion Nidwalden 2020). Neben allgemeiner Elternunterstützung verweisen Köller et al. (2020) konkret auf das Modell der elterlichen Lernunterstützung nach Dumont et al. (2014), das empfiehlt, dass Eltern die Befriedigung der drei psychologischen Grundbedürfnisse ihrer Kinder nach sozialer Eingebundenheit, Kompetenzerleben und Autonomieerleben im Sinne der Selbstbestimmungstheorie (SDT, Deci und Ryan 1993) unterstützen sollten. Eltern sollten daher auf das richtige Ausmaß von Ansprechbarkeit, Strukturierung und Kontrolle achten (siehe Köller et al. 2020 zur Beschreibung dieser Aspekte im Fernunterricht). Inwiefern dies Eltern gelingt, ist mangels Forschungsstand noch unklar. Erste Befunde zur elterlichen Lern- und Motivationsunterstützung wurden in Abschn. 5.3.2 berichtet. Die von Vuorikari et al. (2020) befragten Eltern aus Deutschland, Österreich und der Schweiz sind allerdings der Meinung, dass die Schulen mehr Richtlinien und Hinweise geben sollten, wie sie ihre Kinder beim Fernunterricht unterstützen können (64 %, 66 %, 66 %) und wie sie ihre Kinder psychologisch unterstützen können (54 %, 52 %, 50 %).

##### Unterschiede nach dem sozioökonomischen Hintergrund

Es liegen keine Befunde vor.

##### Unterschiede nach dem Schultyp

Die Elternbefragungen kommen zu einem heterogenen Bild der Befundlage. Während in zwei Befragungen (Bezirkselternausschuss Mitte 2020; Heller und Zügel 2020) Eltern von Grundschulen am häufigsten davon berichten im Fernunterricht keine oder kaum Unterstützung von der Schule oder den Lehrkräften zu erhalten, berichten Feistritzer et al. (2020), dass sich Eltern von Volksschulkindern im Vergleich zu Eltern von Kindern anderer Schultypen am häufigsten von den Lehrer*innen unterstützt fühlen. Bzgl. des Informationsangebotes für Eltern sind *keine* relevanten Schultypenunterschiede beobachtbar (Bildungsdirektion Nidwalden 2020).

#### Globale Qualitätseinschätzung des Fernunterrichts

Manche Befragungen erheben eine allgemeine Einschätzung der Qualität des Fernunterrichts. Aus *Schülersicht* finden 52–71 % den Fernunterricht gut oder sehr gut oder sind der Meinung, dass dieser gut oder sehr gut geklappt hat (71 % bei Bildungsdirektion Nidwalden 2020; 52 % bei Rathgeb 2020; je nach Schultyp 54 % (Gymnasium) bis 65 % (Volksschule) bei Schwerzmann und Frenzel 2020; je nach Fach 40 % (Deutsch) bis 60 % (Informatik) bei Heller und Zügel 2020). Auch aus *Elternperspektive* wurde nach der Qualität des Fernunterrichts gefragt. In mehreren Befragungen berichten sehr unterschiedlich große Elternanteile, von 28–75 % der Eltern, dass der Fernunterricht gut funktioniere (30 % bei Petersen und Heimbach 2020; 41 % bei Tengler et al. 2020; 53 % bei Berghammer 2020; 44 % (Grundschule) bis 61 % (Gymnasium) bei Heller und Zügel 2020; etwa 75 % bei Grote 2020) oder mit dem Homeschooling zufrieden sind (etwa 28 % bei Steinmayr und Christiansen 2020; 38 % bei Müller 2020; 56 % bei Wößmann et al. 2020). Als „gut (auf die Situation eingestellt)“ oder „qualitativ hochwertig“ beurteilen den Fernunterricht deutlich mehr als die Hälfte der Eltern (56 % bei Letzel et al. 2020; 74 % der Volksschullehrer*innen bei Schwerzmann und Frenzel 2020; rund 50 % (Gesamtschule) bis 85 % (Gemeinschaftsschule) bei Bezirkselternausschuss Mitte 2020). In der Civey-Befragung (2020) bewerteten dagegen nur 19 % der befragten Eltern das digitale Bildungsangebot an den Schulen während der Pandemie als gut, während knapp 60 % mit dem Angebot unzufrieden waren. In den meisten hier angeführten Befragungen ist die Unzufriedenheit mit dem Fernunterricht unter den Eltern deutlich niedriger ausgeprägt. Auch aus Lehrerperspektive ist die Einschätzung der allgemeinen Qualität des Fernunterrichts je nach Befragung sehr unterschiedlich: Schwerzmann und Frenzel (2020) berichten von 47 % (Sonderschule) bis 68 % (Volksschule) der befragten Lehrkräfte, die sagen, dass die Lernerfahrungen im Fernunterricht eher gut oder sehr gut waren. Bei Tengler et al. (2020) bewerten knapp mehr als die Hälfte der Lehrpersonen den Fernunterricht als gut bis ausgezeichnet. In der Lehrerbefragung von Seda und Ottacher (2020) sind es nur etwa 36 %, die meinen, dass der Fernunterricht gut bis hervorragend funktioniert. Auffällig ist auch der Befund der Schulleiterbefragung im Kanton Nidwalden (Bildungsdirektion Nidwalden 2020), in der alle 37 befragten Schulleiter*innen der Aussage zustimmen, dass die Lehrpersonen an ihrer Schule den Auftrag zum Fernunterricht gut umgesetzt haben.

##### Unterschiede nach dem sozioökonomischen Hintergrund

Während die Elternbefragungen von Müller (2020) und Wößmann et al. (2020) *keine* Unterschiede in der allgemeinen Qualitätseinschätzung des Fernunterrichts nach dem Bildungshintergrund der befragten Eltern ausmachen können, berichtet Berghammer (2020), dass Eltern mit Matura oder Hochschulabschluss deutlich häufiger als Eltern mit maximal Lehrabschluss angeben, dass der Fernunterricht gut funktioniert.

##### Unterschiede nach dem Schultyp

Die Befunde zu allgemeinen Qualitätseinschätzungen des Fernunterrichts nach Schultyp zeigen ein heterogenes Bild. Bei Rathgeb (2020) und Müller (2020) werden keine systematischen und relevanten Unterschiede zwischen den Schultypen beobachtet. Bei Heller und Zügel (2020) beurteilen Eltern von Gymnasialschüler*innen den Fernunterricht im Durchschnitt besser als Eltern von Grundschüler*innen. Dies ist bei Schwerzmann und Frenzel (2020) umgekehrt. In der Elternbefragung des Berliner Bezirkselternausschuss Mitte (2020) schneidet die Gemeinschaftsschule deutlich besser als andere Schultypen ab.

### Lehrerkompetenzen im Fernunterricht

Zur Frage nach den *Lehrerkompetenzen* für den Fernunterricht liegen nahezu ausschließlich Befunde aus Lehrerselbsteinschätzungen vor. So schätzen in der DACH-Lehrerbefragung von Huber et al. (2020) 31 % das Kollegium als nicht ausreichend kompetent für den Einsatz digitaler Lehr-Lern-Formen ein. Auch bei Eickelmann und Drossel (2020) und Lorenz et al. (2020b) bewerten 10 % bzw. 38 % der befragten Lehrkräfte die eigenen Kompetenzen als nicht ausreichend, um Lernangebote im Fernunterricht (ausreichend gut) bereitstellen zu können. Hinsichtlich des Umgangs mit digitalen Medien schreiben sich bei Dreer et al. (2020) 13 % der Lehrer*innen niedrige Kompetenzen zu. Mangelnde digitale Kompetenzen sehen 24 % der Lehrkräfte (Schrammel et al. 2020; Tengler et al. 2020) als Hindernis im Fernunterricht; 69 % sehen sie als „größten Verbesserungsbedarf“ (forsa 2020c). Aus Sicht der Schulleitungen werden die digitalen Kompetenzen der Lehrkräfte dagegen mehrheitlich nicht als Herausforderung für den Fernunterricht wahrgenommen (Huber et al. 2020; Jesacher-Rößler und Klein 2020).

#### Erfahrung mit digitalen Medien

37 % der Lehrkräfte geben an, bereits seit längerer Zeit in der Schule mit digitalen Medien zu arbeiten (Huber et al. 2020). 88 % bzw. 62 % haben bereits vor dem Fernunterricht digitale Medien (z. B. PC, Notebook) im Unterricht bzw. zur Unterrichtsvorbereitung eingesetzt (Dreer et al. 2020). Bei forsa (2020c) geben 44 % der Lehrkräfte an, dass die Hälfte oder mehr ihrer Kolleg*innen bereits vor Corona mindestens einmal pro Woche digitale Medien im Unterricht eingesetzt haben.

#### Vorbereitung auf den Fernunterricht

Mehr als die Hälfte der Lehrkräfte gibt an, dass sie überhaupt nicht oder eher schlecht auf das Homeschooling vorbereitet war (Cordes 2020) oder die plötzliche Schulschließung sie bezüglich digitaler Lehr-Lern-Formen vor große Herausforderungen stellte (60 % bei Huber et al. 2020). Auch bei Eickelmann und Drossel (2020) lehnen 60 % die Aussage „In unserer Schule war die Nutzung digitaler Möglichkeiten schon vor der Schließung recht weit fortgeschritten, daher waren wir auf eine solche Situation verhältnismäßig gut vorbereitet.“ ab. 33 % stimmen der Aussage zu.

#### Lehrermotivation

Dreer et al. (2020) berichten, dass 60 % der befragten Lehrer*innen auch in der gegenwärtigen Situation mit ihrem Beruf zufrieden sind. Blickt man auf Befunde zur Motivation für den Einsatz digitaler Lehr-Lern-Formen/Medien im Unterricht, so stimmen bei Huber et al. (2020) und bei Dreer et al. (2020) 46 % bzw. 40 % der Lehrkräfte zu, dass sie bzw. ihre Kolleg*innen diesbezüglich motiviert sind. Bei Schrammel et al. (2020; vgl. auch Tengler et al. 2020) nutzen gar rund 80 % der befragten Lehrkräfte *gerne* Lernplattformen und digitale Medien für das Homeschooling. Allerdings haben 20 % eher nicht vor, nach der Phase des Homeschoolings Unterricht weiterhin mit digitalen Medien durchzuführen. In der Umfrage von forsa (2020c) orten zudem 35 % der Lehrkräfte Verbesserungsbedarf bei der Bereitschaft von Lehrkräften, digitale Lernformate im Unterricht einzusetzen.

#### Lehrerselbstwirksamkeit

Während für 97 % der Berufseinsteiger*innen und 73 % der erfahrenen Lehrpersonen das Bedienen digitaler Medien (eher) einfach ist (Schrammel et al. 2020), geben nur 25 % der Lehrkräfte an, dass es ihnen leicht fällt, mit digitalen Medien in der aktuellen Situation Lernprozesse zu gestalten (Huber et al. 2020). Allerdings gibt der Großteil der Lehrkräfte (72 % bei Spiel und Holzer 2020; 81 % bei Eickelmann und Drossel 2020; 81 % bei forsa 2020c) an, dass sie mit dem Unterrichten von zu Hause bzw. der neuen Situation gut zurechtgekommen sind.

Aus *Schüler- und Elternsicht* wird von rund je einem Drittel den Lehrpersonen hohe Kompetenz (32 % Schüler*innen, 32 % Eltern) und Motivation (34 % Schüler*innen; 46 % Eltern) für den Einsatz digitaler Lehr-Lern-Formen attestiert (Huber et al. 2020). Gleichzeitig werden von fast jedem zweiten Elternteil (45 %) die digitalen Kompetenzen der Lehrkräfte als großes Problem aufseiten der Schulen ausgemacht (Petersen und Heimbach 2020).

#### Unterschiede nach dem sozioökonomischen Hintergrund

Bei Eickelmann und Drossel (2020) wird vermutet, dass der in nichtgymnasialen Schulen (im Vergleich zu Gymnasien) beobachtete höhere Anteil an Lehrkräften, die berichten, sich mit der neuen Situation überfordert zu fühlen, auf den höheren Anteil an Schulen mit einer besonders herausfordernden Schülerschaft in diesem Schultyp zurückzuführen sei.

#### Unterschiede nach dem Schultyp

Lehrerbefragungen zeigen, dass die Vorbereitung auf den Fernunterricht und die Selbstwirksamkeit der Lehrkräfte (forsa 2020c; Eickelmann und Drossel 2020) sowie die Lehrermotivation (Schwerzmann und Frenzel 2020) positiv mit höheren Schulstufen bzw. Schultypen assoziiert ist. Anders scheint dies bzgl. dem Lehrerwissen über die Schülersituation zuhause zu sein. Hier beobachten Schwab et al. (2020) einen negativen Zusammenhang zwischen dem Lehrerwissen und der Schulstufe bzw. dem Schultyp.

### Technologieeinsatz im Fernunterricht

**Figure 8:**
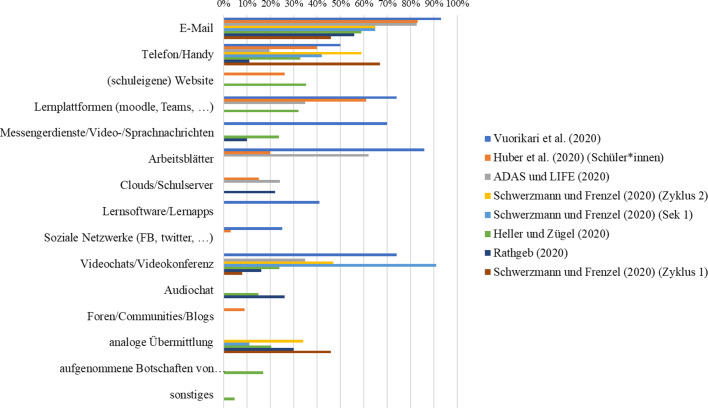

Die Frage, mit welchen Medien Schulen und Lehrkräfte im Fernunterricht versucht haben, ihre Schüler*innen zu erreichen, stellt eine der am häufigsten gestellten Fragen in den vorliegenden Befragungen dar (siehe Abb. 3). Das hohe Interesse ist vermutlich auf den Umstand zurückzuführen, dass die Schulschließung binnen weniger Tage erfolgte, sie viele Schulen unvorbereitet traf und die Nutzung digitaler Medien für den (Fern‑)Unterricht vielerorts noch nicht etabliert war. Wie Abb. 8, 9 und 10 zeigen, berichten alle Personengruppen, dass das E‑Mail mit Abstand am häufigsten zum Einsatz kam (46–93 % bei den Schülerbefragungen; 42–86 % bei den Elternbefragungen; 48–92 % bei den Lehrerbefragungen). Über alle Befragungen hinweg geben im Mittel 70 % der Befragten an, dass das E‑Mail als Kommunikationsmittel im Fernunterricht eingesetzt wird. Dahinter folgen Lernplattformen (45 %) und das Telefon/Handy (42 %). Schließlich nennen im Durchschnitt aller Befragungsgruppen jeweils rund 30 % der Personen, dass Videochats und -konferenzen, Messengerdienste, die (schuleigene) Website und die analoge Übermittlung als weitere Kommunikationsmedien im Fernunterricht zum Einsatz kommen. Korreliert man die berichteten Anteile mit dem Enddatum der Befragung, so zeigt sich nur für den Einsatz von Videokonferenzen eine positive, mittlere Korrelation in allen drei Befragungsgruppen (*r* = 0,53; *N* = 18 Befragungen), was auf eine tatsächliche Zunahme des Einsatzes dieses Kommunikationsmediums mit Fortlauf des Fernunterrichts hindeutet. Allerdings müsste hier zumindest für die Schultypen kontrolliert werden.

**Figure 9:**
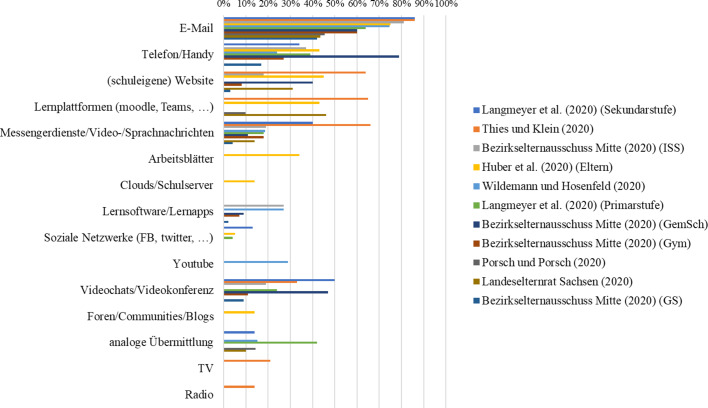

**Figure 10:**
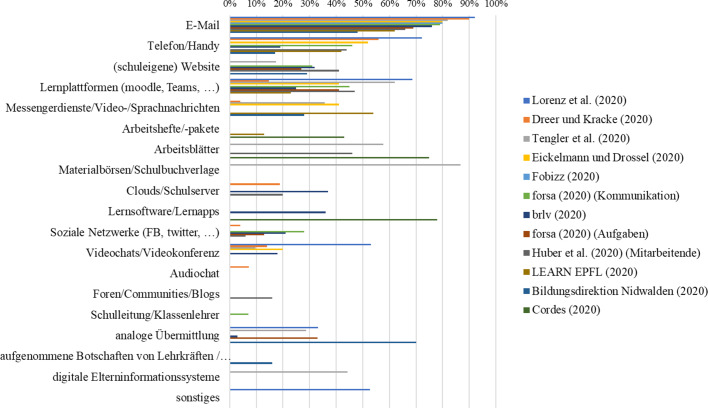

#### Unterschiede nach dem sozioökonomischen Hintergrund

Es liegen keine Befunde vor.

#### Unterschiede nach dem Schultyp

Erwartungsgemäß zeigen die meisten Schüler- (Schwerzmann und Frenzel 2020), Eltern- (Bezirkselternausschuss Mitte 2020; Langmeyer et al. 2020; Thies und Klein 2020) und Lehrerbefragungen (Eickelmann und Drossel 2020; forsa 2020c), dass digitale Tools im Fernunterricht in der Primarstufe deutlich seltener zum Einsatz kommen. Dies zeigt sich insbesondere im geringeren Einsatz von Videokonferenztools; aber auch bereits bei der Verwendung von E‑Mails. Entsprechend wird in der Primarstufe Lernmaterial häufiger analog übermittelt und die Kommunikation mit den Schüler*innen erfolgt etwas häufiger via Mobiltelefon. In den genannten Befragungen korreliert neben der Häufigkeit des Einsatzes von Videokonferenztools auch die Häufigkeit des Einsatzes von Lernplattformen, Cloud- und Messenger-Diensten positiv mit höheren Schulstufen bzw. Schultypen.

## Diskussion

Ziel dieses Reviews ist es, einen ersten systematischen Überblick über den Forschungsstand zum Lehren und Lernen während des coronabedingten Fernunterrichts zu geben. Der Review macht deutlich, dass bereits eine Fülle von Informationen zu zentralen Aspekten des Fernunterrichts vorliegen; bisher aber in überwiegend nicht publizierter Form in einschlägigen Fachorganen oder durch einschlägige Verlage. Der vorgelegte Review versucht die Befundlage in strukturierter und systematischer Weise auch entlang methodologischer und theoretischer Überlegungen aufzubereiten. Damit leistet er für Forscher*innen, die sich gegenwärtig und künftig mit dem Thema wissenschaftlich beschäftigen, einen wichtigen Service: Das wissenschaftliche Ethos erfordert die Wahrung des bereits erreichten theoretischen und empirischen Wissens. Wie der vorliegende Review zeigt, liegt für das Forschungsfeld „Schule und COVID-19“ bereits umfangreiches empirisches Wissen vor. Der Review stellt daher Befragungen, Quellen und exemplarische Befunde dar. Zukünftige Studien können deutlich effizienter erfolgen, wenn der aktuelle empirische Wissensstand Berücksichtigung findet. Darüber hinaus liefert der Review eine relevante Informationsbasis für Entscheidungen und für das Handeln in den jeweiligen Verantwortungsbereichen der Politik, Verwaltung und Schulpraxis.

### Zusammenfassung der Erkenntnisse des Reviews

#### Design der Befragungen

##### Ziele der Befragungen

Alle Befragungen weisen einen sehr stark ausgeprägten situationsanalytischen Charakter auf. Die Befragungsziele werden meist, wenn überhaupt, sehr vage dargestellt.

##### Durchführende Organisationen

Ein kleinerer Teil der Befragungen (22), der aber hinsichtlich des Stichprobeumfangs substanziell ist, wurde von Organisationen durchgeführt, die üblicherweise nicht wissenschaftlich tätig sind (z. B. Eltern- und Lehrerverbände).

##### Theorie

In den meisten Fällen werden keine Informationen über die der jeweiligen Befragung zugrundeliegenden theoretischen Konzepte gegeben. Dort wo Bezüge zu Theorien hergestellt werden, sind diese meist knapp und/oder sehr abstrakt gehalten.

##### Konstruktoperationalisierung

Nur sehr selten werden Skalen eingesetzt. Die allermeisten Befragungen verwenden Einzelindikatoren zur Erfassung von Konstrukten. Am häufigsten (in 32 Ergebnisberichten) wurde die Frage nach der technischen Ausstattung der Schüler*innen zuhause gestellt.

##### Erhebungsmethode

Alle Befragungen wurden im Zeitraum vom 24. März bis 11. November 2020 durchgeführt und stellen Online-Befragungen dar. Die Rekrutierung der befragten Personen erfolgte meist über die Aussendung des Links zur Online-Befragung via E‑Mailverteiler.

##### Stichprobe

Die 97 Befragungen umfassen 255.955 Fälle – davon 60.468 Schüler*innen, 151.660 und 41.350 Lehrkräfte sowie 2339 Schulleitungen. Dabei verteilen sich die erfassten Fälle wie folgt auf die Länder: 124.485 (Deutschland), 36.417 (Österreich), 84.497 (Schweiz), 10.322 (DACH). Die meisten Forschungsinitiativen fokussieren dabei ein (54) oder zwei (9) Zielgruppen. Neun Initiativen umfassen mehr als zwei Zielgruppen und versuchen zu ähnlichen Fragestellungen eine multiperspektivische Sichtweise zu berichten. 18 Befragungen verfügen über eine repräsentative Stichprobe.

##### Analysemethoden

Alle Befragungen berichten primär deskriptive Statistiken. In einigen Fällen werden auch Gruppenvergleiche (z. B. benachteiligte vs. nicht-benachteiligte Schülergruppen) auf Basis deskriptiver Analysen ohne inferenzstatistische Absicherung berichtet. Allerdings befanden sich zum Zeitpunkt der Manuskripterstellung mehrere Beiträge mit multivariaten Analysen im Begutachtungsprozess.

##### Dissemination

Einige wenige Befragungen wurden bereits relativ früh in peer-reviewten bildungswissenschaftlichen Publikationsorganen (z. B. Die Deutsche Schule, Educational Assessment Evaluation and Accountability) veröffentlicht. Die Mehrheit der Befragungen wurde allerdings via Online-Report veröffentlicht.

#### Inhaltliche Befunde der Befragungen

##### Lernerfolg

Die vorliegenden Befragungen zeigen auf, dass – laut Selbsteinschätzung der Schüler*innen – etwa ein Fünftel bis die Hälfte negative Auswirkungen des Fernunterrichts auf den eigenen Lernerfolg erwarten. Werden Eltern nach dem Lernrückstand ihrer Kinder befragt, sind es rund ein Drittel bis zwei Drittel, die negative Auswirkungen befürchten. Auch etwas mehr als ein Drittel der Lehrkräfte befürchtet negative Effekte auf das Schülerlernen.

##### Lernaufwand

Der Forschungsstand zum Lernaufwand der Schüler*innen während des Fernunterrichts ist von hoher Heterogenität geprägt. Der Anteil an Schüler*innen, die weniger als 2 h pro Tag für das Lernen aufwenden, variiert je nach Befragung zwischen rund einem Fünftel und etwas mehr als der Hälfte der Schüler*innen. Darüber hinaus geben in den Schülerbefragungen rund ein Drittel bis knapp die Hälfte an, dass ihr Workload während des Fernunterrichts geringer als vor dem Lockdown war. Diese Befunde decken sich im Wesentlichen mit jenen der Elternbefragungen.

##### Lernmotivation

Etwa rund ein bis zwei Drittel der Schüler*innen berichtet, gerne oder sehr gerne im Fernunterricht zu lernen. Je nach Befragung beklagen allerdings rund ein Viertel bis die Hälfte der Eltern und bis zu 70 % der Lehrkräfte die fehlende Lernmotivation ihrer Kinder/Schüler*innen im Fernunterricht.

##### Selbstständigkeit der Schüler*innen

Etwas mehr als einem Drittel der Schüler*innen fällt die Selbstorganisation ihres Tagesablaufes (z. B. früh aufstehen) schwer. Zudem berichten etwa ein Viertel bis ein Drittel der Schüler*innen von Schwierigkeiten, Konzentrationsproblemen und Überforderung beim selbstgesteuerten Lernen. Bzgl. der Nutzung digitaler Medien attestieren sich je nach Anwendungsbereich zwischen der Hälfte und über 80 % der Schüler*innen gute Fähigkeiten. Aus Elternperspektive berichten in der Mehrheit der Befragungen etwa ein Viertel bis ein Drittel der Eltern, dass ihre Kinder Schwierigkeiten beim selbstgesteuerten Lernen haben. Die Befunde der Lehrerbefragungen sind diesbezüglich sehr heterogen. Je nach Befragung schätzen rund 40–90 % der Lehrkräfte das selbstständige Arbeiten und die Selbstorganisation der Schüler*innen als Herausforderung für den Fernunterricht ein. Gleichzeitig bewerten in einer großangelegten Schweizer Befragung nahezu alle Lehrkräfte das selbstständige Arbeiten ihrer Schüler*innen als zuverlässig.

##### Technische Ausstattung zuhause

Je nach Befragung berichten rund 3 % bis ein Viertel der Schüler*innen und Eltern, dass die technische Ausstattung zuhause eine Herausforderung für das Lernen im Fernunterricht darstellt. Es ist sehr wahrscheinlich, dass dieser Befund den tatsächlichen Anteil an Schüler*innen mit mangelhafter technischer Ausstattung zuhause unterschätzt, da anzunehmen ist, dass gerade diese Schülergruppe mit Online-Befragungen – insbesondere in den nicht repräsentativen Befragungen (siehe Abschn. 4.6) – nur bedingt erreicht wird. Aus Lehrersicht wird die technische Ausstattungssituation der Schüler*innen deutlich negativer beschrieben. Rund 30–70 % der Lehrkräfte bemängeln diese.

##### Elterliche Unterstützung im Fernunterricht

Die elterliche Unterstützung ist gerade für junge Kinder mit noch gering ausgeprägten Selbstlernfähigkeiten im Fernunterricht zentral. Eltern von Kindern der Primarstufe und der Sekundarstufe I berichten viermal häufiger als Eltern älterer Kinder, dass sie ihre Kinder beim Lernen unterstützen. Die elterliche Unterstützung wird dabei in etwa 80 % der Fälle von Müttern geleistet. Aus Schülersicht erhalten je nach Befragung ein Fünftel bis ein Drittel der Schüler*innen zuhause nicht die notwendige Lernunterstützung von ihren Eltern. Je nach Befragung geben ein Viertel bis zwei Drittel der Eltern an, dass ihnen dazu die Zeit fehlt; u. a. aufgrund der Berufstätigkeit und weiterer Kinder. Je nach Befragung geben rund ein Viertel bis zwei Drittel der Eltern an, durchschnittlich weniger als 1 h pro Tag für die Lernunterstützung ihrer Kinder Zeit zu haben. Auch aus Lehrersicht wird von rund 40 % der Lehrkräfte die fehlende Elternunterstützung als Herausforderung für den Fernunterricht beklagt. Eine weitere Hürde für den Fernunterricht ist das fehlende Fachwissen der Eltern, wie 15 % bis rund ein Drittel der befragten Eltern meinen. Auch geben je nach Elternbefragung rund ein Zehntel bis knapp die Hälfte der Eltern an mit der Betreuung allgemein überfordert zu sein. Was die Gestaltung der elterlichen Lernunterstützung betrifft, so nutzen Eltern am häufigsten gratis Online-Lernmaterialien und gratis Online-Lernprogramme sowie analoge Lernmaterialien. Die Lernunterstützung besteht zudem meist darin, die Korrektheit und Vollständigkeit der Bearbeitung der Lernaufgaben zu kontrollieren. Zu starke Kontrolle kann aber zu Reibungen zwischen Kindern und Eltern führen, wovon je nach Befragung 20 bis rund 60 % der Eltern berichten.

##### Echte Lernzeit im Fernunterricht

Um im Fernunterricht Schüler*innen beim Lernen zu halten, ist es wichtig, dass die Lehrpersonen die Schüler*innen überhaupt erreichen, regelmäßig Kontakt zu den Schüler*innen und Eltern halten, digitalen Live-Unterricht abhalten, klar verständliche Lernaufgaben einsetzen und diese auch kontrollieren. Den vorliegenden Befragungen zufolge gibt es hier Verbesserungspotenzial. Je nach Befragung berichten Lehrkräfte, dass sie mit ihrem Unterrichtsangebot zwischen 70 und 90 % ihrer Schüler*innen im Fernunterricht erreichen. Von regelmäßigem Lehrerkontakt berichten nur ein Fünftel bis rund die Hälfte der Schüler*innen. Zudem berichtet etwa die Hälfte der Eltern, dass ihr Kind während des Fernunterrichts nie persönlichen Kontakt mit einer Lehrkraft hatte. Auch der Lehrer-Eltern-Kontakt ist laut mindestens der Hälfte der Eltern nicht gegeben. Aus Lehrersicht stehen 30–60 % täglich in Kontakt mit den Schüler*innen, rund 10–20 % geben an, mit nur wenigen Schüler*innen in Verbindung zu stehen. Die Übermittlung von Lernmaterialen als weitere wichtige Voraussetzung für einen hohen Anteil an echter Lernzeit funktioniert aus Elternsicht in nahezu 100 % der Fälle. Auch deuten die Befragungen darauf hin, dass Lehrkräfte – zumindest in der ersten Phase der Schulschließungen – kaum digitale Lehr-Lern-Formate (z. B. Videokonferenztools zur Live-Kommunikation, Erklärvideos) eingesetzt haben. So berichten zwischen rund 40 % und rund 70 % der Schüler*innen, keinen digitalen Unterricht gehabt zu haben. Auch Eltern berichten zu 45–85 %, dass kein oder kaum Onlineunterricht stattfand. Auch aus Sicht der Lehrer*innen ist der Einsatz digitalen Unterrichts wenig verbreitet. So berichten je nach Umfrage zwischen rund 20 und 40 % der Lehrkräfte digitale Medien im Fernunterricht einzusetzen. Die Wahrung von Struktur und Klarheit im Fernunterricht als weitere Maßnahmen zur Sicherung der Lernzeit scheint den Lehrkräften gut zu gelingen. Je nach Fach und Befragung berichten rund 80–90 % der Schüler*innen und rund 60–80 % der Eltern, dass der Unterricht und die Arbeitsaufträge klar und verständlich sind. Die regelmäßige Kontrolle der von den Schüler*innen zu bearbeitenden Lernaufgaben durch die Lehrperson erfolgt aus Schülersicht in etwa der Hälfte bis rund 70 % der Fälle. Aus Lehrersicht erfolgt dies deutlich häufiger.

##### Kognitive Aktivierung im Fernunterricht

Zentrale Merkmale der kognitiven Aktivierung (z. B. kognitiv anregende und herausfordernde Lernaufgaben, kooperatives Lernen) werden im Fernunterricht bisher eher selten eingesetzt. Dies berichten abhängig vom Fach rund 60 bis über 70 % der Schüler*innen. Auch kreative Aufgaben kommen laut rund drei Viertel der Eltern selten zum Einsatz. Aus Sicht von rund 80 % der Lehrkräfte stellen Arbeitsblätter das mit großem Abstand am häufigsten genutzte Format dar. Vor dem Hintergrund dieser Befunde ist zu befürchten, dass sich der Fernunterricht zu sehr auf das repetitive, mechanische Abarbeiten von Lernaufgaben fokussiert und die Potenziale des sozialen Lernens zu wenig ausschöpft (vgl. dazu auch Klieme 2020).

##### Individuelle Lernunterstützung im Fernunterricht

Die *Lehrer-Schüler-Beziehung* betreffend berichten etwa ein Drittel bis die Hälfte der Schüler*innen, dass ihnen der Kontakt zu ihren Lehrer*innen fehlt. Auch substanzielle Anteile der Eltern, etwa zwischen einem Drittel und der Hälfte, beklagen, dass die Lehrpersonen nicht ausreichend Kontakt zu den Schüler*innen halten. Aus Lehrer- und Schulleitersicht wird dagegen die Lehrer-Schüler-Interaktion im Fernunterricht überaus positiv wahrgenommen. *Differenzierung und Individualisierung* im Fernunterricht durch Aufgaben (60–80 %) und durch Arbeitsgruppen (über 90 %) finden laut der überwiegenden Mehrheit (60–100 %) der Schüler*innen und Eltern eher selten bis nie statt. Dagegen schätzen die Lehrkräfte die Differenzierung und Individualisierung deutlich stärker ausgeprägt ein. Unabhängig davon scheint es den Lehrer*innen gut zu gelingen Lernaufgaben an das Niveau der Schüler*innen anzupassen. Aus Schülersicht empfindet ein hoher Anteil die Lernaufgaben als nicht zu schwer (etwa 50–70 %). Die Befunde aus Elternsicht sind ähnlich. So stufen zwischen 60–70 % der Eltern den Schwierigkeitsgrad, die Komplexität und die Menge der Lernaufgaben als angemessen für ihre Kinder ein. Auch das *Feedback* als weitere Individualisierungsmaßnahme im Fernunterricht dürfte gut funktionieren. Je nach Schulfach meinen „nur“ 5–20 % der Schüler*innen kein Feedback zu erhalten. Aus Elternsicht ist die Lage deutlich anders. Etwa ein Drittel bis drei Viertel der Eltern geben an, dass ihr Kind kein Feedback von der Lehrperson erhält. Dies widerspricht den Lehrerangaben: Über 80 % der Lehrkräfte geben an, Feedback zu geben.

##### Unterstützung der Eltern im Fernunterricht

Die Elternunterstützung im Fernunterricht ist gerade für den Lernerfolg jüngerer Schüler*innen zentral. Mehrere Elternbefragungen weisen darauf hin, dass es einen moderaten Anteil an Eltern (etwa rund 10 bis rund 50 %) gibt, der sich im Fernunterricht von der Schule und den Lehrkräften ihrer Kinder nicht unterstützt fühlt. Im Gegensatz zum Unterstützungsangebot dürfte das Informationsangebot der Schulen zufriedenstellend sein. Neben der allgemeinen Elternunterstützung verweisen Köller et al. (2020) darauf, dass Eltern die psychologischen Grundbedürfnisse (Autonomie, Kompetenz, soziale Eingebundenheit) besonders beachten sollten. In diesem Zusammenhang jedoch monieren etwa 50–70 % der Eltern, dass die Schulen mehr Richtlinien und Hinweise geben sollten, wie sie ihre Kinder (psychologisch) unterstützen können.

##### Lehrerkompetenzen im Fernunterricht

10–30 % der Lehrkräfte schätzen ihre eigenen *Kompetenzen* für den Fernunterricht und jene der Kolleg*innen als nicht ausreichend ein. Hinsichtlich des Umgangs mit digitalen Medien schreiben sich ebenfalls etwa 10 % bis ein Viertel niedrige Kompetenzen zu. Gleichzeitig berichtet die Mehrheit (60–90 %) der Lehrkräfte bereits vor der Schulschließung, Erfahrung im Einsatz digitaler Medien im Unterricht gesammelt zu haben; und rund 40 % arbeiten bereits seit längerer Zeit mit digitalen Medien. Dennoch gibt mehr als die Hälfte der Lehrkräfte an, nicht oder schlecht auf das Homeschooling vorbereitet gewesen zu sein. Bzgl. der Lehrermotivation und der Lehrerselbstwirksamkeit im Fernunterricht geben mehrere Befragungen an, dass etwa knapp die Hälfte (40–50 %) der Lehrkräfte motiviert sind und rund drei Viertel (70–80 %) geben an, gut mit der neuen Situation zurecht zu kommen. Aus *Schüler- und Elternsicht* wird von rund je einem Drittel den Lehrpersonen hohe Kompetenz und Motivation für den Einsatz digitaler Lehr-Lern-Formen attestiert.

##### Technologieeinsatz im Fernunterricht

In allen Personengruppen berichten zwischen rund 40–90 % der Befragten, dass das E‑Mail mit Abstand am häufigsten als Kommunikationsmedium im Fernunterricht zum Einsatz kam. Dahinter folgen mit deutlichem Abstand an zweiter Stelle die Lernplattformen und das Mobiltelefon. Schließlich folgen an dritter Stelle Videochats und -konferenzen, Messengerdienste, die (schuleigene) Website sowie die analoge Übermittlung von bspw. Lernaufgaben.

##### Unterschiede nach dem sozioökonomischen Hintergrund

Folgende Kategorien des Fernunterrichts erweisen sich in den identifizierten Befragungen als erwartungsgemäß positiv abhängig vom sozioökonomischen Hintergrund der Schüler*innen: Lernerfolg, Lernmotivation, Selbstständigkeit der Schüler*innen, technische Ausstattung zuhause, Kompetenz der Eltern für die Lernunterstützung.

##### Unterschiede nach dem Schultyp

Folgende Kategorien des Fernunterrichts weisen einen positiven Zusammenhang mit höheren Schultypen auf: Selbstständigkeit und Unterstützungsbedarf der Schüler*innen, Ausmaß des digital unterstützten Fernunterrichts, kooperatives Lernen, Individualisierung im Fernunterricht, Lehrermotivation, Technologieeinsatz im Fernunterricht. Die Aspekte Lehrer-Schüler-Kontakt und Lehrerwissen hängen dagegen negativ mit höheren Schultypen zusammen.

### Kritische Reflexion der Aussagekraft der Befunde und des Reviews

Neben der Berücksichtigung des bisherigen Forschungsstandes fordert ein weiteres wissenschaftliches Ethos einen kritischen Umgang mit den bisherigen Befunden. Wir werfen daher im Folgenden einen kritischen Blick auf die wissenschaftliche Qualität des skizzierten Forschungsstandes. Allerdings soll dabei nicht vergessen werden, dass die bisherige Forschung unter zwei Rahmenbedingungen zustande kam: (1) Die Schulschließungen erfolgten vielerorts sehr kurzfristig. Forscher*innen hatten im besten Fall einige wenige Tage/Wochen Zeit, um Befragungen, die eine Momentaufnahme der aktuellen Situation liefern, zu planen und durchzuführen. Je nach zeitlichen und finanziellen Ressourcen konnte in dieser knappen Zeit wissenschaftlichen Qualitätskriterien mehr oder weniger entsprochen werden. (2) Es ist darauf hinzuweisen, dass in den hier gesammelten Befragungen die Daten nicht immer vor dem Hintergrund wissenschaftlicher Ziele (siehe Abschn. 4.2) erhoben wurden und daher wissenschaftliche Gütekriterien (z. B. die theoretische Fundierung, Repräsentativität) unter Umständen nicht im Fokus standen.

#### Verallgemeinerbarkeit der Befunde

Die Verallgemeinerbarkeit von Studienergebnissen, also die Frage, ob Rückschlüsse auf die Gesamtpopulation möglich sind, hängt wesentlich von der Repräsentativität der untersuchten Stichprobe ab. Globale Repräsentativität (d. h. in Bezug auf alle Merkmale einer Stichprobe) kann in der Regel nur in Zufallsstichproben erwartet werden, für deren Ziehung ein Stichprobenrahmen – d. h. eine Liste mit Kontaktdaten aller Einheiten (Personen, Schulen …) der Grundgesamtheit – nötig ist. Merkmalsspezifische Repräsentativität (z. B. in Bezug auf Geschlecht oder Bildungshintergrund) versucht man u. a. auch durch Quoten bei der Stichprobenziehung oder durch Gewichtung (Poststratifikation, d. h. die Anpassung der realisierten Stichprobe an bekannte Merkmalsverteilungen der Grundgesamtheit) zu erreichen.

Bisher sind nur wenige Befragungen bekannt (vgl. Abschn. 4.6), die über eine repräsentative Stichprobe verfügen. Die meisten Online-Befragungen bzw. deren Ergebnisse sind in der Regel nicht als repräsentativ anzusehen. Diesbezüglich sind u. a. folgende Gründe anzuführen: (a) Personen ohne (ausreichenden) Zugang zu digitalen Medien und Internet sind per se von der Befragung ausgeschlossen. (b) Aufgrund von fehlenden Stichprobenrahmen werden Links zu Online-Befragungen meist über Websites oder per Mail („Schneeballverfahren“, d. h. Versenden von Mails mit der Bitte um Weiterleitung) verbreitet, wodurch sehr wahrscheinlich nicht die gesamte Grundgesamtheit erreicht wird. (c) Die Teilnahmeraten an Befragungen variieren häufig in Abhängigkeit von soziodemografischen Merkmalen, wie dem Bildungshintergrund oder auch einem Migrationshintergrund bzw. der (zuhause) gesprochenen Sprache. Durch diese Selbstselektion ist davon auszugehen, dass insbesondere jene Kinder und Familien, die vermutlich am stärksten von den Schulschließungen betroffen sind, in den bisherigen Befragungen unterrepräsentiert sind.

Inwiefern berichtete Befunde aus den Online-Befragungen verallgemeinerbar sind, ist mit Blick auf die jeweilige Stichprobenzusammensetzung kritisch zu prüfen. In der Beurteilung von Studien sollten folgende Punkte berücksichtigt werden: (a) Wie wurde die Stichprobe realisiert? (b) Wird die realisierte Stichprobe in Hinblick auf merkmalsspezifische Repräsentativität geprüft? (c) Erfolgt im Falle einer Abweichung (z. B., wenn Kinder aus bildungsfernen Familien unterrepräsentiert sind) eine Gewichtung der Daten? Tab. 3 und das Online-Zusatzmaterial enthalten diesbezügliche Informationen, die den Leser*innen erlauben, die Verallgemeinerung der Befunde einzuordnen.

#### Reliabilität und Validität von Messungen

Online-Befragungen müssen in ihrem Umfang kurzgehalten werden, um einerseits überhaupt Personen der Zielgruppe für die Teilnahme zu gewinnen und um andererseits einen Dropout während der Befragung zu vermeiden. Daher werden häufig Einzelindikatoren zur Erfassung von Konstrukten wie dem erlebten Stress eingesetzt, um den Fragebogen kurz zu halten. Diese Limitation lässt sich aber nur schwer mit Ansprüchen an die Reliabilität und Validität einer Studie vereinbaren (Stichworte: Messfehler, Inhaltsvalidität). Es ist daher ein kritischer Blick auf die Operationalisierung der interessierenden Variablen der jeweiligen Online-Befragungen zu werfen. Werden Informationen zu Reliabilität und Validität der Messungen berichtet? Der Review zeigt, dass dies in nur 2 der 97 Befragungen geschieht.

#### Multi-Informant Studies

Die Aussagekraft von Online-Befragungen kann erhöht werden, indem dieselben Items von mehreren Personengruppen (z. B. Schüler*innen, Lehrer*innen) aus deren jeweils spezifischer Perspektive eingeschätzt werden. Manche der hier gelisteten Online-Befragungen nutzten die Möglichkeit, interessierende Aussagen aus unterschiedlichen Informationsquellen einschätzen zu lassen, um so die Validität der Befunde zu stärken. Der vorliegende Review zeigt an vielen Stellen, dass die Perspektiven der drei Akteursgruppen (Schüler*innen, Eltern, Lehrkräfte) teils deutlich auseinandergehen. Bei der Interpretation von Informationen, die nur auf einer Akteursgruppe basieren, ist daher immer die perspektivenspezifische Validität zu berücksichtigen.

#### Limitationen des Reviews

Auch der vorliegende Review selbst muss kritisch reflektiert werden.

##### Fokus auf deutschsprachige Länder

Der vorgelegte Review fokussiert bewusst auf den DACH-Raum, da ein internationaler Review aus mehreren Gründen kaum machbar gewesen wäre. (1) Allein für den DACH-Raum liegen 97 Befragungen vor, die gesichtet und analysiert werden mussten. Es ist anzunehmen, dass international gesehen bereits eine nicht handhabbare Fülle an Befragungen vorliegt. (2) Viele Befragungen, an denen wir interessiert sind, werden nicht in englischer Sprache publiziert, da es sich nicht um wissenschaftliche Befragungen handelt, die ein internationales Publikum ansprechen, sondern für die Region und/oder Nation erstellt wurden. Daher sind derartige Befragungen für uns bereits sprachlich nicht zugänglich. (3) Der DACH-Raum stellt einen vergleichsweise einheitlichen Kulturkreis dar, der aufgrund seiner gemeinsam verwendeten Sprache (sieht man von einzelnen Kantonen in der Schweiz ab), aber auch der Ähnlichkeit der Bildungssysteme, unserer Einschätzung nach ausreichend homogen ist, um die Befragungen in einer gemeinsamen Analyse zu untersuchen. Zwar wäre ein komparativer internationaler Vergleich durchaus interessant, um zu erörtern, wie unterschiedliche Bildungsregionen mit den Schulschließungen umgegangen sind (z. B. TV-Unterricht in China); dies war aber nicht Ziel des vorliegenden Reviews. Vielmehr soll der vorliegende Review den Forschungsstand zu Befragungen darstellen, die die Situation während des Schul-Lockdowns beschreiben, um so für weitere Studien im deutschsprachigen Raum eine empirische Basis zu schaffen.

##### Interpretation der Befunde

Mangels repräsentativer Vergleichsdaten ist häufig schwer zu beurteilen, ab wann die in den Befragungen berichteten relativen Häufigkeitsverteilungen (z. B. der Anteil zustimmender Proband*innen) als „viel“ oder „wenig“ zu interpretieren sind. Hinzu kommt, dass sich in den Befragungen nicht nur die Itemformulierungen unterscheiden, sondern auch die Antwortoptionen. So verwenden nicht alle Studien eine Mittelkategorie – ganz wenige überhaupt drei Kategorien.

##### Inhaltlicher Fokus

Schließlich vernachlässigt der Review Befunde zu Aspekten, die nicht den beiden Modellen (Phasen des Forschungsprozesses, integratives Distance Education-Modell) zuordenbar sind (z. B. die erlebte Belastung der Akteursgruppen während der Schulschließung).

##### Methodologischer Fokus

Darüber hinaus werden ausschließlich quantitative Befragungen berücksichtigt. Da bereits eine Vielzahl qualitativer Studien vorliegt (z. B. Huber et al. 2020; Jesacher-Rößler und Klein 2020; Letzel et al. 2020; Schwab et al. 2020; Wacker et al. 2020) bzw. in Arbeit ist (Hascher et al. 2020), besteht der Bedarf eines entsprechenden Reviews, um der Forderung nach Berücksichtigung des aktuellen Wissensstandes auch umfänglich gerecht zu werden.

Trotz möglicher Limitationen in den bisherigen Online-Befragungen und des vorgelegten Reviews liefert die hier berichtete Synopse ein umfassendes Bild der Schulsituation während des Lockdowns im Frühjahr 2020.

### Implikationen für Forschung und Praxis

Aus dem Review lassen sich einige Implikationen für die Forschung und die Praxis ableiten.

#### Forschung, z. B.

- Die hohe Heterogenität in den Befunden wirft die Fragen nach möglichen Ursachen auf (z. B. Befragungsgruppe, Zusammensetzung der Stichprobe, unterschiedliche Messinstrumente, Erhebungszeitpunkt), die Gegenstand künftiger Forschung sein sollten.
- Bisher fehlen (mit wenigen Ausnahmen) Unterschieds- (z. B. nach dem Schultyp, nach zentralen Schülergruppen) und Zusammenhangsanalysen (z. B. zwischen den Variablengruppen des integrativen Distance Education-Modells), die versuchen, die Varianz in den Daten zu erklären.
- Es fehlen längsschnittliche Studien und – mit zwei Ausnahmen – Studien mit standardisiert erfassten Leistungsdaten, die kausale Schlüsse über bspw. die Entwicklung der Schülerleistungen (insbesondere was den vielzitierten Schereneffekt betrifft) erlauben.
- Insbesondere fehlt auch eine Zusammenschau von qualitativen Befunden, die einen tieferen Blick auf die Situation einzelner Schüler*innen, Eltern und Lehrkräfte werfen.

#### Praxis der Lehrpersonen, z. B.

- Die Befundlage zeigt, dass während der Schulschließungen kaum digital unterstützter Unterricht durchgeführt wurde. Auch zeigt sich, dass viele Lehrkräfte in ihren digitalen Kompetenzen an die Grenzen stoßen. Die Bildungspolitik, -praxis und die Lehrer(fort)bildung sind gefordert, hier die nötigen Kompetenzen und Motivationen für den Einsatz digitaler Medien im Unterricht zu fördern.
- Zudem ist es nötig, die Selbstständigkeit der Schüler*innen und deren Kompetenzen für das selbstregulierte Lernen bedeutend zu fördern, da sich diese im Fernunterricht als besonders prädiktiv für Schüleroutcomes erwiesen haben (Blume et al. 2020; Huber und Helm 2020b).

#### Praxis der Eltern, z. B.

- Auf Seiten der Eltern gilt es insbesondere Mütter in prekären Verhältnissen (alleinerziehend, mit mehreren Kindern, mit einer Erwerbstätigkeit, die kaum Zeit für die Betreuung der eigenen Kinder erlaubt, bildungsfern, mit geringen Deutschkenntnissen, mit finanziellen Schwierigkeiten etc.) bspw. durch finanzielle Ressourcen zu unterstützen.
- Schulen sollten Eltern unterstützen, indem klare Informationen geliefert werden und Beratung bei der (inhaltlichen) Betreuung ihrer Kinder angeboten wird.

## Supplementary Information







## Footnotes

1 1. Theory of Independent Study; 2. Theory of Industrialization of Teaching; 3. Theories of communication and diffusion; 4. Theory of Interaction and Communication; 5. Theory of transactional distance; 6. Equivalency Theory.

2 Die Theorien 2, 3 und 6 dagegen beschreiben Aspekte der Distance Education, die unserer Einsicht nach nicht relevant für den vorliegenden Review sind (z. B. Maximierung und Ökonomisierung des Unterrichts) und werden daher nicht weiter thematisiert.

3 Darüber hinaus fließen Annahmen zur Mehrebenen-Logik und zur Domänenspezifität in das Modell ein.

4 Für den deutschsprachigen Raum ist uns nur die Studie von Tomasik et al. (2020) bekannt, die auf Basis von objektiven Leistungstests die Lernentwicklung von Primar- und Sekundarstufenschüler*innen der Schweiz während der Schulschließung untersucht. International liegen dazu vergleichbare Leistungsstudien für Belgien von Maldonado und de Witte (2020) und für die Niederlande von Engzell et al. (2020) vor.

5 Die diesem Review zugrundliegenden Berichte können beim Erstautor angefragt werden.

6 Wir verwenden den Begriff „Fälle“, da wir nicht garantieren können, dass die Stichproben disjunktiv sind. Es kann sein, dass Personen in mehreren Befragungen teilgenommen haben.

7 https://deutsches-schulportal.de/bildungswesen/schuloeffnung-das-haben-die-laenderchefs-entschieden/ (28.08.2020).

8 Erste Längsschnittanalysen aus Huber et al. (2020) wurden an der wels.edulead.net-Tagung vorgestellt.

9 Bei Berghammer (2020) und Wößmann et al. (2020) wurde zusätzlich mit gewichteten Daten gearbeitet.

10 Für die meisten der angeführten Befragungen (Bildungsdirektion Nidwalden 2020; Cecchini und Dutrévis 2020; Grote 2020; Letzel et al. 2020; Porsch und Porsch 2020; Rathgeb 2020; Schober et al. 2020a; Thies und Klein 2020; Wacker et al. 2020) ist nicht klar, ob auf eine 5‑ oder 7‑Tage-Woche Bezug genommen wird. Einige wenige Befragungen verwenden als Bezugsrahmen „an typischen Schultagen“ (Anger et al. 2020; Holtgrewe et al. 2020a; Tengler et al. 2020; Wößmann et al. 2020)*.* Manche Befragungen fragen direkt nach dem wöchentlichen Lernaufwand (Heller und Zügel 2020; Huber et al. 2020; Refle et al. 2020).

11 Auch bei Schober et al. (2020a) wird der täglich investierte Lernaufwand aus Schülersicht berichtet. Allerdings wurde hier als unterste Kategorie „täglich 3,5 h und weniger“ gewählt, der ein Viertel der befragten Schüler*innen angehört.

12 Standardabweichung: 2,13 h.

13 Standardabweichung: 15,38 h.

14 Demgegenüber steht eine Gruppe von 10–25 % der Schüler*innen, die angeben, diese Skills nicht zu beherrschen.

15 Bei Schreiner et al. (2020) kann allerdings kein Zusammenhang zwischen dem Bildungsabschluss der Eltern und der selbständigen Bewältigung der Aufgaben im Fernunterricht durch die Schüler*innen festgestellt werden. Dagegen kamen jene Schüler*innen, die Deutsch nicht als Erstsprache haben, weniger gut mit der selbständigen Bewältigung der Anforderungen zurecht.

16 In der Befragung von Letzel et al. (2020) geben allerdings nur 10 % der Schüler*innen an, dass sie sich mehr Unterstützung von den Eltern wünschen würden. Diese Diskrepanz könnte am hohen Durchschnittsalter der befragten Schüler*innen von 15 Jahren liegen.

17 Grote (2020) stellt einen Ausreißer dar: Etwa 15 % haben nie Zeit.

18 Weitere 27 % wenden bis zu 2 h; 25 % bis zu 3 h; 14 % bis zu 4 h und 6 % bis zu 5 h pro Tag auf.

19 Aus der Ergebnisdarstellung bei Heller und Zügel (2020) geht nicht hervor, ob es sich um Stunden pro Tag oder Woche handelt. Es ist allerdings letzteres anzunehmen, da die Kategorie „mehr als 8 h“ von 35 % der Eltern gewählt wurde.

20 Das Eltern grundsätzlich eher zufrieden mit der Unterstützung der Lehrkräfte sind, zeigt bspw. die hohe durchschnittliche Elternzufriedenheit mit der Lehrerunterstützung bei Andresen et al. (2020).
